# Precision Phenotyping of Dilated Cardiomyopathy Using Multidimensional Data

**DOI:** 10.1016/j.jacc.2022.03.375

**Published:** 2022-06-07

**Authors:** Upasana Tayal, Job A.J. Verdonschot, Mark R. Hazebroek, James Howard, John Gregson, Simon Newsome, Ankur Gulati, Chee Jian Pua, Brian P. Halliday, Amrit S. Lota, Rachel J. Buchan, Nicola Whiffin, Lina Kanapeckaite, Resham Baruah, Julian W.E. Jarman, Declan P. O’Regan, Paul J.R. Barton, James S. Ware, Dudley J. Pennell, Bouke P. Adriaans, Sebastiaan C.A.M. Bekkers, Jackie Donovan, Michael Frenneaux, Leslie T. Cooper, James L. Januzzi, John G.F. Cleland, Stuart A. Cook, Rahul C. Deo, Stephane R.B. Heymans, Sanjay K. Prasad

**Affiliations:** a National Heart Lung Institute, Imperial College London, London, United Kingdom; b Royal Brompton Hospital (Guy’s and St Thomas’s NHS Foundation Trust), London, United Kingdom; c Cardiovascular Research Institute Maastricht (CARIM), Maastricht University Medical Center, Maastricht, the Netherlands; d Department of Clinical Genetics, Maastricht University Medical Center, Maastricht, the Netherlands; e Department of Medical Statistics, London School of Hygiene and Tropical Medicine, London, United Kingdom; f National Heart Centre, Singapore; g Medical Research Council London Institute of Medical Sciences, Imperial College London, London, United Kingdom; h Mayo Clinic, Jacksonville, Florida, USA; i Cardiology Division, Massachusetts General Hospital, Baim Insitute for Clinical Research, Boston, Massachusetts, USA; j One Brave Idea and Division of Cardiovascular Medicine, Brigham and Women’s Hospital, Boston, Massachusetts, USA; k Centre for Molecular and Vascular Biology, Department of Cardiovascular Sciences, KU Leuven, Belgium

**Keywords:** heart, machine learning, proteomics, CMR, cardiovascular magnetic resonance, DCM, dilated cardiomyopathy, hsTnI, high sensitivity troponin-I, IL4RA, interleukin 4 receptor alpha, LBBB, left bundle branch block, LGE, late gadolinium enhancement, NT-proBNP, N-terminal pro–B-type natriuretic peptide, NYHA, New York Heart Association, PCA, principal component analysis

## Abstract

**Background:**

Dilated cardiomyopathy (DCM) is a final common manifestation of heterogenous etiologies. Adverse outcomes highlight the need for disease stratification beyond ejection fraction.

**Objectives:**

The purpose of this study was to identify novel, reproducible subphenotypes of DCM using multiparametric data for improved patient stratification.

**Methods:**

Longitudinal, observational UK-derivation (n = 426; median age 54 years; 67% men) and Dutch-validation (n = 239; median age 56 years; 64% men) cohorts of DCM patients (enrolled 2009-2016) with clinical, genetic, cardiovascular magnetic resonance, and proteomic assessments. Machine learning with profile regression identified novel disease subtypes. Penalized multinomial logistic regression was used for validation. Nested Cox models compared novel groupings to conventional risk measures. Primary composite outcome was cardiovascular death, heart failure, or arrhythmia events (median follow-up 4 years).

**Results:**

In total, 3 novel DCM subtypes were identified: profibrotic metabolic, mild nonfibrotic, and biventricular impairment. Prognosis differed between subtypes in both the derivation (*P <* 0.0001) and validation cohorts. The novel profibrotic metabolic subtype had more diabetes, universal myocardial fibrosis, preserved right ventricular function, and elevated creatinine. For clinical application, 5 variables were sufficient for classification (left and right ventricular end-systolic volumes, left atrial volume, myocardial fibrosis, and creatinine). Adding the novel DCM subtype improved the C-statistic from 0.60 to 0.76. Interleukin-4 receptor-alpha was identified as a novel prognostic biomarker in derivation (HR: 3.6; 95% CI: 1.9-6.5; *P =* 0.00002) and validation cohorts (HR: 1.94; 95% CI: 1.3-2.8; *P =* 0.00005).

**Conclusions:**

Three reproducible, mechanistically distinct DCM subtypes were identified using widely available clinical and biological data, adding prognostic value to traditional risk models. They may improve patient selection for novel interventions, thereby enabling precision medicine.

Dilated cardiomyopathy (DCM) is the leading indication for heart transplantation and a common cause of heart failure. The diagnosis of DCM is a downstream, imaging-based, homogenous phenotypic classification based on abnormalities in cardiac structure and function. Yet, it is a disease with heterogeneous etiologies (eg, genetic, environmental), clinical manifestations (eg, heart failure, arrhythmia), comorbidities, and response to therapeutic interventions. Despite considerable improvements in disease classification and characterization,^1^, ^2^, ^3^ DCM is associated with an average 5-year mortality of about 20%.^4^^,^^5^ A key unmet need in DCM is to define the underlying disease with greater precision to either target existing therapies more effectively or to identify distinct pathophysiological mechanisms that may be amenable to novel therapies targeted to the patient subgroups most likely to benefit.

Parallel to this issue, for clinicians there is an unmet need to understand how to utilize the growing volume and complexity of clinical data to guide patient care. These include the increasing availability of genetic profiling, advanced imaging, and proteomic data. Novel approaches to data science such as machine learning may help, but have been hindered by studies with poor reproducibility and no validation.^6^, ^7^, ^8^, ^9^

In this study, we applied machine-learning approaches to multiparametric phenotyping to define and validate novel prognostically relevant DCM disease subtypes that could facilitate stratified therapy. This approach harnesses a breadth of clinical, imaging, proteomic, and genetic data and makes it clinically accessible and relevant to improve disease characterization and patient stratification. We hypothesized that machine learning approaches would identify unique groupings—or clusters—of patients with DCM with characteristic patterns of risk factors, cardiac manifestations, and outcomes. We further validated our findings in a separate, distinct patient population to explore the portability of our findings.

## Methods

### Study cohort and multiparametric phenotyping

The derivation cohort comprised 426 patients with a clinical diagnosis of DCM confirmed by late gadolinium enhancement (LGE) cardiovascular magnetic resonance (CMR) prospectively enrolled in the National Institute for Health Research Royal Brompton Hospital Cardiovascular Biobank project between 2009 and 2015. The cohort underwent detailed clinical, imaging, genetic, and biomarker phenotyping. All patients provided written informed consent. The study was approved by the regional ethics committee (South Central Research Ethics Committee 19/SC/0257).

DCM was diagnosed based on established CMR criteria of left ventricular dilation and reduced ejection fraction with reference to age- and sex-adjusted nomograms^10^ in the absence of known coronary artery disease (presence of subendocardial LGE suggestive of previous myocardial infarction, >50% stenosis in ≥1 major epicardial coronary arteries, or need for previous percutaneous coronary intervention or coronary artery bypass grafting), abnormal loading conditions (uncontrolled hypertension or significant primary valvular disease), toxin exposure (alcohol consumption in excess of 80 g/d for 5 years meeting criteria for alcoholic cardiomyopathy), systemic disease known to cause DCM, pericardial disease, congenital heart disease, infiltrative disorders (eg, sarcoidosis), recent acute presentation of myocarditis, or significant primary valvular disease. Diabetes or a history of well-controlled hypertension were documented as comorbidities. A contraindication to CMR included the presence of a pacemaker, defibrillator, pacing wires, metal implants (including cochlear or spinal implants, hydrocephalus shunts), vascular clips, or foreign bodies or metal in the eye. All patients had clinical screening at recruitment to the study as previously described.^11^^,^^12^ At enrollment, all study participants underwent the following: 1) CMR for assessment of cardiac chamber volumes and function and assessment of fibrosis (1.5-T, Siemens Sonata or Avanto scanners, Siemens Medical Systems); 2) analysis of 276 biomarkers putatively linked to cardiovascular disease on 3 commercially available immunoassay panels (Proseek Multiplex CVD II, CVD III, Inflammation; Olink Bioscience; listed in the Supplemental Appendix); 3) quantification of serum high-sensitivity troponin I and serum creatinine; and 4) targeted cardiac genetic analysis of DCM genes from a 169 cardiac gene panel (TruSight Cardio Sequencing kit, Illumina) (further described in the Supplemental Appendix). Truncating variants in the titin gene were curated as previously described.^13^ Genetic variants were grouped into 4 classes: truncating variants in the titin gene, *LMNA*, other sarcomeric variants, or other DCM variant.

The validation cohort comprised 239 individuals with a clinical diagnosis of DCM confirmed by LGE-CMR prospectively enrolled in the Maastricht Cardiomyopathy Registry from the Maastricht University Medical Center between 2009 and 2016. The cohort underwent the same clinical, imaging, genetic, and biomarker phenotyping as the derivation cohort (Supplemental Appendix). All patients provided written informed consent. The study was approved by the local Ethical Review Board of the Maastricht University Medical Center (METC 12-04-013).

A comparator control cohort for Olink biomarker analysis comprised 51 healthy individuals. Further details are provided in the Supplemental Methods.

### Data preprocessing

All available variables for clustering (clinical, imaging, genetic, proteomic) were considered. This comprised demographic (age, sex, and race), clinical (diabetes mellitus, hypertension), electrocardiographic (resting heart rate, atrial fibrillation, left bundle branch block), and 11 CMR variables (left and right ventricular: ejection fraction, indexed end-diastolic, end-systolic, and stroke volumes; indexed left ventricular mass; indexed left atrial volume; and late-gadolinium enhancement midwall fibrosis), as well as variants in 12 curated DCM genes (*TTN*, *LMNA*, *MYH7*, *TNNT2*, *VCL*, *TPM1*, *TNNC1*, *RBM20*, *DSP*, *BAG3*, *SCN5A*, and *TCAP*), and 278 proteomic markers (biomarker panels as outlined in Supplemental Table 5 plus troponin and creatinine). Missing values were imputed with the SVDImpute function within the *imputation* package in R (R Foundation for Statistical Computing) (variables with missing data are outlined in the Supplemental Appendix). Principal component analysis (PCA) was performed as a means of dimensionality reduction for both biomarker and CMR data separately (PCA was not performed on demographic or clinical variables). This approach, rather than a single combined PCA, was used for several reasons. First, the PCA comprised 276 biomarker variables, but only 11 CMR variables. When performing a combined PCA, the overwhelming majority of the variance reduction (and therefore contribution to the final model) was driven by the biomarker components, with the CMR variables contributing relatively little. Second, 97% of the CMR variance was accounted for using only 6 PCA components. In contrast, only 47% of the variance was explained using 5 biomarker components, and this was not improved by combining the PCAs; 10 components explained only 55% of the variance in the combined analysis. Finally, separate PCAs better allowed us to delineate the radiomic vs biochemical contributions to the final model. All available additional clinical variables were used for clustering and are listed in Table 1. Further details about the biomarker and CMR PCA are explained in the Supplemental Methods. PCA loadings for the biomarker and CMR analysis are shown in Supplemental Tables 9 and 10.

**Table 1:** Distribution of Clinical Variables Used for Bayesian Model Clustering in the Derivation Cohort

	Derivation Cohort (n = 426)	Validation Cohort (n = 239)	*P* Value
Clustering variables (biomarker and CMR principal component loadings not shown)			
Age, y	54 (44-64)	56 (46-64)	0.20
Men	287 (67)	154 (64)	0.44
European	378 (89)	231 (97)	<0.001
Diabetes	48 (11)	27 (11)	1.00
Hypertension	123 (29)	99 (41)	0.001
Left bundle branch block	120 (28)	58 (24)	0.32
Atrial fibrillation	105 (25)	53 (22)	0.51
Heart rate, beats/min	74 (16)	74 (17)	0.51
Creatinine, μmol/L	79 (68-95)	89 (75-103)	<0.001
*TTN* truncating variant	54 (13)	16 (13)	1.00
CMR variables			
Left ventricular ejection fraction, %	41 (30-51)	35 (24-44)	<0.001
Indexed left ventricular end-diastolic volume, mL/m^2^	116 (102-142)	123 (101-162)	0.05
Indexed left ventricular end-systolic volume, mL/m^2^	68 (51-95)	79 (58-119)	<0.001
Indexed left ventricular stroke volume, mL/m^2^	48 (39-56)	42 (32-52)	<0.001
Indexed left ventricular mass, g/m^2^	86 (72-103)	71 (57-88)	<0.001
Right ventricular ejection fraction, %	54 (44-62)	48 (38-56)	<0.001
Indexed right ventricular end-diastolic volume, mL/m^2^	85 (69-101)	83 (67-101)	0.51

Values are median (IQR) or n (%). Comparative data are shown for the validation cohort. The second part of the table shows the cardiovascular magnetic resonance (CMR) phenotypic variables in the primary and derivation cohort. Data are compared using the Mann-Whitney test for continuous data and the Fisher test for categorical data. Principal component loadings of protein biomarkers and CMR phenotypic variables are not shown.

### Profile regression mixture models for patient grouping

The PReMiuM R Package was used to perform profile regression mixture modeling for patient grouping (further explained in the Supplemental Methods). The inputs described in the data preprocessing section were used, and a categorical response variable (New York Heart Association [NYHA] functional class I, II, or III/IV) was entered as an outcome. A range of seeds were used to assess the robustness of the results. In contrast to prior work evaluating phenomapping in heart failure,^14^ this semisupervised approach was selected to minimize the high variability previously observed with model-based clustering assignments depending on the choice of input features. NYHA functional class was used to create clinically relevant groupings instead of groupings that were driven by a less clinically meaningful feature. As a comparator, unsupervised clustering was employed using the same input data (results outlined in Supplemental Table 1), demonstrating a high variability in groupings depending on the clustering algorithm chosen and without different survival profiles.

### Mapping of validation cohort to phenotypic groups using multinomial logistic regression

The purpose of the validation set was to demonstrate the utility of the clusters. In an effort to validate phenotypic groups identified in the derivation set, a model was developed to assign patients in the validation cohort to the 3 phenotypic groups identified in the derivation cohort using a minimal number of input variables. The glmnet package in R was used, and a multinomial logistic regression model was fitted with an L1 penalty, using all shared clinical and biomarker variables as potential inputs. The resulting model was 97% accurate on the derivation cohort as evaluated through 10-fold cross-validation and used only 5 variables: indexed left ventricular end-systolic volume, indexed right ventricular end-systolic volume, indexed left atrial volume, midwall myocardial fibrosis detected on late gadolinium enhancement imaging (LGE), and serum creatinine. We used the corresponding inputs from the validation cohort and mapped patients to the 3 phenotypic groups.

### Survival analysis across the phenotypic groups

The primary endpoint in the derivation and validation cohorts was a composite of cardiovascular mortality, major arrhythmic events, and major heart failure events (explained in detail in the Supplemental Methods). All primary endpoint events were adjudicated by an independent committee of 3 senior cardiologists (M.F., J.J., R.B.) with expertise in electrophysiology, heart failure management, or clinical trial adjudication who were blinded to imaging and biomarker data. All patients had follow-up data. The median follow-up duration was 4.0 years (IQR: 2.1-5.8 years) in the derivation cohort and 3.1 years (IQR: 1.7-5.0 years) in the validation cohort. The log-rank test was used to examine the survival of the 3 phenotypic groups in derivation and validation cohorts, and the cph function within the rms package in R^15^ was utilized to perform survival analysis using a Cox Proportional Hazards model. Nested models—ie, including or not including the phenotypic cluster assignments—were compared using a likelihood ratio test.

### Analysis of biomarker variable importance in the random forest algorithm

A random survival forest model with all biomarker features treated as individual features (ie, not combined within principal components) was created. To assess the importance of individual features, variable importance and minimal tree depth was computed using the gg_vimp and gg_md functions, respectively, in the ggRandomForest package.^16^ Cox proportional hazard modelling was used to evaluate a novel prognostic biomarker.

**Figure 1:**
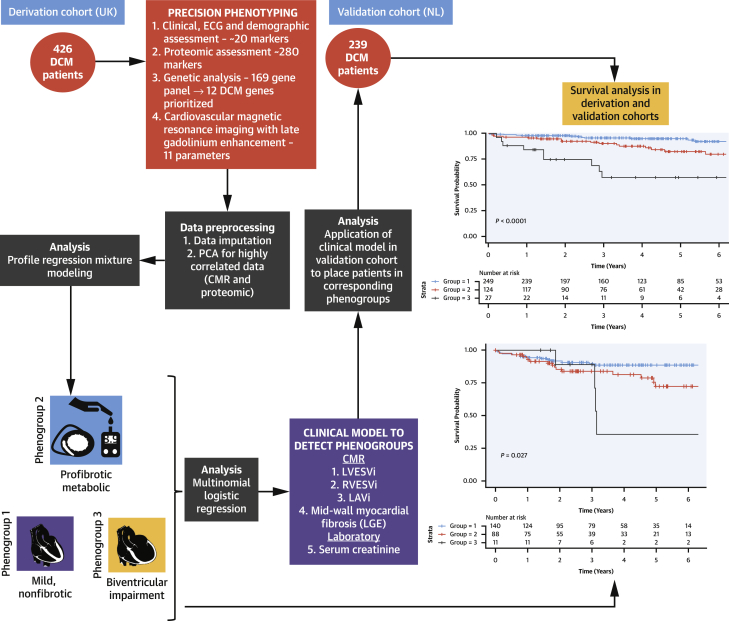
An Overview of the Study Analysis Pipeline Machine learning approaches were applied to multiparametric data (clinical, imaging, genetics, biomarkers) from a prospectively recruited UK derivation cohort of patients with dilated cardiomyopathy (DCM) and identified 3 novel reproducible subtypes of disease: mild nonfibrotic, profibrotic metabolic, and biventricular impairment. Multinomial logistic regression was used to create a model to place patients in the independent Dutch validation cohort into corresponding subtypes. Composite survival differed between novel subtypes in both the derivation and validation cohorts. CMR = cardiovascular magnetic resonance; ECG = electrocardiogram; LAVi = indexed left atrial volume; LGE = late gadolinium enhancement; LVESVi = indexed left ventricular end-systolic volume; NL = the Netherlands; PCA = principal component analysis; RVESVi = indexed right ventricular end systolic volume; UK = United Kingdom.

All statistical analyses were conducted in the R environment (version 3.3.1). An overview of the analysis pipeline is provided in Figure 1.

## Results

### Cohort demographics

The UK derivation cohort consisted of 426 patients with confirmed DCM. Most were European (n = 378; 89%), 287 were men (67%), and most were in NYHA functional class I/II (n = 339; 80%) at the time of recruitment. Median age at recruitment was 54 years (IQR: 44-64 years). Cardiac morphology and function are shown in Table 1. There was moderate-severe left ventricular impairment with a median left ventricular ejection fraction of 41%. Midwall myocardial fibrosis was present in 137 patients (32%), 120 (28%) had left bundle branch block (LBBB), 123 (29%) had controlled hypertension, and 48 (11%) had diabetes mellitus.

The Dutch validation cohort consisted of 239 patients with confirmed DCM. Most were European (n = 231; 97%), 154 were men (64%), and most were in NYHA functional class I/II (n = 174; 72.8%) at recruitment. The median age at recruitment was 55 years (IQR: 47-64 years). Cardiac morphology and function are shown in Table 1. There was slightly more ventricular impairment in the validation cohort with a median left ventricular ejection fraction of 35%. Midwall myocardial fibrosis was present in 91 patients (38%), 58 (24%) had LBBB, 99 (41%) had controlled hypertension, and 27 (11%) had diabetes mellitus. The commonest genetic finding was a truncating variant in *Titin*, found in 13% of patients in both the derivation and validation cohorts (Table 1).

### Defining DCM subtypes in the derivation cohort

Following data preprocessing, 400 study participants remained in the derivation cohort. We clustered these 400 individuals in the derivation cohort using a total of 21 variables comprising 6 CMR-PCA vectors, 5 biomarker-PCA vectors, and 10 clinical variables comprising age, sex, race, diabetes, hypertension, LBBB, atrial fibrillation, creatinine, heart rate and the presence of a *Titin* truncating variant (Table 1), with NYHA functional class as an outcome variable. The optimal number of DCM clusters was three, a value robust to multiple runs of profile regression mixture modeling. These 3 clusters, henceforth referred to as phenotypic groups (PGs), comprised 63% (n = 249), 31% (n = 124), and 7% (n = 27) of the cohort, respectively (Table 2).

**Table 2:** Comparison of Phenotypic Variables Across Patient Groups in the Derivation Cohort

	Group 1 (n = 249) Mild, Nonfibrotic	Group 2 (n = 124) Profibrotic Metabolic	Group 3 (n = 27) Biventricular Impairment	*P* Value
Age, y	52 (44-63)	56 (46-66)	55 (46-66)	0.08
European	88	90	89	0.84
Men	59	85	82	<0.00001
Hypertension	25	35	40	0.04
Body surface area	2.0 (1.8- 2.1)	2.1 (1.9-2.2)	1.9 (1.8-2.0)	0.01
Diabetes mellitus	9	20	4	0.002
Atrial fibrillation	23	27	33	0.24
Ventricular tachycardia (sustained) before enrollment	0.4	5	0	0.007
Nonsustained ventricular tachycardia before enrollment	9	12	22	0.11
Excess alcohol consumption	14	18	22	0.19
Left bundle branch block	28	23	44	0.11
Heart rate, beats/min	74 (62-85)	73 (63-80)	81 (70-92)	0.045
Family history of dilated cardiomyopathy	18	13	19	0.43
Family history of sudden cardiac death	19	12	7	0.11
TTNtv	13	13	11	0.96
*LMNA*	0.4	0.8	0	0.81
Other sarcomeric/any DCM genetic variant	2	0.8	0	0.67
NYHA functional class				<0.00001
I	122 (49)	53 (43)	5 (19)	
II	95 (38)	55 (44)	9 (33)	
III/IV	32 (13)	16 (13)	13 (48)	
Midwall myocardial fibrosis/LGE	0	100	22	<0.00001
Creatinine, μmol/L	81 (68-100)	99 (81-120)	100 (83-130)	<0.00001
Beta-blocker	68	81	70	0.03
ACE inhibitor	74	90	89	0.002
Aldosterone blocker	26	48	56	<0.00001
Diuretic	35	54	82	<0.00001
Left ventricular ejection fraction, %	45 (35-52)	37 (28-47)	21 (18-28)	<0.00001
Right ventricular ejection fraction, %	55 (46-62)	54 (43-62)	30 (25-44)	<0.00001
Indexed left atrial volume, mL/m^2^	53 (43-66)	59 (47-74)	93 (71-130)	<0.00001
Indexed left ventricular end-diastolic volume, mL/m^2^	110 (100-130)	120 (110-150)	200 (160-220)	<0.00001
Indexed left ventricular end-systolic volume, mL/m^2^	61 (50-82)	75 (57-110)	150 (120-170)	<0.00001
Indexed left ventricular stroke volume, mL/m^2^	49 (41-57)	47 (38-56)	42 (28-51)	0.03
Indexed left ventricular mass, g/m^2^	83 (68-97)	97 (79-110)	110 (85-120)	<0.00001
Indexed right ventricular end diastolic volume, mL/m^2^	83 (69-99)	84 (68-100)	120 (88-140)	0.00011
Indexed right ventricular end systolic volume, mL/m^2^	39 (29-50)	38 (28-51)	82 (48-100)	<0.00001
Indexed right ventricular stroke volume, mL/m^2^	45 (37-53)	44 (35-55)	37 (31-44)	0.011
High-sensitivity troponin-I, ng/mL	2.8 (1.1-6.6)	8.9 (4-15)	14 (7-25)	<0.00001
NT-proBNP, NPX	1.9 (0.89-3.2)	2.8 (1.4-3.9)	4.4 (3.7-5.7)	<0.00001
IL4RA, NPX	2.6 (2.3-2.9)	2.7 (2.4-2.9)	2.9 (2.6-3.4)	0.0004
Interval since diagnosis, y	0.11 (0-0.45)	0.14 (0-0.71)	0.04 (0-1.4)	0.77

Values are median (IQR), %, or n (%). Only biomarkers that were significantly different between dilated cardiomyopathy subtypes are shown. NPX is the arbitrary unit for Olink biomarker assays. It is a log2 scale; therefore, a 1 NPX difference means a doubling of protein concentration. Groups are compared using the Kruskal-Wallis test for nonnormal continuous variables and Fisher test for categorical variables.

ACE = angiotensin-converting enzyme; DCM = dilated cardiomyopathy; IL4RA = interleukin 4 receptor alpha; LGE = late gadolinium enhancement; NPX = normalized protein expression value; NT-proBNP = N-terminal pro–B-type natriuretic peptide; NYHA = New York Heart Association; TTNtv = truncating variant in titin gene.

### Identifying a novel profibrotic metabolic DCM subtype

About 90% of individuals in each cluster were European. Age was similar across clusters (Table 2). There was no difference in the median interval since DCM diagnosis (median: Group 1 0.11 [IQR: 0-0.45], Group 2 0.14 [IQR: 0-0.71], Group 3 0.04 [IQR: 0-1.4]; *P =* 0.77) among groups (Table 2). Key differences between groups are also highlighted in the Supplemental Appendix (Supplemental Table 2, Supplemental Figure 1). There were a number of differences between the groups in terms of fibrosis, metabolic state, and arrhythmia.

PG1 individuals were notable for the highest proportion of women (41%). They had the mildest cardiac phenotype with the least impaired left ventricular ejection fraction, most preserved right ventricular function, and nondilated atria. Members in this group did not have myocardial fibrosis. They had the lowest prevalence of atrial fibrillation, the lowest plasma concentrations of N-terminal pro–B-type natriuretic peptide (NT-proBNP) and high sensitivity troponin-I (hsTnI), and the lowest serum creatinine.

PG2 was a novel profibrotic metabolic subtype of DCM. Individuals in PG2 had the highest rates of diabetes mellitus (20% of patients vs 9% and 4% in PG1 and PG3, respectively; *P =* 0.002). Strikingly, all had midwall myocardial fibrosis. Regarding arrhythmia, 5% of patients had experienced clinically significant ventricular tachycardia compared with 0% in other groups (Table 2). With respect to cardiac structure and function, individuals in PG2 had intermediate values between PG1 and PG3 for several left ventricular measurements (left ventricular ejection fraction, and end-diastolic and -systolic volumes) but similar right ventricular structure and function to PG1. Those in PG2 also had a higher body surface area compared with the other groups.

PG3 members had the most severe cardiac phenotype, essentially representing the opposite extreme to PG1, with biventricular adverse remodeling (enlarged left ventricle, right ventricle, and left atrium), worst NYHA functional class, and highest serum levels of NT-proBNP, hsTnI, and creatinine (Table 2).

There was no difference in a genetic etiology to DCM between the groups including titin truncating variants, *LMNA* variants, or other DCM genetic variants. In addition, the groups did not differ in terms of a family history of DCM or a family history of sudden cardiac death.

In an effort to understand the impact of using NYHA functional class in profile regression (which might have generated groupings that only differed by NYHA functional class) we found PG1 and PG2 had a very similar distribution of NYHA functional class, in contrast to PG3, thus lending further support to the identification of 3 novel subtypes of DCM, independent of NYHA functional class.

### Survival analysis: PGs and outcome

**Figure 2:**
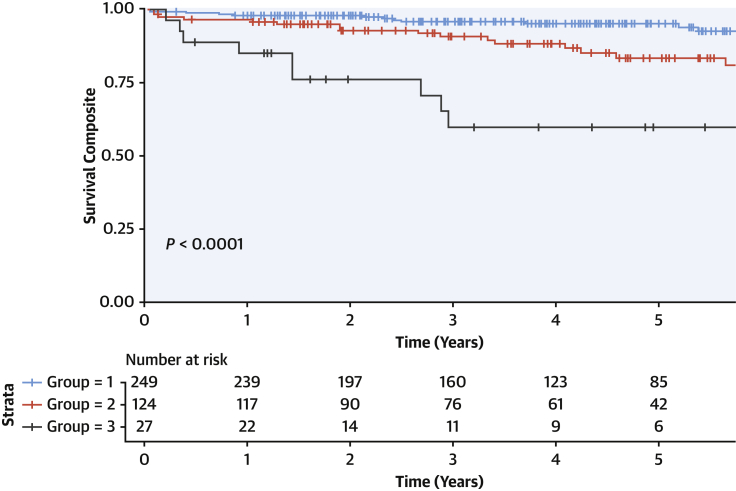
UK Derivation Cohort: Outcome for the 3 Novel Disease Subtypes Phenotypic group 2 (PG2) is a novel distinct profibrotic metabolic subtype of DCM. PG2 patients had the highest rates of diabetes mellitus; all had midwall myocardial fibrosis, and they had experienced more ventricular tachycardia compared with the other groups. PG2 patients had intermediate values between PG1 and PG3 for several left ventricular measurements (left ventricular ejection fraction and end-diastolic and end-systolic volumes) but similar right ventricular structure and function to PG1. Composite survival consists of major arrhythmic events, major heart failure events, or cardiovascular mortality. Outcome varied by these novel DCM disease subtypes. *P* value is computed by the log-rank test.

Prognosis worsened from PG1 to PG2 to PG3 in the derivation cohort (Figure 2); *P* < 0.0001. We compared outcomes between individual PGs using an unadjusted Cox model. All 3 PGs differed from one another: Cluster 1 vs 2 *P =* 0.011, Cluster 1 vs 3 *P <* 0.001; Cluster 2 vs 3 *P =* 0.003. To evaluate whether phenotypic groups added prognostic value for survival beyond conventional measures, a series of nested Cox proportional hazards models were fitted, evaluating changes in model likelihood upon addition of phenotypic groups (Supplemental Appendix, Supplemental Table 3). Addition of a PG term improved the fit of a broad range of multiple models composed of traditional risk factors, suggesting that the phenotypic groups add prognostic value beyond clinical, imaging, and proteomic markers (eg, age, race, sex, left ventricular size and function, family history of sudden death, NT-proBNP, and right ventricular function). Furthermore, the phenogroups provided prognostic information above and beyond the MAGGIC traditional heart failure prognostic score, increasing the C statistic from 0.60 to 0.76 (Supplemental Tables 3 and 4).

### Validation of novel phenotypic groups in independent cohort

**Figure 3:**
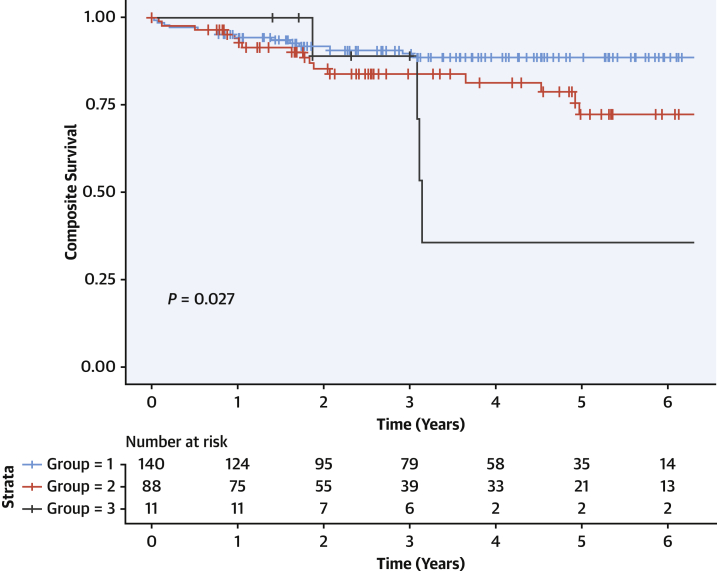
Dutch Validation Cohort: Outcome for the 3 Disease Subtypes The novel disease subtypes in the validation cohort also vary by adverse event risk. Composite survival consists of major arrhythmic events, major heart failure events, or cardiovascular mortality. *P* value is computed by the log-rank test.

Patients in the validation cohort were assigned to phenotypic groups identified in the derivation cohort using a minimal number of input variables (5 variables: indexed left ventricular end-systolic volume, right ventricular end-systolic volume, indexed left atrial volume, LGE, and serum creatinine). A model using only these 5 parameters showed clinical utility: in the independent Dutch validation cohort of 239 DCM patients, the novel phenotypic groups were also associated with prognosis (*P =* 0.027) (Figure 3), resembling the overall prognosis seen in the derivation cohort. This shows the feasibility, potential clinical utility, and validity of the derived phenogroups.

### Identifying IL4RA as a novel prognostic DCM biomarker

**Figure 4:**
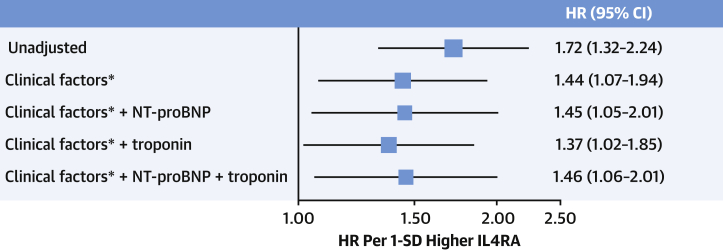
IL4RA Is a Novel Dilated Cardiomyopathy Prognostic Biomarker IL4RA was strongly associated with outcome in both derivation and validation cohorts. Unadjusted and adjusted HRs for IL4RA in the derivation cohort are shown. HRs are presented per 1-SD (ie, standardized to make a fair comparison across biomarkers). In adjusted analyses, IL4RA remained of prognostic utility in addition to clinical factors∗ that predicted outcome (indexed left atrial volume, left ventricular ejection fraction, midwall fibrosis on cardiac magnetic resonance, and a history of nonsustained ventricular tachycardia), as well as in addition to conventional prognostic biomarkers NT-proBNP and high sensitivity troponin I. This suggests that IL4RA is a novel prognostic marker for dilated cardiomyopathy. IL4RA = interleukin 4 receptor alpha; NT-proBNP = N-terminal pro-B-type natriuretic peptide.

Overall, 60 of the 276 biomarkers differed significantly between the novel phenogroups (Supplemental Appendix, Supplemental Table 6). Random survival forests algorithm was used to investigate the importance of these protein biomarkers for prediction of survival. The top predictive feature was serum concentration of interleukin-4 receptor alpha (IL4RA) (Supplemental Figure 2). IL4RA is a transmembrane receptor for interleukins-4 and -13 that is expressed on both innate and adaptive immune cells. It is associated with inflammatory and fibrotic pathways.^17^ IL4RA levels increased from group 1 to group 3 (Table 2). IL4RA was strongly associated with outcome in both the derivation (HR for primary endpoint: 3.6; 95% CI: 1.9-6.5; *P =* 0.00002) and validation cohorts (HR: 1.94; 95% CI: 1.3-2.8; *P =* 0.00005). This suggests that IL4RA is a novel prognostic marker for DCM. In adjusted analyses, IL4RA remained of prognostic utility in addition to clinical factors that predicted outcome in this cohort (indexed left atrial volume, left ventricular ejection fraction, late gadolinium enhancement midwall fibrosis on CMR, and a history of nonsustained ventricular tachycardia), as well as in addition to conventional prognostic biomarkers NT-proBNP and hsTnI (Figure 4). Overall, plasma concentrations of IL4RA were elevated in the DCM cohort compared with controls (DCM NPX median 2.63 [IQR: 2.38-2.93], control NPX median 2.55 [IQR: 2.31-2.72]; *P =* 0.037) (outlined further in Supplemental Methods and Supplemental Table 7).

## Discussion

DCM is a phenotypically homogenous condition with a highly heterogenous etiology that is not currently used to guide management. In this multicenter international study using multiparametric phenomapping, a machine learning approach was used to identify patterns of mechanistically distinct DCM subgroups from familiar clinical and biological data (Central Illustration). Three distinct subgroups of DCM were identified: 1) a mild, nonfibrotic subtype; 2) a novel profibrotic metabolic subtype; and 3) a biventricular impairment subtype. These subtypes were informative for patient stratification and prognosis beyond traditional markers and were reproducible in an independent validation cohort. This complex multiparametric data could be captured in a validation model of 5 parameters that could be easily clinically applicable. These findings may facilitate more targeted approaches to an increasingly diverse repertoire of heart failure therapies and provide an opportunity to identify and better protect DCM patient subgroups at increased risk of mortality and morbidity. The identified subgroups could not be determined with current methods of disease classification. That these novel subgroups were informative for prognosis beyond conventional risk models highlights the value of this type of approach. This is the first study to demonstrate the potential value of machine learning approaches for cardiomyopathy classification over current approaches.

**Central Illustration:**
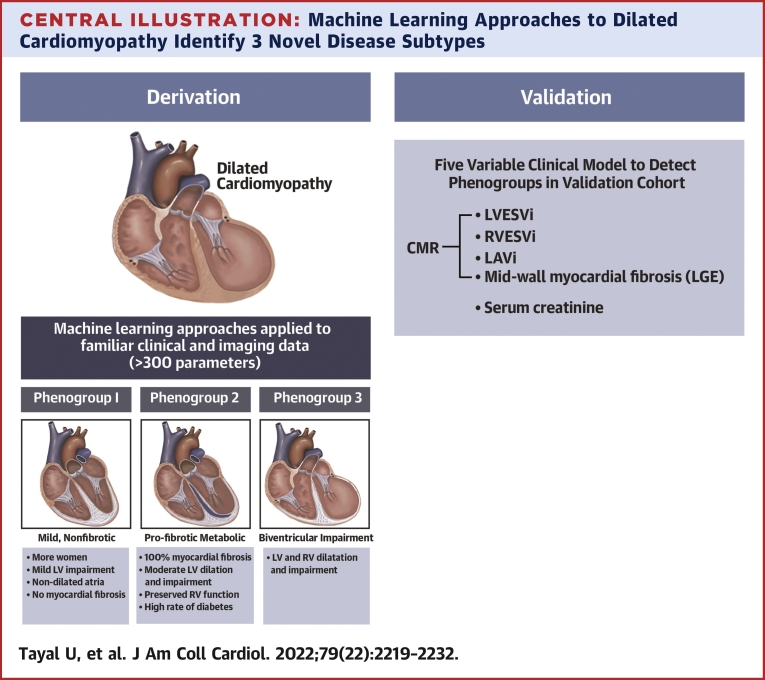
Machine Learning Approaches to Dilated Cardiomyopathy Identify 3 Novel Disease Subtypes Machine learning approaches applied to a prospectively recruited UK derivation cohort of patients with dilated cardiomyopathy identified 3 novel reproducible disease subtypes: mild nonfibrotic, profibrotic-metabolic, and biventricular impairment. Prognosis varied among groups and was reproduced in the independent Dutch validation cohort. The novel profibrotic-metabolic subtype had a high rate of diabetes, universal myocardial fibrosis, elevated creatinine, and preserved right ventricular function. For clinical application, 5 variables were sufficient for classification. CMR = cardiovascular magnetic resonance; LAVi = indexed left atrial volume; LGE = late gadolinium enhancement; LV = left ventricular; LVESVi = indexed left ventricular end-systolic volume; RV = right ventricular; RVESVi = indexed right ventricular end systolic volume.

The novel DCM subtypes identified in this study may be applied in addition to current risk models to improve risk stratification. The addition of the novel subtype term improved the fit of a broad range of prognostic models composed of traditional risk factors showing that the phenotypic groups add prognostic value beyond clinical, imaging, and proteomic markers. In addition, they offer mechanistic insights into DCM, which may underlie differential response to heart failure therapy. Our approach opens the scope to assess whether subtypes of DCM can be matched to specific therapies as a key direction for future research. Strategies to improve patient selection for newer therapies are important to target benefit, improve compliance, and reduce exposure to side effects, and are also important from a cost-benefit perspective.

The identification of a profibrotic metabolic DCM subtype is a key finding of this study. The phenogroups may help to differentiate patients with DCM, particularly in relation to comorbidities that are currently under-recognized in DCM clinical care, in contrast to the management of patients with heart failure and preserved ejection fraction. The profibrotic metabolic subtype 2 was distinct, with universal midwall myocardial fibrosis, a much higher proportion of diabetes, impaired renal function, and relatively preserved right ventricular function, and was more likely to have a prior history of ventricular tachycardia. These findings were not related to age or genetic differences across groups. Subtypes 1 (mild, nonfibrotic group) and 3 (biventricular impairment group) may represent 2 poles of severity within DCM, although they too have distinct clinical characteristics. Subtype 3 was characterized by biventricular impairment but strikingly much less fibrosis compared with the profibrotic metabolic subtype 2. The mild, nonfibrotic subtype 1 was characterized by more asymptomatic milder disease, absence of myocardial fibrosis, right ventricular involvement, or atrial enlargement, and less biomarker derangement. The differential characteristics of myocardial fibrosis and prognosis between the subtypes is striking and highlights the potential of these groupings for clinical impact.

It is well established that midwall myocardial fibrosis is a strong prognostic indicator in DCM,^4^^,^^18^ and it would be reasonable to expect that the proportion of patients with midwall myocardial fibrosis would be highest in subtype 3, the group with marked biventricular impairment, higher plasma concentrations of biomarkers associated with an adverse prognosis (troponin, NT-proBNP), and greater symptom burden. However, only 22% of subtype 3 (biventricular impairment) patients had midwall myocardial fibrosis compared with 100% of subtype 2 (profibrotic metabolic), which could not have been predicted based on disease severity or standard baseline parameters alone. Our approach separated individuals with myocardial fibrosis into 2 distinct groups, with differing cardiac structure and function, clinical characteristics, and crucially, also prognosis. Despite universal myocardial fibrosis, the prognosis was more favorable in the profibrotic metabolic subtype 2 compared with the biventricular impairment subtype 3. Further work is needed to understand why prognosis differs so markedly between these disease subtypes. Appropriate medical therapy may mitigate the potentially adverse prognosis associated with myocardial fibrosis, because patients in the profibrotic metabolic subtype were more likely to be taking beta-blockers, but this requires further study.

An important question remains as to whether the resultant phenotypic groups represent distinct biology or simply different stages of disease. There was no statistically significant difference in interval since diagnosis among subtypes, and all 3 groups had a median time since diagnosis of <2 months. As outlined in the previous text, the 3 subtypes were not easily distinguished by phenotypic severity alone, eg, universal myocardial fibrosis in subtype 2 despite less reduction in left ventricular ejection fraction compared with subtype 3. Therefore, the groupings are less likely to reflect different disease stages. Given the biomarker differences across the groups, we propose that the novel groupings represent biologically distinct groups, and future work should focus on exploring the nature of biological differences between these groups.

In this study, the presence of genetic DCM did not stratify between groups and did not seem to affect prognosis. Titin gene truncating variants, which are not known to confer additional prognostic risk,^11^^,^^19^^,^^20^ were the most common genetic abnormality. There were few patients with variants in arrhythmogenic DCM genes that are known to affect outcome adversely. It could also reflect the importance of the end phenotype, instead of genetic etiology. This clustering algorithm was derived and externally validated in patients on appropriate guideline-directed medical therapy. Therefore, the disease subtypes may also reflect treatment response in that subtype 1 is a cohort that improves in response to therapy, subtype 2 is a metabolic, profibrotic phenotype that could be targeted with specific therapies to improve outcomes after guideline therapy, and subtype 3 is a high-risk advanced phenotype that may need evaluation for advanced heart failure therapies. Our findings were validated in an independent cohort who were not recruited via CMR and who had on average a more impaired cardiac phenotype. Although there are differences between cohorts, the clinical utility of the findings of this study are reflected in identifying similar clusters in an external cohort. Management strategies were similar in both the derivation and validation cohorts; yet, subtype 3 in the validation cohort experienced late-onset adverse events that were not seen in the discovery cohort. This difference in morbidity outcome warrants further investigation, and future work could also evaluate the progression of the model over time in the same patients.

The practical utility of machine learning–generated patient groupings has been difficult to demonstrate, in part because the number of inputs is often too large to find in a replication cohort. To more rapidly translate the findings of this analysis to easily classify individuals with DCM, an approach using multinomial logistic regression was developed whereby a set of only 5 features was required (left ventricular end-systolic volume, right ventricular end-systolic volume, left atrial volume, midwall myocardial fibrosis, and serum creatinine). This is an important and novel insight, demonstrating that, beyond discovery approaches, acquisition of complex multi-omics data may not necessarily improve patient stratification in DCM.

Differences across the novel groupings were evaluated to identify which clinical or biomarker features were most important for DCM outcome. Although other biomarkers and cytokines such as troponin, NT-proBNP, and IL-6 had prognostic value, the top predictive feature of outcomes was serum concentration of interleukin-4 receptor alpha (IL4RA). IL4RA is a transmembrane receptor for interleukins-4 and -13 that is expressed on both innate and adaptive immune cells. It is associated with inflammatory and fibrotic pathways.^10^ IL4-glucocorticoid signaling is finely balanced between stress and immune responses to regulate myocyte proliferation.^21^ IL4RA levels for subtypes 1 and 2 were similar compared with subtype 3, suggesting that it may be a marker of severity, such as a compensated vs noncompensated state, not fibrosis. The role of IL-4 in the development of heart disease is complex, and it has been shown to have both beneficial and adverse effects in preclinical studies.^10^ However, it has not previously been associated with outcomes for heart failure or cardiomyopathy.

### Study limitations

This study comprised 665 participants with DCM who had detailed phenotyping with CMR, genetic analysis, and proteomic study together with complete outcome data, making it one of the most comprehensive and unique data sets of affected individuals. There are, however, a number of potential limitations to this work. This was a predominantly ambulatory cohort of patients with DCM recruited during investigation by CMR, although most were in a worse NYHA functional class at the time of initial presentation before therapy initiation. Our phenomapping classification may not apply to specific subsets of DCM, such as those with very mild disease who may not be referred for specialist investigation or those who are so severe that it prohibits CMR. Our cohort consists of patients seen in our large cardiomyopathy service as well as patients referred to the CMR service for a scan. Within our own institution, patients will undergo a CMR scan as part of their routine standard of care and also before device implantation. In addition, our network of referring hospitals tends to refer patients early on for a CMR scan to look for evidence of myocardial fibrosis and any active inflammation. As such, some patients will have a new diagnosis made in our center, whereas others will have a new diagnosis made elsewhere and then referred either for detailed phenotypic characterization by CMR or for specialist evaluation. However, most patients in this study were enrolled close to the time of DCM diagnosis—which is reflected in the very short median time since diagnosis in all 3 groups. An important consideration and potential confounder is whether the phenotypes identified are distinct or, for instance, represent patients in different stages of their disease process or on medication for different time periods. Our study design was cross-sectional, and future longitudinal studies are planned to address this. In particular, they will aim to address the progression of the model over time in the same patients and to determine whether patients move between subtypes over time. Furthermore, in this study, we did not systematically collect medication duration and class usage over time—this could potentially affect outcomes and should also be addressed in future work.

Although the results of this study are generalizable to other DCM cohorts, an important extension of this work will be to evaluate whether the cardiac volume measurements from echocardiography can be used to classify patients in a similar way and to evaluate the importance of late gadolinium enhancement in the model. Advanced CMR features such as T_1_/T_2_ mapping were not routinely acquired in all patients in this study. Whether these additional features could improve patient classification remains to be evaluated.

Patients were predominantly of European descent. Further work on more diverse cohorts should investigate the effect of ethnicity on phenogroup stratification. We elected not to include medications in the baseline model, because they reflect treatment decisions that are subject to factors other than the disease itself, such as provider bias, patient tolerance, patient preference, and renal function.^22^^,^^23^ Moreover, many patients were studied within days or weeks of diagnosis at a time when their treatment was rapidly changing. In addition, this study describes medium-term outcomes. Longer-term follow-up is planned to determine the ongoing prognostic implications of these novel subtypes.

## Conclusions

Machine learning approaches using complex multi-omics data in DCM robustly and reproducibly improved disease characterization and patient stratification. Reproducible subtypes of DCM were identified and were associated with distinct characteristics and clinical outcomes, which may reflect different underlying pathologies. In the drive toward personalized medicine, the subtypes identified in this study may facilitate more targeted approaches to an increasingly diverse repertoire of heart failure therapies.

Perspectives **COMPETENCY IN MEDICAL KNOWLEDGE:** Although myocardial fibrosis is associated with adverse outcomes in patients with DCM, other factors further modify risk, and more precise phenotyping could have important therapeutic implications.

**TRANSLATIONAL OUTLOOK:** Adequately powered studies are needed to evaluate these stratified approaches to the increasingly diverse therapeutic array for patients with DCM.

## Funding Support and Author Disclosures

This work was supported by the UK Medical Research Council (UT- MR/M003191/1; DOR-MRC: MC-A658-5QEB0), Elliot's Touch, National Institute for Health Research Royal Brompton Biomedical Research Unit, National Institute for Health Research Imperial College Biomedical Research Centre, British Heart Foundation (SP/10/10/28431; SP/17/11/32885; RE/18/4/34215; DOR: RG/19/6/34387), Fondation Leducq (11 CVD-01, 16 CVD-03), Wellcome Trust (107469/Z/15/Z), Rosetrees Trust, Alexander Jansons Foundation, CORDA, and the Society of Cardiovascular Magnetic Resonance. This research was funded in part by the Wellcome Trust. The funders had no input in the design and conduct of the study; collection, management, analysis, and interpretation of the data; preparation, review, or approval of the manuscript; and decision to submit the manuscript for publication. Dr Hazebroek has received funding from the Kootstra Talented Post-Doc Fellowship. Dr Ware has served as a consultant for MyoKardia and Foresite Labs. Dr Pennell has served as a consultant for Chiesi; has received research support from Bayer and Siemens; and has received speakers fees from Chiesi and Bayer. Dr Cooper has served as a board member for the Myocarditis Foundation; and has served as a consultant for Kiniksa, CardiolRx, Stromal Therapeutics, and Bristol Myers Squibb. Dr Januzzi is a Trustee of the American College of Cardiology; has received research support from Applied Therapeutics, Innolife, Novartis Pharmaceuticals, and Abbott Diagnostics; has received consulting income from Abbott, Janssen, Novartis, and Roche Diagnostics; and has served on Clinical Endpoint Committees/Data Safety Monitoring Boards for Abbott, AbbVie, Amgen, Bayer, CVRx, Janssen, MyoKardia, and Takeda. Dr Cook is co-founder and a shareholder of Enleofen Bio PTE LTD. Dr Deo has received funding from the National Institutes of Health/National Heart, Lung, and Blood Institute (DP2 HL123228), and One Brave Idea. Prof Heymans has received funding from IMI2-CARDIATEAM (N° 821508), the Netherlands Cardiovascular Research Initiative, an initiative with support of the Dutch Heart Foundation, CVON2016-Early HFPEF, 2015-10, CVONShe-PREDICTS, grant 2017-21, CVON Arena-PRIME, and 2017-18; is supported by FWO G091018N and FWO G0B5930N; has received personal fees for scientific advice to AstraZeneca, Cellprothera, and Merck; and has received an unrestricted research grant from Pfizer. All other authors have reported that they have no relationships relevant to the contents of this paper to disclose.
