# Supplementary material for: Precision Phenotyping of Dilated Cardiomyopathy Using Multidimensional Data
Source: J Am Coll Cardiol. 2022 Jun 7;79(22):2219–32. doi: 10.1016/j.jacc.2022.03.375 (PMC9168440; doi:10.1016/j.jacc.2022.03.375)
Supplement: Supplemental Data [file mmc1.docx]

**SUPPLEMENTARY MATERIALS**

**Precision Phenotyping of Dilated Cardiomyopathy Using Multidimensional Data**

Upasana Tayal, PhD1,2, Job A.J. Verdonschot, MD6,10, Mark R. Hazebroek, PhD6, James Howard, PhD^1^, John Gregson, PhD3, Simon Newsome, MSc3, Ankur Gulati, PhD2, Chee Jian Pua, PhD4, Brian P. Halliday, PhD1,2, Amrit S. Lota, PhD1,2, Rachel J. Buchan, MSc1,2, Nicola Whiffin, PhD5,1, Lina Kanapeckaite, MBBS2, Resham Baruah, PhD2, Julian W.E. Jarman, PhD2, Declan P. O’Regan^12^, Paul J.R. Barton, PhD1,2,5, James S. Ware, MRCP1,2,5, Dudley J. Pennell, MD1,2, Bouke P. Adriaans, PhD6, Sebastiaan C.A.M. Bekkers, MD6, Jackie Donovan, FRCPath2, Michael Frenneaux, MD1, Leslie T. Cooper, MD^11^, James L. Januzzi Jr MD^7^, John G.F. Cleland, MD1, Stuart A. Cook, PhD4,5, Rahul C. Deo, MD^9^, Stephane R.B. Heymans, MD^6,8 , and Sanjay K. Prasad, MD^1,2

^Equal contribution

**Affiliations:**

^1^National Heart Lung Institute, Imperial College London, London, United Kingdom

^2^Royal Brompton Hospital (Guy’s and St Thomas’s NHS Foundation Trust), London, United Kingdom

^3^Department of Medical Statistics, London School of Hygiene and Tropical Medicine, London, United Kingdom.

^4^National Heart Centre, Singapore

^5^Medical Research Council London Institute of Medical Sciences, Imperial College London, London, United Kingdom

^6^Cardiovascular Research Institute Maastricht (CARIM), Maastricht University Medical Center, Maastricht, The Netherlands.

^7^Cardiology Division, Massachusetts General Hospital, Baim Institute for Clinical Research, Boston, MA, USA

^8^Centre for Molecular and Vascular Biology, Department of Cardiovascular Sciences, KU Leuven, Belgium.

^9^One Brave Idea and Division of Cardiovascular Medicine, Brigham and Women’s Hospital, Boston, MA, USA

^10^Department of Clinical Genetics, Maastricht University Medical Center, Maastricht, the Netherlands.

^11^Mayo Clinic, Jacksonville, FL, USA

^12^MRC London Institute of Medical Sciences, Imperial College London, Hammersmith Hospital Campus, London, United Kingdom

**Study cohort and phenotyping – derivation cohort genetic analysis**

Variants in the DCM genes titin (*TTN*) (truncating variants - frameshift, nonsense and essential splice site variants), *LMNA*, *MYH7*, *TNNT2*, *VCL*, *TPM1*, *TNNC1*, *RBM20*, *DSP*, *BAG3*, *SCN5A* and *TCAP* were prioritized for analysis. Truncating variants in the titin gene (TTNtv) were curated as previously described(1). Variants in the other genes were selected for inclusion if determined likely pathogenic or pathogenic in Cardioclassifier, a semi-automated variant interpretation tool(2). Genetic variants were grouped into 4 classes: TTNtv, LMNA, other sarcomeric variants, or other DCM variant.

**Study cohort and phenotyping – biomarker analysis**

Up to 276 biomarkers putatively linked to cardiovascular disease were evaluated on 3 multiplex immunoassay panels (Proseek Multiplex CVD II, CVD III, Inflammation; Olink Bioscience, Uppsala, Sweden) using serum stored from patient study enrollment. The assay uses proximity extension assay methodology whereby oligonucleotide labelled antibodies bind to the target protein in a pair-wise manner(3). The oligonucleotide pairs then hybridize and undergo extension by DNA polymerase to create a target protein specific DNA reporter sequence which is amplified and detected by standard real-time polymerase chain reaction (PCR)(3). For this reason, results are presented as normalized protein expression values (NPX) and not absolute quantification.

**Study cohort and phenotyping – validation cohort**

The validation cohort comprised 239 patients with a clinical diagnosis of DCM confirmed by LGE-CMR prospectively enrolled in the Maastricht Cardiomyopathy Registry from the Maastricht University Medical Center (MUMC) between 2009-2016. The cohort underwent the same clinical, imaging, genetic and biomarker phenotyping as the derivation cohort (Supplementary materials). All patients had clinical screening at recruitment to the study, cardiovascular magnetic resonance imaging for assessment of cardiac chamber volumes and function and assessment of fibrosis (1.5T, Ingenia, Philips Medical Systems, Best, The Netherlands), analysis of 276 biomarkers on the same three immunoassay panels (Proseek Multiplex CVD II, CVD III, Inflammation; Olink Bioscience, Uppsala, Sweden) as well as quantification of serum creatinine, and targeted genetic analysis for variants in 47 DCM genes (HiSeq 2000 or NextSeq 500, Illumina, San Diego, California), including all of those analyzed in the UK cohort.

**Data Preprocessing – missing data**

Missing values were imputed with the SVDImpute function within the *imputation* package in R. Imputation using the SVD first fills missing values using the mean of the column, and then iteratively re-imputes values until the values stop changing by more than a specified threshold. At each step, the missing values are recalculated using a linear combination of a specified number *(k)* of the most significant components. The process stops when the algorithm “converges” on a solution. For the derivation cohort, variables with missing data as well as the percentage of values that are missing are:

1. MRI variables: right ventricular systolic and diastolic end-diastolic and end-systolic volume (2.3%), left ventricular mass (1.6%), left atrial volume (3.5%)

2. Clinical variables: heart rate (5.4%), NYHA class (6.1%)

3. Laboratory variables: serum creatinine (12.4%), Galectin-3 (2.8%), troponin-I (0.9%)

**Data Preprocessing – PCA**

After imputation of missing data, redundancy in the CMR and biomarker datasets was reduced by performing principal components analysis (PCA), a form of unsupervised learning. This step has two primary purposes: 1) to make our subsequent clustering more robust by reducing the number of separate variables used as inputs and 2) to facilitate interpretation of the intrinsic structure within the dataset, thereby identifying common patterns of structural and functional variation.

PCA takes linear combinations of the original features to derive a new set of features that are ranked in terms of how much of the variability in the data set they explain. Looking first at the CMR data set, we found that the top 6 principal component vectors collectively explain 97% of the phenotypic variability in the data set. The top principal component (MRI-PC1), which explained 39% of the variance, groups together features in a way that reflected concordant biatrial/biventricular impairment in cardiac structure and function. Specifically, patients with high activity for MRI-PC1 had more dilated left atria, more dilated left and right ventricles, higher indexed left ventricular mass, and poorer left ventricular and right ventricular function, including both stroke volume and ejection fraction (Supplementary Figure 3).

Subsequent principal components further refined this profile. MRI-PC2, which accounted for 22% of the variance, emphasized the distinction between ejection fraction and stroke volume (Supplementary Figure 3, yellow to red color gradient). Specifically, it identified a subset of individuals with high indexed stroke volume (for both RV and LV) despite having diminished LV and RV ejection fraction. These individuals tended to have a larger RV end-diastolic volume index. MRI-PC3 which accounted for 14% of the variance, refined the profile of biventricular dysfunction highlighted in MRI-PC1. It separated individuals with a pattern of LV enlargement, diminished LV function, and relatively preserved RV function (i.e. high MRI-PC3 activity) from those with a pattern of RV enlargement, diminished RV function, but relatively preserved LV function (low MRI-PC3 activity).

We also performed PCA on the serum concentrations of 276 biomarkers. In this case the top 20 principal components collectively explained 65% of the variance, though each subsequent biomarker explained less than 0.8% of the variance. Bio-PC3 prominently featured BNP a well-recognized marker of heart failure. We also looked at cross-correlation of MRI-and Bio-PCA vectors (Supplementary Figure 4) and found the highest direct correlation between MRI-PC1 and Bio-PC3 (ρ = 0.20) and between MRI-PC2 and Bio-PC2 (ρ = 0.20) and the strongest inverse correlation between MRI-PC1 and Bio-PC2 (ρ = -0.25).

**Profile regression mixture models for patient grouping**

Although DCM patients show considerable phenotypic heterogeneity, we nonetheless hypothesized that some phenotypic characteristics would recur in distinct patterns, representing dominant activities of one or more pathological pathways. To identify these recurring groups of features, we used profile regression mixture modeling, a semi-supervised statistical learning method that combines unsupervised learning to find natural substructure in a dataset but also prioritizes patient groupings that are associated with some outcome. In contrast to our prior work(4), we selected a semi-supervised approach to minimize the high variability we previously observed with model-based clustering assignments depending on the choice of input features.

We used the PReMiuM R Package to perform this, which simultaneously learns an “assignment model” that groups patients into clusters, as well as a “disease submodel”, which links clusters to an outcome via a regression model. The assignment model uses a Dirichlet process prior on the mixing distribution, which enables selecting the number of clusters as part of the model-fitting process. PReMiuM uses Markov Chain Monte Carlo sampling to jointly fit the assignment and disease submodels. Each iteration of the sampler results in a different clustering of patients and a clustering algorithm (partitioning around medoids) is used to generate an optimal grouping of patients across the entire range of samples.

We used the inputs described in the Data preprocessing section as inputs and used a categorical response variable (NYHA Class I, II, or III/IV) as an outcome. The requirement of NYHA staging reduced our sample to 400 patients. The model was fit with 40000 burn-in and 40000 sampling iterations. Default hyperparameters were used for the Dirichlet process prior. We explored a range of seeds to assess the robustness of the results. In the majority of cases the optimal number of clusters was 3 - though a minority of cases selected 2 clusters (here typically patients from cluster 2 were grouped with cluster 1).

**Survival analysis**

The primary end-point in the derivation cohort was a composite of cardiovascular mortality, major arrhythmic events and major heart failure events. All patients had follow-up data. The median follow-up time was 4.0 years (interquartile range 2.1 to 5.8 years). Major arrhythmic events comprised hemodynamically stable and unstable sustained ventricular tachycardia, ventricular fibrillation, appropriate implantable cardiac defibrillator (ICD) shock and aborted sudden cardiac death. Major heart failure events comprised heart transplantation, left ventricular assist device implantation and unplanned heart failure hospitalization. End-points were defined according to the 2014 American College of Cardiology/American Heart Association definitions for cardiovascular end-points in clinical trials(5,6). All primary end-point events were adjudicated by an independent committee of 3 senior cardiologists with expertise in electrophysiology, heart failure management or clinical trial adjudication, blinded to imaging and biomarker data. Event-free survival was calculated from the date of study entry to the date of the first event in the composite end-point. Data for all patients who were last known to be alive, or who had died after December 31st 2015, were censored on December 31st 2015.

The primary end-point in the validation cohort was a composite of cardiovascular mortality, life-threatening arrhythmias and major heart failure events. All patients had follow-up data. The median follow-up time was 3.1 years (interquartile range 1.7 to 5.0 years). Life-threatening arrhythmias comprised non-fatal ventricular fibrillation (with or without ICD shock), hemodynamically unstable sustained ventricular tachycardia, and/or sustained ventricular tachycardia with appropriate ICD shock. Major heart failure events comprised heart transplantation, left ventricular assist device implantation and unplanned heart failure hospitalization. Event-free survival was calculated from the date of study entry to the date of the first event in the composite end-point. Data for all patients who were last known to be alive, or who had died after October 1^st^ 2016, were censored on October 1^st^ 2016.

**Control cohort for biomarker comparison**

Control participants were selected as a substudy from the UK Digital Heart Project at Imperial College London. In total, 51 participants had serum analyzed for Olink panels CVDII, CVDIII and Inflammation (n=31 men; median age 47, IQR 36-58yo).

In the UK Digital Heart Project study, 1,258 adult volunteers (680 women, age range 18 to 80 years, mean age 40.6 ± 12.8 years) were recruited prospectively via advertisement for the UK Digital Heart Project at Imperial College London. Participants who had known cardiovascular or metabolic disease were excluded from imaging. Those taking prescription medicines were excluded but simple analgesics, antihistamines, and oral contraceptives were acceptable. Women were excluded if they were pregnant or breastfeeding. All participants underwent 3-dimensional CMR. Standard safety contraindications to CMR were applied including a weight limit of 120 kg. All participants provided written informed consent for participation in the study, which was approved by a research ethics committee. Further details are provided in reference(7).

**Supplementary Results**

Supplementary Figure 1: Heatmap showing clinical characteristics across the clusters. Green bar indicates patients belonging to mild, non-fibrotic phenotypic group 1, orange bar is patients belonging to metabolic, profibrotic phenotypic group 2, and the red bar is patients in biventricular impairment phenotypic group 3. Grey bar indicates patients who could not be assigned a cluster following data pre-processing.


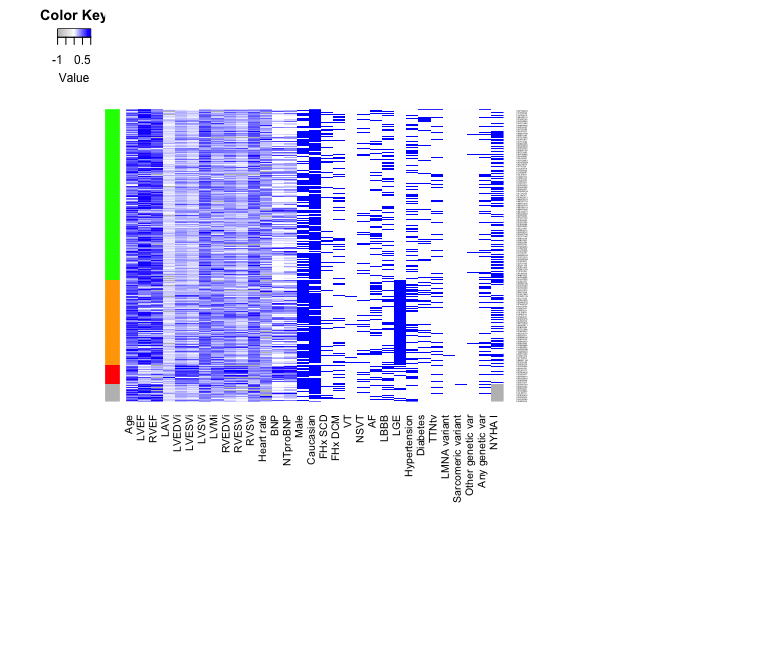


**Supplementary Figure 2: Random survival forests identify novel predictors of prognosis in DCM.** A. Top 15 variables as ranked by variable importance measure (VIMP). B. Partial dependence plot for the soluble IL4-receptor alpha (IL4RA) showing sharp increase in 5-year adverse event risk with individuals with higher levels. Purple triangles represent patients with events while yellow circles represent patients who were event free during follow up. Blue line represents spline fit with standard error shown in dark gray.

**Supplementary Figure 3: Relationship between top 3 MRI principal components**

**Supplementary Figure 4: Cross-correlation of MRI-and Biomarker-PCA vectors**

Supplementary Table 1: Unsupervised clustering to create groupings.

By using NYHA class in our semi supervised approach, we wanted to create clinically relevant groupings instead of groupings that were driven by a less clinically meaningful feature. As a comparator, we performed unsupervised clustering using the same input data and determined that there was a high variability in groupings depending on clustering algorithm chosen. Importantly, none of the unsupervised clustering algorithms yielded clusters with significantly different survival profiles. The p value for the log rank test to compare survival across the clusters is shown.

| Algorithm | Number of clusters | P value (log rank) |
| --- | --- | --- |
| K-means | 3 * | 0.08 |
| BIRCH | 3 * | 0.16 |
| DBSCAN | Did not converge | N/A |
| Spectral clustering | 3 * | 0.74 |
| Gaussian Mixture | 3 * | 0.12 |
| Agglomerative clustering | 3 * | 0.16 |
| OPTICS | 3 | 0.41 |
| Mean shift | Did not converge | N/A |

* Denotes algorithms where the number of clusters is specified

Supplementary Table 2: Table illustrating some of the phenotypic differences between subgroups. +/- indicates increasing or decreasing tendency towards phenotype of interest respectively.

|  | **Subtype 1: Mild, non fibrotic** | **Subtype 2: Profibrotic, metabolic** | **Subtype 3: Biventricular impairment** |
| --- | --- | --- | --- |
| **Demographics** |  |  |  |
| Older age | -/+ | + | + |
| Male sex | + | +++ | +++ |
| Asymptomatic | ++ | ++ | + |
| **Comorbidities** |  |  |  |
| Diabetes mellitus | + | +++ | - |
| Hypertension | + | ++ | ++ |
| **Biomarkers** |  |  |  |
| Renal impairment | - | ++ | ++ |
| Raised Troponin | - | ++ | ++ |
| Raised NT pro BNP | - | ++ | ++ |
| **Arrhythmia** **history** |  |  |  |
| Left bundle branch block | + | + | + |
| Atrial fibrillation | + | + | + |
| Non sustained ventricular tachycardia | + | + | + |
| Sustained ventricular tachycardia | - | ++ | - |
| **Imaging** |  |  |  |
| Myocardial fibrosis | - | ++++ | ++ |
| Severely impaired left ventricular ejection fraction | - | + | ++ |
| Dilated left ventricle | + | ++ | +++ |
| Severely impaired  right ventricular ejection fraction | - | + | ++ |
| Dilated right ventricle | - | - | ++ |
| Dilated left atria | - | - | ++ |
| **Genetics and family history** |  |  |  |
| Family history of DCM or family history of sudden cardiac death | + | + | + |
| Titin truncating variant | + | + | + |
| Other genetic variant | + | + | + |

Supplementary Table 3: Phenotypic groups add prognostic value beyond clinical, proteomic, and imaging markers. A nested series of predictors was evaluated within the Cox proportional-hazards framework and the addition of phenotypic group status was assessed by a log likelihood ratio test. DM= diabetes mellitus, HTN = hypertension, HR = heart rate; LVEDVi = indexed left ventricular end diastolic volume; VT= ventricular tachycardia; NSVT= non sustained ventricular tachycardia; LGE= late gadolinium enhancement; LVEF= left ventricular ejection fraction; RVEF= right ventricular ejection fraction.

| Model | p-value for addition of PG status (LLR test) |
| --- | --- |
| Age, Sex, Race | 1.1x10−6 |
| Age, Sex, Race, LVEDVi, Family history of sudden cardiac death, VT, NSVT, LBBB | 8.8x10−6 |
| Age, Sex, Race, LVEDVi, Family history of sudden cardiac death, VT, NSVT, LBBB, LGE | 0.0088 |
| Age, Sex, Race, LVEDVi, Family history of sudden cardiac death, VT, NSVT, LBBB, LGE, DM, Hypertension, NTproBNP, heart rate, creatinine | 0.0052 |
| Age, Sex, Race, LVEDVi, Family history of sudden cardiac death, VT, NSVT, LBBB, LGE, DM, Hypertension, NTproBNP, heart rate, creatinine, LVEF, NYHA, | 0.00084 |
| Age, Sex, Race, LVEDVi, Family history of sudden cardiac death, VT, NSVT, LBBB, LGE, DM, Hypertension, NTproBNP, heart rate, creatinine, LVEF, NYHA, RVEF | 0.010 |

**Comparison with the MAGGIC score**

We have not sought to create a risk prediction model but our phenotypic subtypes do offer prognostic value beyond traditional variables.

We compared the discrimination of our complete Cox model with the MAGGIC heart failure risk prediction score for the primary composite endpoint. We calculated the MAGGIC score(8) in our cohort using the variables age, sex, BMI, current smoker status, systolic blood pressure, diabetes, NYHA status, LVEF, creatinine, beta blocker and ACE inhibitor use, as well as the interactions between LVEF and age and LVEF and systolic blood pressure . We substituted DCM duration for the heart failure duration variable. COPD was not part of our structured DCM data-collection but a free text search of each participant’s records found no case of COPD. It is possible that COPD was missed and this may affect the performance of the MAGGIC risk score in this cohort. Complete data for all other variables was available for 360 patients.

We present the C-statistics of each model below in Supplementary Table 4. This shows that the Cox proportional hazard models which include the phenotypic subtypes have greater prognostic discrimination for risk compared to the MAGGIC calculator in this cohort of patients with DCM.

**Supplementary Table 4: Comparative C-statistics of a Cox model of the MAGGIC heart failure risk prediction score and the most complete Cox model from Supplementary Table 3**. We also show the results of a simple Cox model using phenotypic subtypes alone.

| **Cox model** | **C statistic** | **Standard error** |
| --- | --- | --- |
| MAGGIC | 0.603 | 0.056 |
| Age, Sex, Race, LVEDVi, Family history of sudden cardiac death, VT, NSVT, LBBB, LGE, DM, Hypertension, NTproBNP, heart rate, creatinine, LVEF, NYHA, RVEF, phenotypic subtypes | 0.759 | 0.049 |
| Phenotypic subtypes alone | 0.688 | 0.044 |

**Supplementary Table 5: Protein biomarkers assayed on the three Olink panels – inflammation, cardiovascular 2 and cardiovascular 3, with corresponding UniProt IDs.**

| **Inflammation panel** | | **CVD III panel** | | | | **CVD II panel** | | | |  |
| --- | --- | --- | --- | --- | --- | --- | --- | --- | --- | --- |
| **Target** | **UniProt No.** | | **Target** | | **UniProt No** | | **Target** | | **UniProt No.** | |
| Adenosine Deaminase (ADA) | P00813 | Tumor necrosis factor receptor superfamily member 14 (TNFRSF14) | | Q92956 | | 2,4-dienoyl-CoA reductase, mitochondrial (DECR1) | | Q16698 | |  |
| Artemin (ARTN) | Q5T4W7 | Low-density lipoprotein receptor (LDL receptor) | | P01130 | | A disintegrin and metalloproteinase with thrombospondin motifs 13 (ADAM-TS13) | | Q76LX8 | |  |
| Axin-1 (AXIN1) | O15169 | Integrin beta-2 (ITGB2) | | P05107 | | ADM (ADM) | | P35318 | |  |
| Beta-nerve growth factor (Beta-NGF) | P01138 | Interleukin-17 receptor A (IL-17RA) | | Q96F46 | | Agouti-related protein (AGRP) | | O00253 | |  |
| Brain-derived neutrophic factor (BDNF) | P23560 | Tumor necrosis factor receptor 2 (TNF-R2) | | P20333 | | Alpha-L-iduronidase (IDUA) | | P35475 | |  |
| Caspase 8 (CASP-8) | Q14790 | Matrix metalloproteinase-9 (MMP-9) | | P14780 | | Angiopoietin-1 (ANG-1) | | Q15389 | |  |
| C-C motif chemokine 4 (CCL4) | P13236 | Ephrin type-B receptor 4 (EPHB4) | | P54760 | | Angiopoietin-1 receptor (TIE2) | | Q02763 | |  |
| C-C motif chemokine 19 (CCL19) | Q99731 | Interleukin-2 receptor subunit alpha (IL2-RA) | | P01589 | | Angiotensin-converting enzyme 2 (ACE2) | | Q9BYF1 | |  |
| C-C motif chemokine 20 (CCL20) | P78556 | Osteoprotegerin (OPG) | | O00300 | | Bone morphogenetic protein 6 (BMP-6) | | P22004 | |  |
| C-C motif chemokine 23 (CCL23) | P55773 | CD166 antigen (ALCAM) | | Q13740 | | Brother of CDO (Protein BOC) | | Q9BWV1 | |  |
| C-C motif chemokine 25 (CCL25) | O15444 | Trefoil factor 3 (TFF3) | | Q07654 | | Carbonic anhydrase 5A, mitochondrial (CA5A) | | P35218 | |  |
| C-C motif chemokine 28 (CCL28) | Q9NRJ3 | P-selectin (SELP) | | P16109 | | Carcinoembryonic antigen-related cell adhesion molecule 8 (CEACAM8) | | P31997 | |  |
| CD40L receptor (CD40) | P25942 | Cystatin-B (CSTB) | | P04080 | | Cathepsin L1 (CTSL1) | | P07711 | |  |
| CUB domain-containing protein 1 (CDCP1) | Q9H5V8 | Monocyte chemotactic protein 1 (MCP-1) | | P13500 | | C-C motif chemokine 17 (CCL17) | | Q92583 | |  |
| C-X-C motif chemokine 1 (CXCL1) | P09341 | Scavenger receptor cysteine-rich type 1 protein M130 (CD163) | | Q86VB7 | | C-C motif chemokine 3 (CCL3) | | P10147 | |  |
| C-X-C motif chemokine 5 (CXCL5) | P42830 | Galectin-3 (Gal-3) | | P17931 | | CD40 ligand (CD40-L) | | P29965 | |  |
| C-X-C motif chemokine 6 (CXCL6) | P80162 | Granulins (GRN) | | P28799 | | Chymotrypsin C (CTRC) | | Q99895 | |  |
| C-X-C motif chemokine 9 (CXCL9) | Q07325 | Matrix extracellular phosphoglycoprotein (MEPE) | | Q9NQ76 | | C-X-C motif chemokine 1 (CXCL1) | | P09341 | |  |
| C-X-C motif chemokine 10 (CXCL10) | P02778 | Bleomycin hydrolase (BLM hydrolase) | | Q13867 | | Decorin (DCN) | | P07585 | |  |
| C-X-C motif chemokine 11 (CXCL11) | O14625 | Perlecan (PLC) | | P98160 | | Dickkopf-related protein 1 (Dkk-1) | | O94907 | |  |
| Cystatin D (CST5) | P28325 | Lymphotoxin-beta receptor (LTBR) | | P36941 | | Fatty acid-binding protein, intestinal (FABP2) | | P12104 | |  |
| Delta and Notch-like epidermal growth factor related receptor (DNER) | Q8NFT8 | Neurogenic locus notch homolog protein 3 (Notch 3) | | Q9UM47 | | Fibroblast growth factor 21 (FGF-21) | | Q9NSA1 | |  |
| Eotaxin-1 (CCL11) | P51671 | Metalloproteinase inhibitor 4 (TIMP4) | | Q99727 | | Fibroblast growth factor 23 (FGF-23) | | Q9GZV9 | |  |
| Eukaryotic translation initiation factor 4E-binding protein 1 (4EBP1) | Q13541 | Contactin-1 (CNTN1) | | Q12860 | | Follistatin (FS) | | P19883 | |  |
| Fibroblast growth factor 5 (FGF-5) | Q8NF90 | Cadherin-5 (CDH5) | | P33151 | | Galectin-9 (Gal-9) | | O00182 | |  |
| Fibroblast growth factor 19 (FGF-19) | O95750 | Trem-like transcript 2 protein (TLT-2) | | Q5T2D2 | | Gastric intrinsic factor (GIF) | | P27352 | |  |
| Fibroblast growth factor 21 (FGF-21) | Q9NSA1 | Fatty acid-binding protein, adipocyte (FABP4) | | P15090 | | Gastrotropin (GT) | | P51161 | |  |
| Fibroblast growth factor 23 (FGF-23) | Q9GZV9 | Tissue factor pathway inhibitor (TFPI) | | P10646 | | Growth hormone (GH) | | P01241 | |  |
| Fms-related tyrosine kinase 3 ligand (FIt3L) | P49771 | Plasminogen activator inhibitor 1 (PAI) | | P05121 | | Growth/differentiation factor 2 (GDF-2) | | Q9UK05 | |  |
| Fractalkine (CX3CL1) | P78423 | C-C motif chemokine 24 (CCL24) | | O00175 | | Heat shock 27 kDa protein (HSP 27) | | P04792 | |  |
| Glial cell line-derived neutrophic factor (hGDNF) | P39905 | Transferrin receptor protein 1 (TR) | | P02786 | | Heme oxygenase 1 (HO-1) | | P09601 | |  |
| Hepatocyte growth factor (HGF) | P14210 | Tumor necrosis factor receptor superfamily member 10C (TNFRSF10C) | | O14798 | | Hydroxyacid oxidase 1 (HAOX1) | | Q9UJM8 | |  |
| Interferon gamma (IFN-gamma) | P01579 | Growth/differentiation factor 15 (GDF-15) | | Q99988 | | Interleukin-1 receptor antagonist protein (IL-1ra) | | P18510 | |  |
| Interleukin-1 alpha (IL-1 alpha) | P01583 | E-selectin (SELE) | | P16581 | | Interleukin-1 receptor-like 2 (IL1RL2) | | Q9HB29 | |  |
| Interleukin-2 (IL-2) | P60568 | Azurocidin (AZU1) | | P20160 | | Interleukin-17D (IL-17D) | | Q8TAD2 | |  |
| Interleukin-2 receptor subunit beta (IL-2RB) | P14784 | Protein delta homolog 1 (DLK-1) | | P80370 | | Interleukin-18 (IL-18) | | Q14116 | |  |
| Interleukin-4 (IL-4) | P05112 | Spondin-1 (SPON1) | | Q9HCB6 | | Interleukin-27 (IL27) | | Q8NEV9 Q14213 | |  |
| Interleukin-5 (IL-5) | P05113 | Myeloperoxidase (MPO) | | P05164 | | Interleukin-4 receptor subunit alpha (IL-4RA) | | P24394 | |  |
| Interleukin-6 (IL-6) | P05231 | C-X-C motif chemokine 16 (CXCL16) | | Q9H2A7 | | Interleukin-6 (IL-6) | | P05231 | |  |
| Interleukin-7 (IL-7) | P13232 | Interleukin-6 receptor subunit alpha (IL-6RA) | | P08887 | | Lactoylglutathione lyase (GLO1) | | Q04760 | |  |
| Interleukin-8 (IL-8) | P10145 | Resistin (RETN) | | Q9HD89 | | Lectin-like oxidized LDL receptor 1 (LOX-1) | | P78380 | |  |
| Interleukin-10 (IL-10) | P22301 | Insulin-like growth factor-binding protein 1 (IGFBP-1) | | P08833 | | Leptin (LEP) | | P41159 | |  |
| Interleukin-10 receptor subunit alpha (IL-10RA) | Q13651 | Chitotriosidase-1 (CHIT1) | | Q13231 | | Lipoprotein lipase (LPL) | | P06858 | |  |
| Interleukin-10 receptor subunit beta (IL10RB) | Q08334 | Tartrate-resistant acid phosphatase type 5 (TR-AP) | | P13686 | | Low affinity immunoglobulin gamma Fc region receptor II-b (IgG Fc receptor II-b) | | P31994 | |  |
| Interleukin-12 subunit beta (IL-12B) | P29460 | C-C motif chemokine 22 (CCL22) | | O00626 | | Lymphotactin (XCL1) | | P47992 | |  |
| Interleukin-13 (IL-13) | P35225 | Pulmonary surfactant-associated protein D (PSP-D) | | P35247 | | Macrophage receptor MARCO (MARCO) | | Q9UEW3 | |  |
| Interleukin-15 receptor subunit alpha (IL-15RA) | Q13261 | Elafin (PI3) | | P19957 | | Matrix metalloproteinase-12 (MMP-12) | | P39900 | |  |
| Interleukin-17A (IL-17A) | Q16552 | Epithelial cell adhesion molecule (Ep-CAM) | | P16422 | | Matrix metalloproteinase-7 (MMP-7) | | P09237 | |  |
| Interleukin-17C (IL-17C) | Q9P0M4 | Aminopeptidase N (AP-N) | | P15144 | | Melusin (ITGB1BP2) | | Q9UKP3 | |  |
| Interleukin-18 (IL-18) | Q14116 | Tyrosine-protein kinase receptor UFO (AXL) | | P30530 | | Natriuretic peptides B (BNP) | | P16860 | |  |
| Interleukin-18 receptor 1 (IL-18R1) | Q13478 | Interleukin-1 receptor type 1 (IL-1RT1) | | P14778 | | NF-kappa-B essential modulator (NEMO) | | Q9Y6K9 | |  |
| Interleukin-20 (IL-20) | Q9NYY1 | Matrix metalloproteinase-2 (MMP-2) | | P08253 | | Osteoclast-associated immunoglobulin-like receptor (hOSCAR) | | Q8IYS5 | |  |
| Interleukin-20 receptor subunit alpha (IL-2oRA) | Q9UHF4 | Tumor necrosis factor receptor superfamily member 6 (FAS) | | P25445 | | Pappalysin-1 (PAPPA) | | Q13219 | |  |
| Interleukin-22 receptor subunit alpha-1 (IL-22RA1) | Q8N6P7 | Myoglobin (MB) | | P02144 | | Pentraxin-related protein PTX3 (PTX3) | | P26022 | |  |
| Interleukin-24 (IL-24) | Q13007 | Tumor necrosis factor ligand superfamily member 13B (TNFSF13B) | | Q9Y275 | | Placenta growth factor (PlGF) | | P49763 | |  |
| Interleukin-33 (IL-33) | O95760 | Myeloblastin (PRTN3) | | P24158 | | Platelet-derived growth factor subunit B (PDGF subunit B) | | P01127 | |  |
| Latency-associated peptide transforming growth factor beta 1 (LAP TGF-beta-1) | P01137 | Proprotein convertase subtilisin/kexin type 9 (PCSK9) | | Q8NBP7 | | Poly [ADP-ribose] polymerase 1 (PARP-1) | | P09874 | |  |
| Leukemia inhibitory factor (LIF) | P15018 | Urokinase plasminogen activator surface receptor (U-PAR) | | Q03405 | | Polymeric immunoglobulin receptor (PIgR) | | P01833 | |  |
| Leukemia inhibitory factor receptor (LIF-R) | P42702 | Osteopontin (OPN) | | P10451 | | Programmed cell death 1 ligand 2 (PD-L2) | | Q9BQ51 | |  |
| Macrophage colony-stimulating factor 1 (CSF-1) | P09603 | Cathepsin D (CTSD) | | P07339 | | Proheparin-binding EGF-like growth factor (HB-EGF) | | Q99075 | |  |
| Macrophage inflammatory protein 1-alpha (MIP-1 alpha) | P10147 | Peptidoglycan recognition protein 1 (PGLYRP1) | | O75594 | | Pro-interleukin-16 (IL16) | | Q14005 | |  |
| Matrix metalloproteinase-1 (MMP-1) | P03956 | Carboxypeptidase A1 (CPA1) | | P15085 | | Prolargin (PRELP) | | P51888 | |  |
| Matrix metalloproteinase-10 (MMP-10) | P09238 | Junctional adhesion molecule A (JAM-A) | | Q9Y624 | | Prostasin (PRSS8) | | Q16651 | |  |
| Monocyte chemotactic protein 1 (MCP-1) | P13500 | Galectin-4 (Gal-4) | | P56470 | | Protein AMBP (AMBP) | | P02760 | |  |
| Monocyte chemotactic protein 2 (MCP-2) | P80075 | Interleukin-1 receptor type 2 (IL-1RT2) | | P27930 | | Proteinase-activated receptor 1 (PAR-1) | | P25116 | |  |
| Monocyte chemotactic protein 3 (MCP-3) | P80098 | Tyrosine-protein phosphatase non-receptor type substrate 1 (SHPS-1) | | P78324 | | Protein-glutamine gamma-glutamyltransferase 2 (TGM2) | | P21980 | |  |
| Monocyte chemotactic protein 4 (MCP-4) | Q99616 | C-C motif chemokine 15 (CCL15) | | Q16663 | | Proto-oncogene tyrosine-protein kinase Src (SRC) | | P12931 | |  |
| Natural killer cell receptor 2B4 (CD244) | Q9BZW8 | Caspase-3 (CASP-3) | | P42574 | | P-selectin glycoprotein ligand 1 (PSGL-1) | | Q14242 | |  |
| Neurotrophin-3 (NT-3) | P20783 | Urokinase-type plasminogen activator (uPA) | | P00749 | | Receptor for advanced glycosylation end products (RAGE) | | Q15109 | |  |
| Neurturin (NRTN) | Q99748 | Carboxypeptidase B (CPB1) | | P15086 | | Renin (REN) | | P00797 | |  |
| Oncostatin-M (OSM) | P13725 | Chitinase-3-like protein 1 (CHI3L1) | | P36222 | | Serine protease 27 (PRSS27) | | Q9BQR3 | |  |
| Osteoprotegerin (OPG) | O00300 | ST2 protein (ST2) | | Q01638 | | Serine/threonine-protein kinase 4 (STK4) | | Q13043 | |  |
| Programmed cell death 1 ligand 1 (PD-L1) | Q9NZQ7 | Tissue-type plasminogen activator (t-PA) | | P00750 | | Serpin A12 (SERPINA12) | | Q8IW75 | |  |
| Protein S100-A12 (EN-RAGE) | P80511 | Secretoglobin family 3A member 2 (SCGB3A2) | | Q96PL1 | | SLAM family member 5 (CD84) | | Q9UIB8 | |  |
| Signaling lymphocytic activation molecule (SLAMF1) | Q13291 | Epidermal growth factor receptor (EGFR) | | P00533 | | SLAM family member 7 (SLAMF7) | | Q9NQ25 | |  |
| SIR2-like protein 2 (SIRT2) | Q8IXJ6 | Insulin-like growth factor-binding protein 7 (IGFBP-7) | | Q16270 | | Sortilin (SORT1) | | Q99523 | |  |
| STAM-binding protein (STAMPB) | O95630 | Complement component C1q receptor (CD93) | | Q9NPY3 | | Spondin-2 (SPON2) | | Q9BUD6 | |  |
| Stem cell factor (SCF) | P21583 | Interleukin-18-binding protein (IL-18BP) | | O95998 | | Stem cell factor (SCF) | | P21583 | |  |
| Sulfotransferase 1A1 (ST1A1) | P50225 | Collagen alpha-1(I) chain (COL1A1) | | P02452 | | Superoxide dismutase [Mn], mitochondrial (SOD2) | | P04179 | |  |
| T-cell surface glycoprotein CD5 (CD5) | P06127 | Paraoxonase (PON 3) (PON3) | | Q15166 | | T-cell immunoglobulin mucin receptor 1 (TIM) | | Q96D42 | |  |
| T-cell surface glycoprotein CD6 isoform (CD6) | Q8WWJ7 | Cathepsin Z (CTSZ) | | Q9UBR2 | | T-cell surface glycoprotein CD4 (CD4) | | P01730 | |  |
| Thymic stromal lymphopoietin (TSLP) | Q969D9 | Matrix metalloproteinase-3 (MMP-3) | | P08254 | | Thrombomodulin (TM) | | P07204 | |  |
| TNF-beta (TNFB) | P01374 | Retinoic acid receptor responder protein 2 (RARRES2) | | Q99969 | | Thrombopoietin (THPO) | | P40225 | |  |
| TNF-related activation cytokine (TRANCE) | O14788 | Intercellular adhesion molecule 2 (ICAM-2) | | P13598 | | Thrombospondin-2 (THBS2) | | P35442 | |  |
| TNF-related apoptosis ligand (TRAIL) | P50591 | Kallikrein-6 (KLK6) | | Q92876 | | Tissue factor (TF) | | P13726 | |  |
| Transforming growth factor alpha (TGF-alpha) | P01135 | Platelet-derived growth factor subunit A (PDGF subunit A) | | P04085 | | TNF-related apoptosis-inducing ligand receptor 2 (TRAIL-R2) | | O14763 | |  |
| Tumor necrosis factor (Ligand) superfamily member 12 (TWEAK) | Q4ACW9 | Tumor necrosis factor receptor 1 (TNF-R1) | | P19438 | | Tumor necrosis factor receptor superfamily member 10A (TNFRSF10A) | | O00220 | |  |
| Tumor necrosis factor (TNF) | P01375 | Insulin-like Growth Factor-Binding Protein 2 (IGFBP-2) | | P18065 | | Tumor necrosis factor receptor superfamily member 11A (TNFRSF11A) | | Q9Y6Q6 | |  |
| Tumor necrosis factor ligand superfamily member 14 (TNFSF14) | O43557 | von Willebrand factor (vWF) | | P04275 | | Tumor necrosis factor receptor superfamily member 13B (TNFRSF13B) | | O14836 | |  |
| Tumor necrosis factor receptor superfamily member 9 (TNFRSF9) | Q07011 | Platelet endothelial cell adhesion molecule (PECAM-1) | | P16284 | | Tyrosine-protein kinase Mer (MERTK) | | Q12866 | |  |
| Urokinase-type plasminogen activator (uPA) | P00749 | N-terminal prohormone brain natriuretic peptide (NT-pro BNP) | | NA | | Vascular endothelial growth factor D (VEGF-D) | | O43915 | |  |
| Vascular endothelial growth factor A (VEGF_A) | P15692 | C-C motif chemokine 16 (CCL16) | | O15467 | | V-set and immunoglobulin domain-containing protein 2 (VSIG2) | | Q96IQ7 | |  |

**Comparing biomarkers across phenotypic groups**

To define the phenotypic groups, we used the top principal components of the biomarkers. However, after establishing the phenotypic groups, we then compared the biomarker profile of the individual 276 proteins across the phenotypic groups in the derivation cohort.

By ANOVA, 60 of the 276 biomarkers differed across the 3 phenotypic groups at a 5% false discovery rate (FDR). The top biomarkers with the greatest statistical difference across groups are shown in Supplementary Table 6. Of these, the top two, hsTroponin-I and NT-proBNP are well known markers of cardiac risk. Whilst all the biomarkers have some previous association with cardiovascular disease (a prerequisite for their inclusion in the biomarker panel), of the remaining significant biomarkers, FGF23, GDF15 and adrenomedullin have been reported to be associated with heart failure severity. IL-4 receptor alpha (IL4RA) also discriminated amongst phenotypic groups.

To investigate the importance of protein biomarkers for prediction of survival, we revisited survival analysis in the derivation cohort using the random survival forests algorithm to generate models with biomarkers and clinical variables as features.

The top predictive feature was serum concentration of interleukin-4 receptor alpha (IL4RA). IL4RA is a transmembrane receptor for interleukins-4 and -13 that is expressed on both innate and adaptive immune cells. It is associated with inflammatory and fibrotic pathways(9). The role of IL-4 in the development of heart disease is complex and it has been shown to have both beneficial and adverse effects in preclinical studies(9). However it has not previously been associated with outcome for heart failure or cardiomyopathy. We evaluated potential alternative explanations for the upregulation of IL4R in this cohort and there was no significant difference in the prevalence of asthma or a history suggestive of myocarditis between groups.

To characterize the dependence of the survival model on individual variables, we plotted partial dependence plots, which look at the survival curves as a function of a variable of interest at a specific time point. For IL4RA, we noted a sharp increase in mortality at higher concentrations (Supplementary Figure 2). IL4RA was strongly associated with outcome in both the derivation (hazard ratio for primary endpoint, HR, 3.6, 95% CI 1.9-6.5, p= 0.00002) and validation cohorts (HR 1.94, 95% CI 1.3-2.8, p= 0.00005). This suggests that IL4RA is a novel prognostic marker for DCM.

**Supplementary Table 6:** **By ANOVA, 60 of the 276 biomarkers differed across the 3 phenotypic groups at a 5% false discovery rate (FDR).** The top biomarkers with the greatest statistical difference across groups are shown below. NPX= normalized protein expression value. A complete list of all the biomarkers across the 3 groups is shown in Supplementary Table 8.

| Variable (unit) | 1 (N = 249) | 2 (N = 124) | 3 (N = 27) | p-value |
| --- | --- | --- | --- | --- |
| Adrenomedullin (NPX) | 6.2 (5.7-6.6) | 6.3 (6-6.8) | 7 (6.5-7.5) | <0.00001 |
| Angiotensin-converting enzyme 2 (NPX) | 3.3 (2.9-3.7) | 3.6 (3.1-4.2) | 4 (3.5-4.5) | <0.00001 |
| BNP (NPX) | 1.2 (0.6 -2.7) | 2 (0.8-3.6) | 4.5 (3.4-5.9) | <0.00001 |
| CCL23 (NPX) | 9.5 (9.2-9.7) | 9.6 (9.3-10) | 9.9 (9.6-10) | 0.000015 |
| FGF23 (NPX) | 2.5 (2.1-3.1) | 2.7 (2.3-3.4) | 3.4 (2.8-5.7) | 0.000048 |
| GDF15 (NPX) | 4.9 (4.4-5.7) | 5.2 (4.5-6.2) | 5.6 (5-6.8) | 0.00011 |
| GDNF (NPX) | 2.1 (1.9-2.4) | 2.2 (2-2.5) | 2.5 (2.2-2.7) | 0.000028 |
| Hs Troponin-I (ng/mL) | 2.8 (1.1-6.6) | 8.9 (4-15) | 14 (7-25) | <0.00001 |
| IGFBP2 (NPX) | 7.5 (6.9-8.1) | 7.6 (7-8.5) | 8.4 (7.5-8.8) | 0.0002 |
| Il4RA (NPX) | 2.6 (2.3-2.9) | 2.7 (2.4-2.9) | 2.9 (2.6-3.4) | 0.0004 |
| Interleukin 6 (NPX) | 3.4 (2.9-4.1) | 3.5 (2.9-4.2) | 4.2 (3.6-5.8) | 0.00026 |
| LIFR (NPX) | 3.4 (3.2-3.5) | 3.5 (3.3-3.6) | 3.7 (3.4-3.9) | <0.00001 |
| Myoglobin (NPX) | 6.7 (6.1-7.4) | 7.1 (6.6-7.9) | 7 (6.4-7.8) | 0.00018 |
| NT-proBNP (NPX) | 1.9 (0.89-3.2) | 2.8 (1.4-3.9) | 4.4 (3.7-5.7) | <0.00001 |
| Renin (NPX) | 7.4 (6.9-8.1) | 7.9 (7.1-8.7) | 8.1 (7.6-9) | 0.00005 |
| TNFRSF13B (NPX) | 7.6 (7.3-7.9) | 7.6 (7.5-8.1) | 7.9 (7.7-8.3) | 0.00026 |
| TRAIL-R2 (NPX) | 4.9 (4.6-5.2) | 5 (4.7-5.3) | 5.4 (5-5.6) | <0.00001 |

Supplementary Table 7: Comparison of biomarker distribution between DCM patients and control subjects. Biomarkers are taken from Olink panels CVDII, CVDIII, and Inflammation. NPX= normalized protein expression values. Data are shown as median (interquartile range) and compared using the Mann Whitney test. Full protein names and corresponding UniProt IDs are shown in Supplementary Table 5.

| **Biomarker (NPX)** | **DCM, n=426** | **Controls, n=51** | **p value** |
| --- | --- | --- | --- |
| ANG.1 | 10.48 [10.25, 10.65] | 10.71 [10.56, 10.86] | <0.001 |
| ADM | 6.29 [5.83, 6.75] | 5.69 [5.25, 5.96] | <0.001 |
| CD40.L | 8.23 [7.56, 8.70] | 8.81 [8.36, 9.34] | <0.001 |
| SLAMF7 | 2.03 [1.69, 2.38] | 2.03 [1.83, 2.33] | 0.865 |
| PlGF | 7.14 [6.91, 7.36] | 7.00 [6.89, 7.19] | 0.054 |
| ADAM.TS13 | 6.08 [5.93, 6.21] | 6.24 [6.14, 6.34] | <0.001 |
| Protein.BOC | 4.73 [4.48, 4.94] | 5.02 [4.81, 5.26] | <0.001 |
| IL.4RA | 2.63 [2.38, 2.93] | 2.55 [2.31, 2.72] | 0.037 |
| SRC | 4.67 [4.11, 5.36] | 5.13 [4.49, 5.46] | 0.026 |
| IL.1ra | 4.53 [4.05, 5.03] | 4.14 [3.87, 4.51] | <0.001 |
| IL.6.x | 2.59 [2.10, 3.35] | 2.74 [2.31, 3.03] | 0.311 |
| TNFRSF10A | 2.97 [2.67, 3.27] | 2.83 [2.67, 3.02] | 0.022 |
| STK4 | 1.52 [1.01, 2.21] | 1.50 [0.96, 1.79] | 0.136 |
| IDUA | 5.08 [4.79, 5.39] | 5.20 [5.04, 5.44] | 0.031 |
| TNFRSF11A | 5.18 [4.89, 5.54] | 5.17 [4.97, 5.39] | 0.502 |
| PAR.1 | 3.62 [3.38, 3.88] | 3.64 [3.50, 3.79] | 0.542 |
| TRAIL.R2 | 4.93 [4.65, 5.26] | 4.67 [4.49, 4.87] | <0.001 |
| PRSS27 | 8.15 [7.85, 8.46] | 8.40 [8.14, 8.66] | <0.001 |
| TIE2 | 7.71 [7.51, 7.86] | 7.83 [7.74, 7.92] | <0.001 |
| TF | 5.31 [5.08, 5.54] | 5.37 [5.20, 5.56] | 0.297 |
| IL1RL2 | 4.34 [4.07, 4.61] | 4.47 [4.10, 4.77] | 0.163 |
| PDGF.subunit.B | 10.60 [10.44, 10.71] | 10.72 [10.59, 10.78] | <0.001 |
| IL27 | 3.59 [3.29, 3.84] | 3.54 [3.37, 3.78] | 0.701 |
| IL.17D | 2.09 [1.85, 2.32] | 2.19 [1.95, 2.33] | 0.345 |
| CXCL1.x | 8.68 [8.30, 9.08] | 8.24 [7.84, 8.48] | <0.001 |
| LOX.1 | 7.98 [7.40, 8.51] | 8.38 [7.93, 8.66] | 0.002 |
| Gal.9 | 6.59 [6.31, 6.85] | 6.31 [6.08, 6.52] | <0.001 |
| GIF | 5.51 [5.01, 6.06] | 5.54 [5.07, 6.04] | 0.849 |
| SCF.x | 9.30 [8.99, 9.62] | 9.30 [9.07, 9.47] | 0.444 |
| IL.18.x | 8.29 [7.92, 8.59] | 7.03 [6.66, 7.34] | <0.001 |
| FGF.21 | 6.31 [5.36, 7.23] | 3.15 [2.46, 4.00] | <0.001 |
| PIgR | 5.95 [5.81, 6.06] | 5.93 [5.84, 6.04] | 0.97 |
| RAGE | 4.88 [4.52, 5.20] | 5.02 [4.81, 5.16] | 0.057 |
| SOD2 | 8.32 [8.17, 8.51] | 8.38 [8.24, 8.50] | 0.31 |
| CTRC | 9.93 [9.52, 10.47] | 10.34 [9.86, 10.70] | 0.003 |
| FGF.23.x | 2.66 [2.21, 3.34] | 2.13 [1.96, 2.34] | <0.001 |
| SPON2 | 8.99 [8.85, 9.12] | 8.91 [8.84, 9.07] | 0.11 |
| GH | 9.12 [7.24, 10.66] | 10.20 [8.68, 11.52] | <0.001 |
| FS | 11.21 [10.83, 11.60] | 11.10 [10.66, 11.70] | 0.443 |
| GLO1 | 6.67 [6.25, 7.16] | 6.83 [6.36, 7.36] | 0.273 |
| CD84 | 6.23 [5.87, 6.51] | 6.57 [6.35, 6.77] | <0.001 |
| PAPPA | 3.04 [2.69, 3.38] | 3.05 [2.70, 3.32] | 0.746 |
| SERPINA12 | 3.05 [2.53, 3.75] | 3.46 [2.51, 4.28] | 0.158 |
| REN | 7.61 [6.95, 8.37] | 6.83 [6.48, 7.04] | <0.001 |
| DECR1 | 2.73 [2.30, 3.20] | 2.51 [2.14, 2.88] | 0.016 |
| MERTK | 4.14 [3.83, 4.38] | 4.07 [3.87, 4.26] | 0.321 |
| TIM | 8.51 [7.89, 9.08] | 7.85 [7.48, 8.37] | <0.001 |
| THBS2 | 5.79 [5.57, 5.98] | 5.75 [5.66, 5.90] | 0.811 |
| TM | 8.29 [8.04, 8.53] | 8.49 [8.26, 8.70] | 0.001 |
| VSIG2 | 3.25 [2.88, 3.69] | 3.17 [2.88, 3.52] | 0.264 |
| AMBP | 6.79 [6.63, 6.95] | 6.83 [6.73, 6.95] | 0.283 |
| PRELP | 6.23 [6.06, 6.38] | 6.26 [6.13, 6.38] | 0.374 |
| HO.1 | 10.57 [10.24, 10.85] | 10.47 [10.12, 10.68] | 0.129 |
| XCL1 | 4.79 [4.44, 5.21] | 4.72 [4.44, 4.97] | 0.231 |
| IL16 | 5.13 [4.76, 5.53] | 4.73 [4.51, 5.10] | <0.001 |
| SORT1 | 6.38 [6.19, 6.58] | 6.53 [6.44, 6.74] | <0.001 |
| CEACAM8 | 4.98 [4.29, 5.77] | 4.92 [4.46, 5.24] | 0.264 |
| PTX3 | 2.40 [2.04, 2.88] | 2.37 [2.00, 2.77] | 0.61 |
| PSGL.1 | 4.33 [4.06, 4.54] | 4.42 [4.30, 4.54] | 0.063 |
| CCL17 | 8.62 [7.98, 9.20] | 8.74 [8.29, 9.43] | 0.172 |
| CCL3 | 2.24 [1.91, 2.63] | 1.81 [1.62, 2.10] | <0.001 |
| MMP.7 | 10.42 [10.08, 10.71] | 10.08 [9.86, 10.41] | <0.001 |
| IgG.Fc.receptor.II.b | 1.71 [1.23, 2.12] | 1.57 [1.19, 2.09] | 0.426 |
| ITGB1BP2 | 0.87 [0.87, 0.89] | 0.87 [0.87, 0.87] | 0.258 |
| DCN | 5.04 [4.85, 5.20] | 5.03 [4.94, 5.12] | 0.822 |
| Dkk.1 | 10.00 [9.72, 10.23] | 10.31 [10.07, 10.48] | <0.001 |
| LPL | 9.53 [9.17, 9.87] | 9.74 [9.25, 10.02] | 0.042 |
| PRSS8 | 8.70 [8.45, 8.98] | 8.46 [8.11, 8.79] | 0.001 |
| AGRP | 2.54 [2.20, 2.88] | 2.36 [2.09, 2.56] | 0.006 |
| HB.EGF | 6.60 [6.26, 6.93] | 7.34 [7.03, 7.76] | <0.001 |
| GDF.2 | 4.46 [4.10, 4.80] | 4.90 [4.68, 5.17] | <0.001 |
| FABP2 | 8.44 [7.78, 9.08] | 8.12 [7.65, 8.73] | 0.105 |
| THPO | 2.82 [2.56, 3.06] | 3.08 [2.85, 3.33] | <0.001 |
| MARCO | 6.06 [5.82, 6.25] | 6.11 [5.98, 6.22] | 0.119 |
| GT | 1.12 [0.72, 1.57] | 1.10 [0.86, 1.34] | 0.864 |
| BNP | 1.54 [0.62, 3.29] | 0.62 [0.62, 0.62] | <0.001 |
| MMP.12 | 7.03 [6.52, 7.62] | 6.78 [6.26, 7.04] | 0.002 |
| ACE2 | 3.39 [3.02, 3.94] | 2.82 [2.62, 3.12] | <0.001 |
| PD.L2 | 2.52 [2.25, 2.80] | 2.67 [2.46, 2.76] | 0.07 |
| CTSL1 | 5.03 [4.71, 5.38] | 4.88 [4.68, 5.05] | 0.019 |
| hOSCAR | 9.70 [9.53, 9.92] | 9.81 [9.66, 9.97] | 0.023 |
| TNFRSF13B | 7.66 [7.40, 8.00] | 7.68 [7.54, 7.86] | 0.912 |
| TGM2 | 5.09 [4.56, 5.65] | 5.62 [5.23, 6.03] | <0.001 |
| LEP | 5.99 [5.08, 6.76] | 5.98 [5.00, 6.49] | 0.796 |
| CA5A | 2.12 [1.60, 2.78] | 1.72 [1.22, 2.24] | <0.001 |
| HSP.27 | 8.83 [8.34, 9.25] | 8.53 [8.13, 8.81] | 0.001 |
| CD4 | 3.91 [3.62, 4.18] | 3.66 [3.48, 3.79] | <0.001 |
| NEMO | 4.96 [4.43, 5.59] | 4.20 [3.98, 4.75] | <0.001 |
| VEGF.D | 7.01 [6.71, 7.33] | 7.06 [6.86, 7.34] | 0.357 |
| PARP.1 | 0.56 [0.53, 0.87] | 0.53 [0.53, 0.66] | 0.01 |
| HAOX1 | 4.43 [3.53, 5.53] | 3.43 [2.68, 4.41] | <0.001 |
| TNFRSF14 | 4.47 [4.19, 4.94] | 4.62 [4.39, 4.93] | 0.066 |
| LDL.receptor | 3.50 [3.01, 4.09] | 3.51 [3.04, 4.14] | 0.916 |
| ITGB2 | 4.45 [4.12, 4.83] | 4.47 [4.28, 4.85] | 0.237 |
| IL.17RA | 3.08 [2.68, 3.56] | 3.32 [3.02, 3.62] | 0.025 |
| TNF.R2 | 4.50 [4.14, 5.10] | 4.44 [4.20, 4.71] | 0.506 |
| MMP.9 | 5.67 [5.02, 6.28] | 6.15 [5.56, 6.63] | 0.003 |
| EPHB4 | 1.66 [1.42, 1.92] | 1.68 [1.50, 1.90] | 0.469 |
| IL2.RA | 3.86 [3.45, 4.46] | 3.93 [3.64, 4.27] | 0.542 |
| OPG.x | 2.99 [2.65, 3.40] | 3.05 [2.80, 3.34] | 0.44 |
| ALCAM | 5.27 [4.97, 5.60] | 5.34 [5.19, 5.67] | 0.02 |
| TFF3 | 5.62 [5.18, 6.30] | 5.47 [5.08, 5.92] | 0.111 |
| SELP | 9.43 [8.93, 9.91] | 9.67 [9.34, 10.23] | 0.007 |
| CSTB | 5.41 [4.93, 6.07] | 4.84 [4.62, 5.36] | <0.001 |
| MCP.1.x | 3.95 [3.58, 4.40] | 4.08 [3.61, 4.33] | 0.802 |
| CD163 | 6.67 [6.24, 7.09] | 6.76 [6.38, 6.97] | 0.617 |
| Gal.3 | 5.55 [5.12, 5.94] | 5.46 [5.33, 5.68] | 0.998 |
| GRN | 6.47 [6.15, 6.84] | 6.49 [6.34, 6.76] | 0.338 |
| MEPE | 2.63 [2.28, 3.15] | 2.73 [2.45, 3.24] | 0.119 |
| BLM.hydrolase | 6.40 [6.05, 6.78] | 6.41 [6.11, 6.85] | 0.686 |
| PLC | 6.32 [6.01, 6.83] | 6.14 [5.94, 6.37] | 0.015 |
| LTBR | 3.08 [2.76, 3.47] | 3.07 [2.91, 3.34] | 0.674 |
| Notch.3 | 3.26 [2.87, 3.72] | 3.20 [3.01, 3.58] | 0.528 |
| TIMP4 | 4.16 [3.76, 4.65] | 4.18 [3.91, 4.62] | 0.419 |
| CNTN1 | 2.55 [2.31, 2.95] | 2.81 [2.68, 3.16] | <0.001 |
| CDH5 | 2.91 [2.62, 3.28] | 3.05 [2.76, 3.46] | 0.098 |
| TLT.2 | 4.81 [4.46, 5.29] | 5.20 [4.86, 5.44] | <0.001 |
| FABP4 | 5.83 [5.14, 6.79] | 5.15 [4.73, 5.64] | <0.001 |
| TFPI | 8.44 [8.12, 8.81] | 8.55 [8.30, 8.80] | 0.129 |
| PAI | 7.94 [7.62, 8.37] | 8.13 [7.90, 8.52] | 0.006 |
| CCL24 | 5.77 [5.14, 6.66] | 5.60 [5.02, 6.50] | 0.543 |
| TR | 3.99 [3.60, 4.49] | 4.17 [3.82, 4.53] | 0.041 |
| TNFRSF10C | 5.50 [5.05, 5.99] | 5.60 [5.34, 6.04] | 0.247 |
| GDF.15 | 5.03 [4.44, 5.84] | 4.47 [4.17, 4.88] | <0.001 |
| SELE | 3.31 [2.84, 3.88] | 3.20 [2.78, 3.58] | 0.38 |
| AZU1 | 5.57 [4.72, 6.58] | 5.71 [5.11, 6.43] | 0.35 |
| DLK.1 | 5.10 [4.54, 5.72] | 5.22 [4.72, 5.61] | 0.457 |
| SPON1 | 1.13 [0.86, 1.48] | 1.14 [0.96, 1.39] | 0.853 |
| MPO | 4.44 [3.96, 5.12] | 4.68 [4.18, 5.02] | 0.448 |
| CXCL16 | 5.20 [4.89, 5.56] | 5.29 [5.07, 5.54] | 0.128 |
| IL.6RA | 11.25 [10.92, 11.72] | 11.51 [11.13, 11.76] | 0.042 |
| RETN | 6.42 [5.94, 7.01] | 6.55 [6.08, 7.00] | 0.689 |
| IGFBP.1 | 3.80 [2.74, 4.77] | 3.80 [3.01, 5.00] | 0.747 |
| CHIT1 | 5.90 [5.10, 6.86] | 5.75 [5.04, 6.28] | 0.284 |
| TR.AP | 4.97 [4.53, 5.39] | 5.03 [4.77, 5.31] | 0.358 |
| CCL22 | 3.46 [2.82, 4.50] | 3.45 [3.11, 4.63] | 0.169 |
| PSP.D | 2.18 [1.70, 2.86] | 2.14 [1.64, 2.46] | 0.168 |
| PI3 | 5.02 [4.55, 5.70] | 4.76 [4.52, 5.34] | 0.117 |
| Ep.CAM | 5.76 [4.98, 6.58] | 6.45 [5.40, 7.35] | 0.004 |
| AP.N | 4.97 [4.65, 5.34] | 4.96 [4.80, 5.13] | 0.93 |
| AXL | 7.22 [6.88, 7.54] | 7.20 [7.06, 7.54] | 0.233 |
| IL.1RT1 | 6.13 [5.85, 6.52] | 6.23 [6.06, 6.41] | 0.081 |
| MMP.2 | 4.10 [3.79, 4.60] | 4.23 [4.08, 4.69] | 0.01 |
| FAS | 4.57 [4.27, 4.93] | 4.55 [4.23, 4.81] | 0.46 |
| MB | 6.87 [6.27, 7.51] | 6.42 [6.14, 6.91] | 0.003 |
| TNFSF13B | 6.11 [5.85, 6.57] | 6.15 [5.96, 6.46] | 0.795 |
| PRTN3 | 6.34 [5.77, 7.28] | 6.50 [5.85, 7.16] | 0.737 |
| PCSK9 | 1.45 [1.18, 1.73] | 1.44 [1.18, 1.77] | 0.851 |
| U.PAR | 5.22 [4.77, 5.72] | 5.22 [4.89, 5.73] | 0.549 |
| OPN | 4.25 [3.83, 4.93] | 3.89 [3.67, 4.26] | 0.002 |
| CTSD | 5.36 [4.98, 5.83] | 5.23 [5.01, 5.47] | 0.175 |
| PGLYRP1 | 7.52 [7.07, 8.11] | 7.85 [7.34, 8.37] | 0.016 |
| CPA1 | 5.56 [4.90, 6.30] | 5.59 [5.20, 6.08] | 0.6 |
| JAM.A | 4.71 [4.40, 5.17] | 4.84 [4.62, 5.10] | 0.172 |
| Gal.4 | 3.18 [2.73, 3.76] | 3.02 [2.58, 3.17] | 0.005 |
| IL.1RT2 | 4.92 [4.61, 5.28] | 4.94 [4.76, 5.29] | 0.53 |
| SHPS.1 | 3.38 [3.00, 3.84] | 3.41 [2.96, 3.77] | 0.976 |
| CCL15 | 6.50 [6.13, 7.22] | 6.21 [6.04, 6.89] | 0.152 |
| CASP.3 | 6.30 [5.79, 6.97] | 6.24 [5.97, 6.65] | 0.905 |
| uPA | 4.76 [4.45, 5.15] | 4.99 [4.74, 5.22] | 0.013 |
| CPB1 | 4.52 [3.95, 5.28] | 4.59 [4.24, 5.00] | 0.709 |
| CHI3L1 | 7.36 [6.59, 8.09] | 6.87 [6.35, 7.64] | 0.015 |
| ST2 | 3.80 [3.39, 4.45] | 3.70 [3.36, 4.08] | 0.193 |
| t.PA | 6.06 [5.52, 6.60] | 5.24 [4.87, 5.92] | <0.001 |
| SCGB3A2 | 2.53 [2.07, 3.19] | 2.84 [2.26, 3.46] | 0.122 |
| EGFR | 2.89 [2.66, 3.16] | 3.04 [2.92, 3.24] | <0.001 |
| IGFBP.7 | 4.17 [3.83, 4.72] | 4.02 [3.81, 4.32] | 0.093 |
| CD93 | 9.10 [8.76, 9.47] | 9.20 [9.00, 9.41] | 0.075 |
| IL.18BP | 5.28 [4.95, 5.72] | 5.20 [5.04, 5.54] | 0.92 |
| COL1A1 | 3.53 [3.21, 3.98] | 3.80 [3.47, 4.08] | 0.004 |
| PON3 | 5.74 [5.19, 6.35] | 6.37 [5.83, 6.84] | <0.001 |
| CTSZ | 5.20 [4.83, 5.68] | 5.10 [4.93, 5.42] | 0.537 |
| MMP.3 | 6.65 [6.05, 7.33] | 6.76 [6.32, 7.22] | 0.447 |
| RARRES2 | 11.04 [10.78, 11.36] | 11.02 [10.86, 11.16] | 0.411 |
| ICAM.2 | 4.92 [4.58, 5.38] | 4.98 [4.77, 5.29] | 0.233 |
| KLK6 | 6.19 [5.88, 6.63] | 6.53 [6.23, 6.94] | <0.001 |
| PDGF.subunit.A | 6.11 [5.73, 6.54] | 6.51 [6.14, 6.95] | <0.001 |
| TNF.R1 | 6.20 [5.88, 6.68] | 6.15 [5.93, 6.62] | 0.827 |
| IGFBP.2 | 7.56 [6.94, 8.33] | 7.55 [6.85, 8.14] | 0.978 |
| vWF | 7.50 [6.89, 8.21] | 7.09 [6.70, 7.53] | 0.004 |
| PECAM.1 | 4.62 [4.37, 4.98] | 4.72 [4.54, 5.08] | 0.04 |
| NT.pro.BNP | 2.24 [1.11, 3.67] | 0.81 [0.81, 0.98] | <0.001 |
| CCL16 | 6.54 [6.06, 7.14] | 6.27 [5.53, 6.54] | <0.001 |
| IL.8 | 7.50 [7.12, 7.95] | 7.25 [6.90, 7.64] | 0.009 |
| VEGF.A | 10.49 [10.14, 10.93] | 10.55 [10.13, 10.96] | 0.972 |
| BDNF | 6.13 [2.08, 8.61] | 6.71 [3.96, 8.84] | 0.362 |
| MCP.3 | 2.51 [2.16, 2.83] | 2.13 [1.87, 2.41] | <0.001 |
| GDNF | 2.17 [1.94, 2.43] | 2.08 [1.88, 2.36] | 0.087 |
| CDCP1 | 3.33 [2.87, 3.76] | 2.83 [2.54, 3.36] | <0.001 |
| CD244 | 6.17 [5.94, 6.38] | 6.34 [6.14, 6.42] | 0.003 |
| IL.7 | 5.23 [4.88, 5.53] | 5.09 [4.73, 5.36] | 0.016 |
| OPG.y | 10.09 [9.85, 10.36] | 9.97 [9.77, 10.23] | 0.043 |
| LAP.TGF.beta.1 | 8.17 [7.84, 8.49] | 8.15 [7.97, 8.45] | 0.635 |
| IL.6.y | 3.51 [2.90, 4.19] | 1.96 [1.64, 2.34] | <0.001 |
| IL.17C | 1.51 [1.51, 1.81] | 1.51 [1.51, 1.69] | 0.181 |
| MCP.1.y | 10.05 [9.71, 10.41] | 9.98 [9.57, 10.34] | 0.233 |
| IL.17A | 0.62 [0.40, 0.88] | 0.56 [0.42, 0.74] | 0.467 |
| CXCL11 | 7.00 [6.61, 7.62] | 7.20 [6.74, 7.64] | 0.195 |
| AXIN1 | 1.55 [1.19, 1.99] | 1.40 [1.13, 1.66] | 0.052 |
| TRAIL | 8.22 [8.04, 8.40] | 8.21 [8.03, 8.36] | 0.922 |
| IL.20RA | 0.81 [0.81, 0.81] | 0.81 [0.81, 0.81] | 0.573 |
| CXCL9 | 7.78 [7.28, 8.46] | 7.21 [6.97, 7.61] | <0.001 |
| CST5 | 6.96 [6.60, 7.36] | 6.90 [6.36, 7.31] | 0.183 |
| IL.2RB | 0.77 [0.77, 0.77] | 0.77 [0.77, 0.77] | 0.001 |
| IL.1.alpha | -0.19 [-0.19, -0.19] | -0.19 [-0.19, -0.19] | 0.002 |
| OSM | 3.68 [3.08, 4.30] | 4.05 [3.65, 4.58] | 0.007 |
| IL.2 | 1.10 [1.10, 1.10] | 1.10 [1.10, 1.10] | 0.393 |
| CXCL1.y | 8.11 [7.72, 8.49] | 8.77 [8.58, 9.07] | <0.001 |
| TSLP | 0.95 [0.95, 0.95] | 0.95 [0.95, 0.95] | 0.158 |
| CCL4 | 6.62 [6.22, 7.03] | 6.34 [6.03, 6.85] | 0.035 |
| CD6 | 3.52 [3.24, 3.78] | 3.53 [3.29, 3.86] | 0.467 |
| SCF.y | 9.14 [8.82, 9.41] | 9.57 [9.27, 9.75] | <0.001 |
| IL.18.y | 7.19 [6.84, 7.52] | 8.05 [7.85, 8.53] | <0.001 |
| SLAMF1 | 3.37 [3.10, 3.68] | 3.24 [3.05, 3.50] | 0.079 |
| TGF.alpha | 2.92 [2.31, 3.51] | 3.56 [3.09, 3.92] | <0.001 |
| MCP.4 | 3.77 [3.31, 4.14] | 3.75 [3.48, 4.30] | 0.214 |
| CCL11 | 8.28 [7.99, 8.57] | 8.26 [7.90, 8.59] | 0.639 |
| TNFSF14 | 4.26 [3.70, 4.83] | 4.58 [4.10, 5.00] | 0.005 |
| FGF.23.y | 2.60 [2.18, 3.33] | 2.24 [2.09, 2.44] | <0.001 |
| IL.10RA | 1.21 [1.21, 1.22] | 1.21 [1.21, 1.29] | 0.281 |
| FGF.5 | 1.80 [1.59, 1.97] | 1.81 [1.65, 1.97] | 0.723 |
| MMP.1 | 8.08 [7.34, 8.75] | 7.54 [6.98, 8.41] | 0.019 |
| LIF.R | 3.41 [3.23, 3.61] | 3.34 [3.22, 3.44] | 0.03 |
| CCL19 | 9.27 [8.74, 9.82] | 8.84 [8.21, 9.23] | <0.001 |
| IL.15RA | 1.26 [1.06, 1.44] | 1.11 [1.05, 1.22] | 0.004 |
| IL.10RB | 7.38 [7.14, 7.61] | 7.35 [7.12, 7.62] | 0.811 |
| IL.22.RA1 | 2.11 [2.11, 2.11] | 2.11 [2.11, 2.11] | NaN |
| IL.18R1 | 7.74 [7.40, 8.07] | 7.50 [7.27, 7.82] | 0.002 |
| PD.L1 | 4.64 [4.44, 4.89] | 4.50 [4.35, 4.70] | 0.007 |
| Beta.NGF | 1.99 [1.80, 2.25] | 1.88 [1.76, 2.06] | 0.008 |
| CXCL5 | 12.18 [11.56, 12.81] | 12.33 [12.00, 12.78] | 0.144 |
| TRANCE | 4.73 [4.30, 5.16] | 4.89 [4.32, 5.26] | 0.254 |
| HGF | 7.95 [7.64, 8.29] | 7.84 [7.60, 8.07] | 0.096 |
| IL.12B | 4.59 [4.15, 5.13] | 4.50 [4.13, 4.86] | 0.332 |
| IL.24 | 1.21 [1.21, 1.21] | 1.21 [1.21, 1.21] | 0.167 |
| IL.13 | 1.20 [1.20, 1.20] | 1.20 [1.20, 1.20] | 0.694 |
| ARTN | -0.35 [-0.35, -0.35] | -0.35 [-0.35, -0.35] | 0.16 |
| MMP.10 | 9.05 [8.66, 9.46] | 9.14 [8.61, 9.57] | 0.552 |
| IL.10 | 4.10 [3.82, 4.42] | 3.84 [3.71, 4.03] | <0.001 |
| TNF | 0.94 [0.94, 0.94] | 0.94 [0.94, 0.94] | 0.237 |
| CCL23 | 9.57 [9.28, 9.87] | 9.26 [9.09, 9.44] | <0.001 |
| CD5 | 3.77 [3.56, 4.02] | 3.77 [3.64, 4.06] | 0.313 |
| MIP.1.alpha | 2.73 [2.36, 3.16] | 2.24 [1.96, 2.48] | <0.001 |
| Flt3L | 9.04 [8.73, 9.27] | 8.97 [8.68, 9.25] | 0.594 |
| CXCL6 | 8.71 [8.22, 9.16] | 8.84 [8.47, 9.27] | 0.068 |
| CXCL10 | 8.84 [8.46, 9.55] | 8.86 [8.50, 9.23] | 0.44 |
| 4E.BP1 | 7.47 [6.81, 8.41] | 7.18 [6.12, 7.83] | 0.015 |
| IL.20 | 0.76 [0.76, 0.76] | 0.76 [0.76, 0.78] | 0.249 |
| SIRT2 | 3.75 [3.36, 4.42] | 3.86 [3.36, 4.14] | 0.859 |
| CCL28 | 0.92 [0.74, 1.13] | 1.07 [0.84, 1.31] | 0.008 |
| DNER | 7.20 [7.01, 7.37] | 7.43 [7.28, 7.54] | <0.001 |
| EN.RAGE | 5.51 [4.86, 6.29] | 4.44 [3.95, 5.18] | <0.001 |
| CD40 | 9.53 [9.24, 9.77] | 9.32 [9.14, 9.54] | 0.001 |
| IL.33 | 1.68 [1.68, 1.68] | 1.68 [1.68, 1.68] | 0.495 |
| IFN.gamma | 1.14 [1.14, 1.14] | 1.14 [1.14, 1.14] | 0.495 |
| FGF.19 | 7.82 [7.16, 8.50] | 8.13 [7.58, 8.67] | 0.029 |
| IL.4 | 1.20 [1.20, 1.20] | 1.20 [1.20, 1.24] | 0.23 |
| LIF | 0.88 [0.88, 0.88] | 0.88 [0.88, 0.88] | 0.254 |
| NRTN | 1.36 [1.36, 1.36] | 1.36 [1.36, 1.36] | 0.867 |
| MCP.2 | 10.08 [9.68, 10.42] | 10.15 [9.70, 10.59] | 0.296 |
| CASP.8 | 1.95 [1.62, 2.46] | 1.49 [1.29, 1.88] | <0.001 |
| CCL25 | 6.76 [6.33, 7.17] | 6.51 [6.05, 6.94] | 0.009 |
| CX3CL1 | 6.39 [6.14, 6.65] | 6.40 [6.25, 6.64] | 0.54 |
| TNFRSF9 | 6.53 [6.21, 6.84] | 6.32 [6.12, 6.59] | 0.001 |
| NT.3 | 1.72 [1.48, 1.97] | 1.68 [1.47, 1.87] | 0.268 |
| TWEAK | 8.69 [8.48, 8.89] | 8.88 [8.73, 9.14] | <0.001 |
| CCL20 | 5.75 [5.08, 6.43] | 5.22 [4.82, 5.67] | <0.001 |
| ST1A1 | 2.13 [1.48, 2.97] | 2.26 [1.66, 2.80] | 0.701 |
| STAMPB | 3.14 [2.86, 3.58] | 3.20 [2.84, 3.39] | 0.552 |
| IL.5 | 1.64 [1.64, 1.83] | 1.64 [1.64, 2.03] | 0.333 |
| ADA | 4.74 [4.53, 5.03] | 4.75 [4.56, 4.97] | 0.835 |
| TNFB | 3.62 [3.33, 3.84] | 3.74 [3.52, 3.96] | 0.013 |
| CSF.1 | 8.03 [7.86, 8.20] | 7.90 [7.80, 8.07] | 0.011 |

**Supplementary Table 8:** **Distribution of the 276 biomarkers across the 3 phenotypic groups.** Data are shown as median NPX and 25^th^ and 75^th^ centiles (ie interquartile range). NPX= normalized protein expression value. P values are shown from ANOVA comparison testing. Full protein names and corresponding UniProt IDs are shown in Supplementary Table 5.

|  | Group 1 | | | Group 2 | | | Group 3 | | |  |
| --- | --- | --- | --- | --- | --- | --- | --- | --- | --- | --- |
|  | Median | 25th | 75th | Median | 25th | 75th | Median | 25th | 75th | p-value |
| ACE2 | 3.3 | 2.9 | 3.7 | 3.6 | 3.1 | 4.2 | 4 | 3.5 | 4.5 | 9.10E-08 |
| ADA | 4.7 | 4.5 | 5 | 4.8 | 4.5 | 5.1 | 4.8 | 4.6 | 5.1 | 0.74 |
| ADAM.TS13 | 6.1 | 5.9 | 6.2 | 6.1 | 5.9 | 6.2 | 6 | 5.9 | 6.1 | 0.079 |
| ADM | 6.2 | 5.7 | 6.6 | 6.3 | 6 | 6.8 | 7 | 6.5 | 7.5 | 8.30E-10 |
| AGRP | 2.5 | 2.2 | 2.8 | 2.6 | 2.3 | 2.9 | 2.8 | 2.2 | 3.3 | 0.00047 |
| ALCAM | 5.3 | 5 | 5.6 | 5.2 | 4.9 | 5.6 | 5.3 | 5 | 5.8 | 0.91 |
| AMBP | 6.8 | 6.6 | 6.9 | 6.8 | 6.6 | 7 | 6.7 | 6.6 | 6.9 | 0.22 |
| ANG.1 | 10 | 10 | 11 | 10 | 10 | 11 | 10 | 10 | 11 | 0.061 |
| AP.N | 5 | 4.7 | 5.3 | 4.9 | 4.6 | 5.4 | 5.3 | 4.8 | 5.8 | 0.29 |
| ARTN | -0.35 | -0.35 | -0.35 | -0.35 | -0.35 | -0.35 | -0.35 | -0.35 | -0.3 | 0.061 |
| AXIN1 | 1.5 | 1.1 | 2 | 1.6 | 1.2 | 1.9 | 1.8 | 1.4 | 2.3 | 0.31 |
| AXL | 7.2 | 6.9 | 7.5 | 7.2 | 6.8 | 7.7 | 7.3 | 6.9 | 7.8 | 0.95 |
| AZU1 | 5.6 | 4.8 | 6.5 | 5.7 | 4.9 | 6.7 | 5.3 | 4.6 | 7.1 | 0.9 |
| BDNF | 6.3 | 2.2 | 8.6 | 6.1 | 2.7 | 8.6 | 6.4 | -0.04 | 9.1 | 0.99 |
| Beta.NGF | 2 | 1.8 | 2.2 | 2 | 1.7 | 2.3 | 2.2 | 2.1 | 2.5 | 0.0028 |
| BLM.hydrolase | 6.4 | 6 | 6.7 | 6.4 | 6.1 | 6.8 | 6.5 | 6.1 | 7 | 0.65 |
| BMP.6 | 4.4 | 3.8 | 4.8 | 4.5 | 4 | 4.9 | 4.8 | 4.1 | 5.4 | 0.0068 |
| BNP | 1.2 | 0.62 | 2.7 | 2 | 0.8 | 3.6 | 4.5 | 3.4 | 5.9 | 4.70E-14 |
| CA5A | 2 | 1.6 | 2.6 | 2.2 | 1.7 | 3 | 2.2 | 1.6 | 2.9 | 0.064 |
| CASP.3 | 6.3 | 5.8 | 7 | 6.1 | 5.7 | 6.8 | 6.8 | 5.6 | 7.4 | 0.5 |
| CASP.8 | 1.9 | 1.6 | 2.4 | 2 | 1.6 | 2.6 | 2.2 | 1.8 | 3 | 0.03 |
| CCL11 | 8.3 | 8 | 8.5 | 8.3 | 8.1 | 8.6 | 8.2 | 7.9 | 8.5 | 0.043 |
| CCL15 | 6.5 | 6.1 | 7.1 | 6.5 | 6.2 | 7.3 | 7 | 6.4 | 7.8 | 0.0034 |
| CCL16 | 6.5 | 6 | 7 | 6.7 | 6.1 | 7.1 | 6.6 | 6.3 | 7.7 | 0.033 |
| CCL17 | 8.6 | 8 | 9.2 | 8.6 | 8 | 9.1 | 9.2 | 8.1 | 9.6 | 0.11 |
| CCL19 | 9.2 | 8.6 | 9.8 | 9.3 | 8.8 | 9.7 | 9.4 | 9.1 | 10 | 0.032 |
| CCL20 | 5.6 | 5 | 6.3 | 5.8 | 5.1 | 6.4 | 6.1 | 5.4 | 7.2 | 0.084 |
| CCL22 | 3.5 | 2.9 | 4.5 | 3.3 | 2.6 | 4.7 | 3.8 | 2.6 | 5.1 | 0.69 |
| CCL23 | 9.5 | 9.2 | 9.7 | 9.6 | 9.3 | 10 | 9.9 | 9.6 | 10 | 0.00000014 |
| CCL24 | 5.7 | 5.1 | 6.6 | 5.9 | 5.3 | 6.9 | 5.8 | 5.1 | 6.2 | 0.32 |
| CCL25 | 6.7 | 6.2 | 7 | 6.9 | 6.4 | 7.3 | 7 | 6.4 | 7.4 | 0.0044 |
| CCL28 | 0.91 | 0.73 | 1.2 | 0.9 | 0.75 | 1.1 | 0.92 | 0.73 | 1 | 0.99 |
| CCL3 | 2.2 | 1.9 | 2.6 | 2.3 | 1.9 | 2.7 | 2.5 | 2.1 | 2.8 | 0.55 |
| CCL4 | 6.6 | 6.2 | 7 | 6.6 | 6.3 | 6.9 | 6.4 | 6.3 | 6.8 | 0.8 |
| CD163 | 6.7 | 6.2 | 7.1 | 6.6 | 6.2 | 7.1 | 6.6 | 6.2 | 7.3 | 0.98 |
| CD244 | 6.2 | 5.9 | 6.3 | 6.1 | 5.9 | 6.3 | 6.2 | 6 | 6.3 | 0.54 |
| CD4 | 3.9 | 3.6 | 4.1 | 3.9 | 3.6 | 4.2 | 4.2 | 3.8 | 4.5 | 0.011 |
| CD40 | 9.5 | 9.2 | 9.7 | 9.5 | 9.3 | 9.8 | 9.8 | 9.4 | 10 | 0.0018 |
| CD40.L | 8.4 | 7.6 | 8.7 | 8.1 | 7.6 | 8.6 | 8.4 | 6.7 | 9 | 0.23 |
| CD5 | 3.8 | 3.6 | 4 | 3.7 | 3.5 | 4 | 4 | 3.5 | 4.2 | 0.35 |
| CD6 | 3.5 | 3.3 | 3.8 | 3.5 | 3.2 | 3.7 | 3.3 | 3 | 3.7 | 0.033 |
| CD84 | 6.2 | 5.9 | 6.5 | 6.2 | 5.9 | 6.4 | 6.3 | 5.6 | 6.5 | 0.56 |
| CD93 | 9.1 | 8.8 | 9.4 | 9.1 | 8.7 | 9.5 | 9.2 | 8.9 | 9.8 | 0.42 |
| CDCP1 | 3.3 | 2.9 | 3.8 | 3.4 | 2.9 | 3.8 | 3.5 | 2.9 | 3.7 | 0.69 |
| CDH5 | 2.9 | 2.6 | 3.2 | 2.9 | 2.7 | 3.3 | 2.8 | 2.6 | 3.4 | 0.32 |
| CEACAM8 | 5 | 4.3 | 5.7 | 4.9 | 4.3 | 5.8 | 5.4 | 4.5 | 6 | 0.42 |
| CHI3L1 | 7.2 | 6.5 | 7.8 | 7.5 | 6.7 | 8.3 | 7.8 | 7.2 | 9.3 | 0.00045 |
| CHIT1 | 5.9 | 5.2 | 6.7 | 5.9 | 5 | 7.1 | 5.4 | 4.6 | 7 | 0.9 |
| CNTN1 | 2.6 | 2.3 | 3 | 2.5 | 2.3 | 2.9 | 2.5 | 2.2 | 2.9 | 0.83 |
| COL1A1 | 3.6 | 3.2 | 4 | 3.5 | 3.2 | 3.9 | 3.6 | 3.3 | 4.3 | 0.73 |
| CPA1 | 5.5 | 4.9 | 6.2 | 5.6 | 4.9 | 6.4 | 5.8 | 4.9 | 6.7 | 0.36 |
| CPB1 | 4.5 | 3.9 | 5.1 | 4.6 | 4 | 5.3 | 4.8 | 4.1 | 5.7 | 0.36 |
| CSF.1 | 8 | 7.8 | 8.2 | 8 | 7.9 | 8.2 | 8.2 | 8.1 | 8.5 | 0.0002 |
| CST5 | 6.9 | 6.5 | 7.3 | 7 | 6.7 | 7.4 | 7.2 | 6.9 | 7.7 | 0.0083 |
| CSTB | 5.4 | 4.9 | 5.9 | 5.4 | 4.9 | 6.3 | 5.8 | 5.1 | 6.5 | 0.07 |
| CTRC | 9.9 | 9.5 | 10 | 10 | 9.5 | 10 | 10 | 9.6 | 10 | 0.8 |
| CTSD | 5.3 | 5 | 5.8 | 5.4 | 5 | 5.8 | 5.4 | 4.9 | 6 | 0.58 |
| CTSL1 | 5 | 4.7 | 5.3 | 5 | 4.7 | 5.3 | 5.6 | 4.8 | 5.8 | 0.0003 |
| CTSZ | 5.2 | 4.8 | 5.7 | 5.2 | 4.8 | 5.6 | 5.2 | 4.8 | 5.8 | 0.71 |
| CX3CL1 | 6.4 | 6.1 | 6.6 | 6.4 | 6.2 | 6.7 | 6.6 | 6.3 | 6.9 | 0.0053 |
| CXCL1.x | 8.8 | 8.3 | 9.1 | 8.6 | 8.3 | 8.9 | 8.8 | 8.4 | 9.3 | 0.15 |
| CXCL1.y | 8.2 | 7.7 | 8.5 | 8 | 7.7 | 8.4 | 8.2 | 7.9 | 8.7 | 0.12 |
| CXCL10 | 8.8 | 8.4 | 9.5 | 8.8 | 8.5 | 9.7 | 9.5 | 8.8 | 9.9 | 0.0078 |
| CXCL11 | 7 | 6.6 | 7.6 | 7 | 6.6 | 7.6 | 7.1 | 6.9 | 8.1 | 0.2 |
| CXCL16 | 5.2 | 4.9 | 5.5 | 5.2 | 4.9 | 5.6 | 5.4 | 4.8 | 5.9 | 0.56 |
| CXCL5 | 12 | 12 | 13 | 12 | 11 | 13 | 12 | 11 | 13 | 0.11 |
| CXCL6 | 8.7 | 8.2 | 9.1 | 8.7 | 8.2 | 9.2 | 8.8 | 8.4 | 9.4 | 0.86 |
| CXCL9 | 7.8 | 7.2 | 8.3 | 7.9 | 7.4 | 8.7 | 7.9 | 7.6 | 8.6 | 0.026 |
| DCN | 5 | 4.8 | 5.2 | 5.1 | 4.9 | 5.2 | 5.1 | 5 | 5.4 | 0.00038 |
| DECR1 | 2.7 | 2.2 | 3.2 | 2.7 | 2.4 | 3.2 | 3 | 2.5 | 3.7 | 0.026 |
| Dkk.1 | 10 | 9.7 | 10 | 10 | 9.7 | 10 | 9.8 | 9.5 | 10 | 0.0097 |
| DLK.1 | 5.1 | 4.5 | 5.6 | 5.1 | 4.6 | 5.8 | 4.8 | 4.2 | 5.7 | 0.23 |
| DNER | 7.2 | 7 | 7.4 | 7.2 | 7 | 7.4 | 7.1 | 6.9 | 7.3 | 0.5 |
| EGFR | 2.9 | 2.7 | 3.2 | 2.9 | 2.7 | 3.2 | 2.8 | 2.4 | 3.1 | 0.55 |
| EN.RAGE | 5.4 | 4.9 | 6.2 | 5.6 | 4.9 | 6.4 | 5.9 | 5 | 6.8 | 0.19 |
| Ep.CAM | 5.7 | 5 | 6.6 | 5.8 | 5 | 6.6 | 6.2 | 5.1 | 6.6 | 0.95 |
| EPHB4 | 1.6 | 1.4 | 1.9 | 1.6 | 1.4 | 1.9 | 1.7 | 1.4 | 2 | 0.18 |
| FABP2 | 8.4 | 7.7 | 9 | 8.5 | 7.8 | 9.1 | 8.6 | 8.1 | 9.2 | 0.39 |
| FABP4 | 5.8 | 5.1 | 6.7 | 5.9 | 5.1 | 6.9 | 6.5 | 5.6 | 7.4 | 0.075 |
| FAS | 4.6 | 4.2 | 4.9 | 4.6 | 4.3 | 5 | 4.6 | 4.3 | 4.9 | 0.62 |
| FGF.19 | 7.7 | 7.2 | 8.4 | 7.9 | 7.3 | 8.5 | 8 | 7.1 | 8.6 | 0.4 |
| FGF.21 | 6.2 | 5.1 | 7 | 6.3 | 5.6 | 7.4 | 7.7 | 6.5 | 8.5 | 0.00055 |
| FGF.21.1 | 4.2 | 3.4 | 5.1 | 4.4 | 3.8 | 5.4 | 5.5 | 4.6 | 6.4 | 0.00029 |
| FGF.23 | 2.5 | 2.1 | 3.1 | 2.7 | 2.3 | 3.4 | 3.4 | 2.8 | 5.7 | 5.50E-12 |
| FGF.5 | 1.8 | 1.6 | 2 | 1.8 | 1.6 | 2 | 1.8 | 1.6 | 1.9 | 0.72 |
| Flt3L | 9 | 8.7 | 9.3 | 9.1 | 8.7 | 9.3 | 9 | 8.7 | 9.3 | 0.82 |
| FS | 11 | 11 | 12 | 11 | 11 | 12 | 11 | 11 | 12 | 0.56 |
| Gal.3 | 5.6 | 5.1 | 5.9 | 5.5 | 5.1 | 5.9 | 5.6 | 5.3 | 6.1 | 0.83 |
| Gal.4 | 3.2 | 2.7 | 3.7 | 3.2 | 2.7 | 3.9 | 3.1 | 2.7 | 4 | 0.16 |
| Gal.9 | 6.5 | 6.3 | 6.8 | 6.6 | 6.3 | 6.9 | 6.8 | 6.4 | 7 | 0.11 |
| GDF.15 | 4.9 | 4.4 | 5.7 | 5.2 | 4.5 | 6.2 | 5.6 | 5 | 6.8 | 0.000006 |
| GDF.2 | 4.5 | 4.1 | 4.8 | 4.4 | 4 | 4.7 | 4.3 | 4 | 4.6 | 0.1 |
| GDNF | 2.1 | 1.9 | 2.4 | 2.2 | 2 | 2.5 | 2.5 | 2.2 | 2.7 | 0.00012 |
| GH | 9.2 | 7.1 | 11 | 9 | 7.3 | 11 | 9.3 | 8.4 | 11 | 0.28 |
| GIF | 5.5 | 5 | 6 | 5.5 | 5 | 6.2 | 5.3 | 5 | 6 | 0.46 |
| GLO1 | 6.6 | 6.2 | 7.1 | 6.7 | 6.3 | 7.1 | 7 | 6.1 | 7.8 | 0.23 |
| GRN | 6.5 | 6.1 | 6.8 | 6.5 | 6.2 | 6.9 | 6.7 | 6.2 | 7 | 0.52 |
| GT | 1.1 | 0.7 | 1.6 | 1.2 | 0.72 | 1.6 | 1.2 | 0.82 | 1.4 | 0.81 |
| HAOX1 | 4.3 | 3.6 | 5.3 | 4.7 | 3.6 | 5.6 | 4.4 | 3.3 | 6 | 0.54 |
| HB.EGF | 6.6 | 6.2 | 6.9 | 6.6 | 6.3 | 6.9 | 6.6 | 6.3 | 6.8 | 0.48 |
| HGF | 7.9 | 7.6 | 8.2 | 8 | 7.7 | 8.3 | 8.5 | 7.8 | 8.8 | 0.00068 |
| HO.1 | 11 | 10 | 11 | 11 | 10 | 11 | 11 | 10 | 11 | 0.22 |
| hOSCAR | 9.7 | 9.5 | 9.9 | 9.6 | 9.5 | 9.9 | 9.7 | 9.6 | 10 | 0.065 |
| HSP.27 | 8.8 | 8.3 | 9.2 | 8.8 | 8.3 | 9.2 | 9.2 | 8.8 | 9.6 | 0.021 |
| ICAM.2 | 4.9 | 4.6 | 5.4 | 4.9 | 4.6 | 5.5 | 5 | 4.6 | 5.7 | 0.68 |
| IDUA | 5.2 | 4.8 | 5.4 | 5 | 4.8 | 5.3 | 5 | 4.7 | 5.2 | 0.18 |
| IFN.gamma | 1.1 | 1.1 | 1.1 | 1.1 | 1.1 | 1.1 | 1.1 | 1.1 | 1.1 | 0.56 |
| IGFBP.1 | 3.6 | 2.6 | 4.7 | 3.8 | 2.8 | 4.7 | 4.7 | 4 | 5.6 | 0.0014 |
| IGFBP.2 | 7.5 | 6.9 | 8.1 | 7.6 | 7 | 8.5 | 8.4 | 7.5 | 8.8 | 0.00012 |
| IGFBP.7 | 4.1 | 3.8 | 4.6 | 4.2 | 3.9 | 4.9 | 4.6 | 4.2 | 5.3 | 0.004 |
| IgG.Fc.receptor.II.b | 1.7 | 1.2 | 2.2 | 1.6 | 1.2 | 2 | 1.9 | 1.1 | 2.1 | 0.44 |
| IL.1.alpha | -0.19 | -0.19 | -0.19 | -0.19 | -0.19 | -0.19 | -0.19 | -0.19 | -0.19 | 0.97 |
| IL.10 | 4.1 | 3.8 | 4.4 | 4.1 | 3.9 | 4.4 | 4.1 | 3.8 | 4.5 | 0.76 |
| IL.10RA | 1.2 | 1.2 | 1.2 | 1.2 | 1.2 | 1.2 | 1.2 | 1.2 | 1.2 | 0.6 |
| IL.10RB | 7.4 | 7.2 | 7.6 | 7.3 | 7.1 | 7.6 | 7.6 | 7.1 | 7.7 | 0.23 |
| IL.12B | 4.6 | 4.2 | 5.1 | 4.6 | 4.1 | 5.1 | 4.6 | 4 | 5.4 | 0.89 |
| IL.13 | 1.2 | 1.2 | 1.2 | 1.2 | 1.2 | 1.2 | 1.2 | 1.2 | 1.2 | 0.22 |
| IL.15RA | 1.2 | 1.1 | 1.4 | 1.3 | 1.1 | 1.5 | 1.3 | 1.2 | 1.5 | 0.075 |
| IL.17A | 0.63 | 0.42 | 0.88 | 0.55 | 0.37 | 0.88 | 0.62 | 0.51 | 0.84 | 0.72 |
| IL.17C | 1.5 | 1.5 | 1.8 | 1.5 | 1.5 | 1.8 | 1.7 | 1.5 | 1.9 | 0.19 |
| IL.17D | 2.1 | 1.8 | 2.3 | 2.1 | 1.8 | 2.4 | 2.1 | 1.8 | 2.4 | 0.45 |
| IL.17RA | 3.1 | 2.7 | 3.5 | 3 | 2.6 | 3.5 | 3.3 | 2.7 | 3.6 | 0.73 |
| IL.18.x | 8.3 | 7.9 | 8.6 | 8.3 | 7.9 | 8.6 | 8.1 | 7.8 | 8.4 | 0.16 |
| IL.18.y | 7.2 | 6.9 | 7.5 | 7.2 | 6.8 | 7.6 | 7.1 | 6.8 | 7.4 | 0.5 |
| IL.18BP | 5.3 | 4.9 | 5.6 | 5.2 | 4.9 | 5.9 | 5.4 | 4.9 | 6.3 | 0.52 |
| IL.18R1 | 7.7 | 7.4 | 8 | 7.7 | 7.4 | 8.1 | 7.8 | 7.5 | 8.3 | 0.35 |
| IL.1ra | 4.5 | 4 | 5 | 4.5 | 4.1 | 5 | 4.4 | 4.1 | 5 | 0.65 |
| IL.1RT1 | 6.1 | 5.8 | 6.4 | 6.1 | 5.9 | 6.6 | 6.2 | 5.8 | 6.5 | 0.35 |
| IL.1RT2 | 4.9 | 4.6 | 5.3 | 5 | 4.6 | 5.3 | 4.9 | 4.5 | 5.4 | 0.81 |
| IL.2 | 1.1 | 1.1 | 1.1 | 1.1 | 1.1 | 1.1 | 1.1 | 1.1 | 1.1 | 0.49 |
| IL.20 | 0.76 | 0.76 | 0.76 | 0.76 | 0.76 | 0.76 | 0.76 | 0.76 | 0.76 | 0.47 |
| IL.20RA | 0.81 | 0.81 | 0.81 | 0.81 | 0.81 | 0.81 | 0.81 | 0.81 | 0.81 | 0.35 |
| IL.22.RA1 | 2.1 | 2.1 | 2.1 | 2.1 | 2.1 | 2.1 | 2.1 | 2.1 | 2.1 | 0.33 |
| IL.24 | 1.2 | 1.2 | 1.2 | 1.2 | 1.2 | 1.2 | 1.2 | 1.2 | 1.2 | 0.72 |
| IL.2RB | 0.77 | 0.77 | 0.77 | 0.77 | 0.77 | 0.77 | 0.77 | 0.77 | 0.77 | 0.54 |
| IL.33 | 1.7 | 1.7 | 1.7 | 1.7 | 1.7 | 1.7 | 1.7 | 1.7 | 1.7 | 0.37 |
| IL.4 | 1.2 | 1.2 | 1.2 | 1.2 | 1.2 | 1.2 | 1.2 | 1.2 | 1.3 | 0.5 |
| IL.4RA | 2.6 | 2.3 | 2.9 | 2.7 | 2.4 | 2.9 | 2.9 | 2.6 | 3.4 | 0.000015 |
| IL.5 | 1.6 | 1.6 | 1.8 | 1.6 | 1.6 | 1.8 | 1.6 | 1.6 | 1.6 | 0.92 |
| IL.6.x | 2.4 | 2.1 | 3.1 | 2.6 | 2.1 | 3.4 | 3.4 | 2.6 | 4.8 | 0.0000016 |
| IL.6.y | 3.4 | 2.9 | 4.1 | 3.5 | 2.9 | 4.2 | 4.2 | 3.6 | 5.8 | 0.00000055 |
| IL.6RA | 11 | 11 | 12 | 11 | 11 | 12 | 11 | 11 | 12 | 0.92 |
| IL.7 | 5.2 | 4.9 | 5.5 | 5.2 | 4.9 | 5.5 | 5.4 | 5 | 5.7 | 0.77 |
| IL.8 | 7.5 | 7.1 | 7.9 | 7.5 | 7.2 | 8 | 8.1 | 7.4 | 8.7 | 0.011 |
| IL16 | 5.1 | 4.7 | 5.5 | 5.1 | 4.8 | 5.6 | 5.3 | 4.8 | 5.9 | 0.21 |
| IL1RL2 | 4.4 | 4.1 | 4.6 | 4.3 | 4.1 | 4.6 | 4.2 | 3.9 | 4.4 | 0.086 |
| IL2.RA | 3.9 | 3.4 | 4.4 | 3.8 | 3.4 | 4.6 | 3.8 | 3.5 | 4.8 | 0.37 |
| IL27 | 3.6 | 3.3 | 3.8 | 3.6 | 3.3 | 3.8 | 3.8 | 3.5 | 4.5 | 0.000019 |
| ITGB1BP2 | 0.87 | 0.87 | 0.87 | 0.87 | 0.87 | 0.87 | 0.87 | 0.87 | 1 | 0.25 |
| ITGB2 | 4.5 | 4.1 | 4.8 | 4.3 | 4.1 | 4.9 | 4.3 | 4 | 4.8 | 0.55 |
| JAM.A | 4.7 | 4.4 | 5.1 | 4.7 | 4.3 | 5.2 | 5 | 4.5 | 5.7 | 0.081 |
| KLK6 | 6.2 | 5.9 | 6.5 | 6.2 | 5.9 | 6.8 | 6.3 | 6.1 | 6.8 | 0.11 |
| LAP.TGF.beta.1 | 8.2 | 7.8 | 8.5 | 8.2 | 7.9 | 8.5 | 8.2 | 7.8 | 8.5 | 0.61 |
| LDL.receptor | 3.5 | 3.1 | 4.1 | 3.5 | 2.9 | 4 | 3 | 2.6 | 3.6 | 0.13 |
| LEP | 6.2 | 5.2 | 6.9 | 5.8 | 4.9 | 6.5 | 5.5 | 4.6 | 6.2 | 0.0017 |
| LIF | 0.88 | 0.88 | 0.88 | 0.88 | 0.88 | 0.88 | 0.88 | 0.88 | 0.88 | 0.68 |
| LIF.R | 3.4 | 3.2 | 3.5 | 3.5 | 3.3 | 3.6 | 3.7 | 3.4 | 3.9 | 3.90E-08 |
| LOX.1 | 8 | 7.4 | 8.5 | 8 | 7.4 | 8.5 | 8.1 | 7.3 | 8.5 | 0.79 |
| LPL | 9.6 | 9.2 | 9.9 | 9.5 | 9.1 | 9.8 | 9.5 | 9.1 | 9.7 | 0.095 |
| LTBR | 3 | 2.7 | 3.4 | 3 | 2.8 | 3.5 | 3.4 | 2.9 | 4 | 0.075 |
| MARCO | 6.1 | 5.8 | 6.2 | 6 | 5.8 | 6.2 | 6 | 5.8 | 6.1 | 0.24 |
| MB | 6.7 | 6.1 | 7.4 | 7.1 | 6.6 | 7.9 | 7 | 6.4 | 7.8 | 0.00017 |
| MCP.1.x | 4 | 3.6 | 4.4 | 4 | 3.5 | 4.4 | 3.8 | 3.5 | 4.4 | 0.58 |
| MCP.1.y | 10 | 9.7 | 10 | 10 | 9.7 | 10 | 9.9 | 9.7 | 10 | 0.32 |
| MCP.2 | 10 | 9.7 | 10 | 10 | 9.8 | 10 | 10 | 9.2 | 10 | 0.29 |
| MCP.3 | 2.5 | 2.2 | 2.8 | 2.5 | 2.2 | 2.9 | 2.2 | 1.8 | 2.7 | 0.44 |
| MCP.4 | 3.8 | 3.3 | 4.2 | 3.7 | 3.3 | 4.1 | 3.6 | 3.3 | 3.9 | 0.23 |
| MEPE | 2.6 | 2.2 | 3.1 | 2.7 | 2.3 | 3.1 | 2.6 | 2.3 | 3.3 | 0.17 |
| MERTK | 4.1 | 3.8 | 4.3 | 4.2 | 3.9 | 4.4 | 4.3 | 4 | 4.5 | 0.024 |
| MIP.1.alpha | 2.7 | 2.3 | 3.1 | 2.7 | 2.4 | 3.2 | 3.1 | 2.8 | 3.6 | 0.3 |
| MMP.1 | 8.1 | 7.4 | 8.7 | 8 | 7.2 | 8.8 | 8.7 | 7.4 | 9.4 | 0.032 |
| MMP.10 | 9.1 | 8.6 | 9.4 | 9 | 8.7 | 9.4 | 9 | 8.7 | 9.5 | 0.95 |
| MMP.12 | 7 | 6.5 | 7.6 | 7.1 | 6.6 | 7.7 | 6.9 | 6.4 | 7.6 | 0.14 |
| MMP.2 | 4.1 | 3.8 | 4.5 | 4.1 | 3.8 | 4.7 | 4.3 | 3.9 | 5.1 | 0.2 |
| MMP.3 | 6.6 | 6 | 7.3 | 6.7 | 6.2 | 7.4 | 6.7 | 6.2 | 7.6 | 0.038 |
| MMP.7 | 10 | 10 | 11 | 10 | 10 | 11 | 11 | 10 | 11 | 0.02 |
| MMP.9 | 5.6 | 5 | 6.2 | 5.8 | 5.1 | 6.3 | 5.7 | 4.6 | 6.6 | 0.57 |
| MPO | 4.4 | 4 | 5.1 | 4.5 | 4 | 5.1 | 4.2 | 3.8 | 5.3 | 0.74 |
| NEMO | 4.9 | 4.4 | 5.5 | 5 | 4.5 | 5.7 | 5.2 | 4.3 | 6.1 | 0.15 |
| Notch.3 | 3.2 | 2.8 | 3.6 | 3.3 | 2.9 | 3.8 | 3.7 | 3.5 | 4.1 | 0.019 |
| NRTN | 1.4 | 1.4 | 1.4 | 1.4 | 1.4 | 1.4 | 1.4 | 1.4 | 1.4 | 0.53 |
| NT.3 | 1.7 | 1.5 | 2 | 1.6 | 1.5 | 1.9 | 2 | 1.6 | 2.5 | 0.059 |
| NT.pro.BNP | 1.9 | 0.89 | 3.2 | 2.8 | 1.4 | 3.9 | 4.4 | 3.7 | 5.7 | 1.30E-13 |
| OPG.x | 3 | 2.6 | 3.4 | 3 | 2.6 | 3.5 | 3.1 | 2.7 | 3.6 | 0.29 |
| OPG.y | 10 | 9.8 | 10 | 10 | 9.8 | 10 | 10 | 9.9 | 11 | 0.0056 |
| OPN | 4.2 | 3.8 | 4.7 | 4.4 | 3.8 | 5.1 | 4.7 | 4.3 | 5.7 | 0.004 |
| OSM | 3.7 | 3.1 | 4.3 | 3.6 | 3.1 | 4.4 | 3.8 | 3.1 | 4.4 | 0.81 |
| PAI | 8 | 7.7 | 8.4 | 7.9 | 7.6 | 8.4 | 7.8 | 7.2 | 8.5 | 0.31 |
| PAPPA | 3 | 2.7 | 3.3 | 3 | 2.7 | 3.4 | 3.1 | 2.9 | 3.7 | 0.32 |
| PAR.1 | 3.6 | 3.4 | 3.8 | 3.6 | 3.4 | 3.8 | 3.7 | 3.5 | 4 | 0.043 |
| PARP.1 | 0.54 | 0.53 | 0.8 | 0.58 | 0.53 | 0.9 | 0.57 | 0.53 | 1.2 | 0.03 |
| PCSK9 | 1.4 | 1.2 | 1.7 | 1.4 | 1.1 | 1.7 | 1.4 | 1.3 | 2.1 | 0.24 |
| PD.L1 | 4.6 | 4.4 | 4.8 | 4.7 | 4.4 | 5 | 4.8 | 4.7 | 5.1 | 0.004 |
| PD.L2 | 2.5 | 2.2 | 2.8 | 2.5 | 2.2 | 2.8 | 2.6 | 2.3 | 2.9 | 0.33 |
| PDGF.subunit.A | 6.2 | 5.8 | 6.5 | 6.1 | 5.6 | 6.4 | 6 | 5.3 | 6.6 | 0.74 |
| PDGF.subunit.B | 11 | 10 | 11 | 11 | 10 | 11 | 11 | 10 | 11 | 0.11 |
| PECAM.1 | 4.6 | 4.3 | 4.9 | 4.6 | 4.4 | 5 | 4.6 | 4.4 | 5.1 | 0.71 |
| PGLYRP1 | 7.5 | 7 | 8 | 7.5 | 7.1 | 8.1 | 7.6 | 7.2 | 8.5 | 0.28 |
| PI3 | 5 | 4.5 | 5.6 | 5 | 4.6 | 5.8 | 5.6 | 4.9 | 6.4 | 0.0027 |
| PIgR | 6 | 5.8 | 6 | 5.9 | 5.8 | 6 | 6 | 5.9 | 6.2 | 0.13 |
| PLC | 6.3 | 6 | 6.8 | 6.3 | 6 | 6.9 | 6.7 | 6.2 | 7 | 0.063 |
| PlGF | 7.1 | 6.9 | 7.3 | 7.2 | 7 | 7.4 | 7.3 | 6.9 | 7.7 | 0.0032 |
| PON3 | 5.8 | 5.3 | 6.4 | 5.7 | 5.1 | 6.3 | 5.4 | 4.8 | 6 | 0.057 |
| PRELP | 6.2 | 6 | 6.4 | 6.2 | 6.1 | 6.4 | 6.4 | 6.1 | 6.7 | 0.00053 |
| Protein.BOC | 4.7 | 4.5 | 4.9 | 4.7 | 4.4 | 4.9 | 4.7 | 4.5 | 5 | 0.25 |
| PRSS27 | 8.2 | 7.9 | 8.4 | 8.1 | 7.8 | 8.4 | 8 | 7.7 | 8.3 | 0.019 |
| PRSS8 | 8.7 | 8.4 | 8.9 | 8.8 | 8.5 | 9.1 | 8.9 | 8.4 | 9 | 0.043 |
| PRTN3 | 6.3 | 5.8 | 7.2 | 6.4 | 5.8 | 7.3 | 6.3 | 5.5 | 7.6 | 0.76 |
| PSGL.1 | 4.3 | 4.1 | 4.6 | 4.3 | 4 | 4.5 | 4.2 | 3.9 | 4.4 | 0.21 |
| PSP.D | 2.1 | 1.6 | 2.8 | 2.3 | 1.9 | 3 | 2.3 | 1.9 | 2.8 | 0.22 |
| PTX3 | 2.3 | 2 | 2.8 | 2.4 | 2 | 2.9 | 2.8 | 2.4 | 3.3 | 0.04 |
| RAGE | 4.9 | 4.5 | 5.1 | 4.8 | 4.5 | 5.2 | 5.3 | 4.9 | 5.6 | 0.0013 |
| RARRES2 | 11 | 11 | 11 | 11 | 11 | 11 | 11 | 11 | 11 | 0.78 |
| REN | 7.4 | 6.9 | 8.1 | 7.9 | 7.1 | 8.7 | 8.1 | 7.6 | 9 | 0.00012 |
| RETN | 6.4 | 6 | 6.9 | 6.4 | 5.9 | 7 | 6.5 | 6.3 | 7.4 | 0.19 |
| SCF.x | 9.3 | 9 | 9.6 | 9.3 | 8.9 | 9.6 | 9.2 | 8.5 | 9.5 | 0.024 |
| SCF.y | 9.2 | 8.9 | 9.4 | 9.1 | 8.8 | 9.4 | 9 | 8.3 | 9.4 | 0.067 |
| SCGB3A2 | 2.4 | 2 | 3.1 | 2.7 | 2.1 | 3.2 | 2.6 | 2.2 | 3.6 | 0.4 |
| SELE | 3.3 | 2.8 | 3.8 | 3.3 | 2.9 | 3.9 | 3.3 | 3 | 4.2 | 0.44 |
| SELP | 9.4 | 9 | 10 | 9.4 | 8.9 | 9.9 | 9.2 | 8.7 | 9.8 | 0.33 |
| SERPINA12 | 3.1 | 2.5 | 3.7 | 3 | 2.5 | 3.8 | 3.2 | 2.3 | 3.8 | 0.81 |
| SHPS.1 | 3.4 | 3 | 3.8 | 3.3 | 2.9 | 3.9 | 3.7 | 3.1 | 4.1 | 0.35 |
| SIRT2 | 3.7 | 3.3 | 4.4 | 3.7 | 3.4 | 4.3 | 4.2 | 3.5 | 5 | 0.069 |
| SLAMF1 | 3.3 | 3.1 | 3.6 | 3.4 | 3.1 | 3.7 | 3.5 | 3.3 | 3.8 | 0.087 |
| SLAMF7 | 2 | 1.7 | 2.4 | 2 | 1.6 | 2.4 | 2.2 | 1.9 | 2.7 | 0.029 |
| SOD2 | 8.3 | 8.1 | 8.5 | 8.3 | 8.2 | 8.5 | 8.3 | 8.1 | 8.5 | 0.84 |
| SORT1 | 6.4 | 6.2 | 6.6 | 6.4 | 6.2 | 6.5 | 6.3 | 6.1 | 6.5 | 0.66 |
| SPON1 | 1.1 | 0.83 | 1.4 | 1.1 | 0.82 | 1.5 | 1.4 | 1.1 | 1.8 | 0.0042 |
| SPON2 | 9 | 8.8 | 9.1 | 9 | 8.9 | 9.1 | 9.1 | 9 | 9.2 | 0.0044 |
| SRC | 4.7 | 4.1 | 5.4 | 4.6 | 4 | 5.2 | 4.9 | 4.3 | 5.6 | 0.5 |
| ST1A1 | 2.2 | 1.5 | 3 | 1.9 | 1.5 | 2.8 | 2.6 | 1.9 | 3 | 0.29 |
| ST2 | 3.7 | 3.3 | 4.3 | 3.8 | 3.4 | 4.5 | 4.6 | 3.8 | 5.6 | 0.000016 |
| STAMPB | 3.1 | 2.8 | 3.6 | 3.2 | 2.9 | 3.4 | 3.6 | 2.9 | 4 | 0.17 |
| STK4 | 1.5 | 1.1 | 2.2 | 1.6 | 0.96 | 2.2 | 1.8 | 0.83 | 2.8 | 0.37 |
| t.PA | 6 | 5.5 | 6.5 | 6.2 | 5.6 | 6.7 | 6.1 | 5.5 | 6.4 | 0.44 |
| TF | 5.3 | 5.1 | 5.5 | 5.3 | 5.1 | 5.6 | 5.4 | 5.1 | 5.7 | 0.049 |
| TFF3 | 5.6 | 5.1 | 6.2 | 5.7 | 5.2 | 6.3 | 6.2 | 5.6 | 7.1 | 0.00034 |
| TFPI | 8.4 | 8.1 | 8.8 | 8.5 | 8.2 | 8.9 | 8.4 | 8.1 | 8.7 | 0.21 |
| TGF.alpha | 2.9 | 2.2 | 3.5 | 2.9 | 2.4 | 3.5 | 3.1 | 2.7 | 3.8 | 0.23 |
| TGM2 | 5 | 4.5 | 5.6 | 5.1 | 4.5 | 5.6 | 5.8 | 5.2 | 6.3 | 0.0045 |
| THBS2 | 5.8 | 5.6 | 6 | 5.8 | 5.6 | 6 | 6 | 5.8 | 6.2 | 0.00073 |
| THPO | 2.8 | 2.6 | 3.1 | 2.8 | 2.6 | 3 | 2.8 | 2.5 | 2.9 | 0.26 |
| TIE2 | 7.7 | 7.5 | 7.8 | 7.7 | 7.5 | 7.9 | 7.8 | 7.6 | 8 | 0.047 |
| TIM | 8.5 | 7.8 | 9 | 8.5 | 8.1 | 9.1 | 9 | 7.8 | 9.6 | 0.051 |
| TIMP4 | 4.1 | 3.7 | 4.6 | 4.1 | 3.7 | 4.6 | 4.6 | 4.2 | 5 | 0.065 |
| TLT.2 | 4.8 | 4.5 | 5.3 | 4.7 | 4.4 | 5.2 | 4.6 | 4.1 | 5.2 | 0.77 |
| TM | 8.3 | 8 | 8.5 | 8.3 | 8 | 8.5 | 8.2 | 7.8 | 8.6 | 0.66 |
| TNF | 0.94 | 0.94 | 0.94 | 0.94 | 0.94 | 0.94 | 0.94 | 0.94 | 0.94 | 0.35 |
| TNF.R1 | 6.2 | 5.8 | 6.6 | 6.2 | 5.9 | 6.9 | 6.6 | 6 | 7.5 | 0.012 |
| TNF.R2 | 4.5 | 4.1 | 5 | 4.5 | 4.1 | 5.3 | 4.8 | 4.1 | 5.7 | 0.15 |
| TNFB | 3.6 | 3.3 | 3.8 | 3.6 | 3.3 | 3.8 | 3.5 | 3.2 | 3.8 | 0.41 |
| TNFRSF10A | 2.9 | 2.6 | 3.2 | 3 | 2.7 | 3.3 | 3.1 | 2.9 | 3.5 | 0.00052 |
| TNFRSF10C | 5.5 | 5 | 5.9 | 5.5 | 5 | 5.9 | 5.6 | 5 | 6.1 | 0.46 |
| TNFRSF11A | 5.2 | 4.9 | 5.5 | 5.2 | 4.9 | 5.5 | 5.2 | 4.9 | 5.9 | 0.12 |
| TNFRSF13B | 7.6 | 7.3 | 7.9 | 7.6 | 7.5 | 8.1 | 7.9 | 7.7 | 8.3 | 0.000052 |
| TNFRSF14 | 4.5 | 4.2 | 4.9 | 4.4 | 4.2 | 5 | 4.5 | 4.2 | 5.4 | 0.39 |
| TNFRSF9 | 6.5 | 6.2 | 6.8 | 6.6 | 6.2 | 6.9 | 6.6 | 6.3 | 6.9 | 0.012 |
| TNFSF13B | 6.1 | 5.8 | 6.5 | 6 | 5.8 | 6.5 | 6.5 | 6 | 7 | 0.21 |
| TNFSF14 | 4.2 | 3.7 | 4.8 | 4.2 | 3.7 | 4.8 | 4.4 | 3.7 | 5.1 | 0.82 |
| TR | 4 | 3.6 | 4.5 | 4 | 3.6 | 4.4 | 4.5 | 3.8 | 5.4 | 0.00037 |
| TR.AP | 4.9 | 4.6 | 5.4 | 5 | 4.5 | 5.3 | 4.6 | 4.3 | 5.2 | 0.15 |
| TRAIL | 8.2 | 8.1 | 8.4 | 8.2 | 8 | 8.4 | 8.1 | 8 | 8.2 | 0.00066 |
| TRAIL.R2 | 4.9 | 4.6 | 5.2 | 5 | 4.7 | 5.3 | 5.4 | 5 | 5.6 | 7.20E-09 |
| TRANCE | 4.8 | 4.4 | 5.2 | 4.7 | 4.2 | 5 | 4.6 | 3.8 | 4.9 | 0.013 |
| TSLP | 0.95 | 0.95 | 0.95 | 0.95 | 0.95 | 0.95 | 0.95 | 0.95 | 0.95 | 0.024 |
| TWEAK | 8.7 | 8.5 | 8.9 | 8.7 | 8.4 | 8.9 | 8.6 | 8.4 | 8.7 | 0.0075 |
| U.PAR | 5.2 | 4.8 | 5.6 | 5.2 | 4.8 | 5.8 | 5.4 | 4.9 | 6.7 | 0.036 |
| uPA | 4.8 | 4.4 | 5.1 | 4.7 | 4.4 | 5.1 | 4.7 | 4.4 | 5.4 | 0.95 |
| uPA.2 | 9.9 | 9.7 | 10 | 9.9 | 9.6 | 10 | 10 | 9.7 | 10 | 0.19 |
| VEGF.A | 10 | 10 | 11 | 10 | 10 | 11 | 11 | 10 | 11 | 0.072 |
| VEGF.D | 7 | 6.7 | 7.3 | 7 | 6.7 | 7.4 | 7.3 | 7.1 | 7.6 | 0.00072 |
| VSIG2 | 3.2 | 2.9 | 3.6 | 3.3 | 2.8 | 3.8 | 3.5 | 3.1 | 3.9 | 0.0052 |
| vWF | 7.4 | 6.8 | 8.2 | 7.6 | 7 | 8.3 | 7.6 | 7.2 | 8.3 | 0.16 |
| X4E.BP1 | 7.3 | 6.8 | 8.2 | 7.7 | 7 | 8.4 | 8.3 | 7.5 | 9.7 | 0.00058 |
| XCL1 | 4.7 | 4.4 | 5.2 | 4.8 | 4.4 | 5.2 | 5 | 4.6 | 5.3 | 0.11 |

**Supplementary Table 9: Principal component analysis loadings (Eigenvectors) across the 5 selected principal components from the biomarker analysis**

Variables are sorted according to the absolute magnitude of their contribution towards the first principal component, following normalization. Green and red shadings indicate positive and negative contributions, respectively. The strength of shading indicates the absolute magnitude of contribution.

|  | PC1 | PC2 | PC3 | PC4 | PC5 |
| --- | --- | --- | --- | --- | --- |
| TNF.R1 | 0.117097 | 0.00847 | 0.011692 | -0.04087 | -0.0084 |
| TNF.R2 | 0.115809 | 0.013767 | 0.017458 | -0.04009 | -0.02576 |
| TNFRSF14 | 0.114161 | 0.017337 | -0.05348 | -0.00564 | -0.01268 |
| LTBR | 0.114158 | 0.036491 | 0.027235 | -0.02188 | -0.01403 |
| IL.18BP | 0.114153 | 0.032181 | 0.020694 | -0.02666 | -0.03461 |
| ALCAM | 0.112057 | 0.058811 | 0.005397 | 0.019018 | 0.005292 |
| EPHB4 | 0.111209 | 0.04905 | 0.010115 | -0.02032 | -0.01028 |
| U.PAR | 0.110915 | 0.000148 | -0.03805 | -0.04331 | 0.029057 |
| IGFBP.7 | 0.110374 | 0.042256 | 0.033267 | -0.01451 | 0.01593 |
| CXCL16 | 0.110269 | 0.057936 | 0.007661 | 0.007876 | 0.012629 |
| PLC | 0.109705 | 0.022031 | 0.042787 | -0.006 | -0.00777 |
| IL.1RT1 | 0.109499 | 0.058394 | 0.009234 | -0.00205 | 0.00327 |
| GRN | 0.108777 | 0.04596 | 0.006773 | 0.002245 | -0.0068 |
| uPA | 0.108723 | 0.056061 | -0.01023 | 0.01558 | 0.010587 |
| SPON1 | 0.10867 | 0.042013 | 0.025206 | 0.000773 | 0.029276 |
| FAS | 0.10848 | 0.046309 | 0.01641 | -0.00478 | 0.002372 |
| OPG.x | 0.10833 | 0.040555 | 0.026746 | -0.00329 | 0.016354 |
| JAM.A | 0.108269 | 0.025326 | -0.06059 | -0.00269 | 0.02803 |
| CTSZ | 0.107828 | 0.040369 | 0.005733 | -0.00927 | -0.03415 |
| AXL | 0.107436 | 0.056734 | 0.003888 | -0.00048 | -0.0103 |
| ICAM.2 | 0.10741 | 0.046547 | -0.00302 | 0.000807 | -0.00934 |
| PECAM.1 | 0.107163 | 0.056336 | -0.02494 | 0.031211 | 0.027372 |
| RARRES2 | 0.106922 | 0.019318 | -0.00329 | 0.016267 | -0.03727 |
| CDH5 | 0.106758 | 0.065323 | 0.001046 | 0.022681 | 0.003593 |
| CSTB | 0.105844 | 0.00962 | 0.006458 | -0.06531 | 0.041618 |
| TNFSF13B | 0.105802 | 0.039616 | 0.000457 | -0.01571 | -0.0027 |
| ITGB2 | 0.105399 | 0.053525 | -0.03053 | 0.017349 | 0.001834 |
| IL2.RA | 0.105366 | 0.024864 | 0.0193 | -0.05033 | -0.03609 |
| Notch.3 | 0.105191 | 0.048509 | 0.040234 | -8.1E-05 | 0.023755 |
| PCSK9 | 0.105001 | 0.057875 | -0.00632 | 0.001178 | 0.012875 |
| Gal.3 | 0.104935 | 0.052608 | -0.00313 | -0.00247 | 0.026379 |
| CD93 | 0.104888 | 0.047664 | 0.027599 | 0.019191 | -0.01257 |
| SHPS.1 | 0.104877 | 0.046272 | 0.024274 | -0.01074 | -0.00018 |
| CD163 | 0.104549 | 0.034447 | 0.005477 | -0.00283 | -0.01145 |
| KLK6 | 0.104426 | 0.059753 | 0.015461 | 0.009418 | -0.00053 |
| CTSD | 0.104285 | 0.030328 | -0.01161 | -0.00565 | 0.001071 |
| RETN | 0.103666 | 0.010158 | -0.06679 | -0.04285 | 0.00616 |
| TLT.2 | 0.103625 | 0.042482 | -0.07669 | -0.00437 | -0.02907 |
| MMP.2 | 0.103315 | 0.062347 | 0.032651 | 0.009249 | 0.034023 |
| PGLYRP1 | 0.10326 | 0.006015 | -0.08228 | -0.03339 | 0.015303 |
| Gal.4 | 0.102728 | 0.027389 | 0.026545 | -0.00564 | -0.00219 |
| AP.N | 0.101984 | 0.050473 | 0.004144 | 0.016388 | 0.025714 |
| OPN | 0.100371 | 0.014013 | 0.056557 | -0.05175 | 0.007454 |
| IL.1RT2 | 0.09933 | 0.074011 | -0.00864 | 0.017005 | 0.030459 |
| MCP.1.x | 0.099201 | 0.036886 | -0.01976 | 0.019906 | -0.04254 |
| CNTN1 | 0.099104 | 0.083221 | -0.00502 | 0.02994 | 0.012813 |
| GDF.15 | 0.098769 | -0.02143 | 0.075312 | -0.0581 | 0.015861 |
| TFF3 | 0.098263 | -0.00024 | 0.037147 | -0.04029 | -0.01932 |
| TR.AP | 0.097313 | 0.069374 | -0.01741 | 0.036186 | -0.00645 |
| TFPI | 0.097214 | 0.069108 | 0.010288 | 0.026444 | -0.00961 |
| TIMP4 | 0.097091 | 0.038135 | 0.025191 | -0.00378 | 0.023908 |
| IL.6RA | 0.097031 | 0.069983 | -0.01411 | 0.018536 | -0.00138 |
| CCL15 | 0.096371 | 0.017117 | 0.032652 | -0.02682 | 0.015262 |
| IL.17RA | 0.09625 | 0.061312 | -0.01166 | 0.016411 | 0.005763 |
| TNFRSF10C | 0.096244 | 0.04097 | -0.03288 | -0.02186 | -0.00824 |
| SELE | 0.095932 | 0.037229 | -0.01173 | 4.2E-05 | 0.019726 |
| EGFR | 0.095891 | 0.100655 | -0.02449 | 0.044937 | 0.007131 |
| MEPE | 0.094155 | 0.059963 | -0.00706 | 0.006752 | -0.01256 |
| PI3 | 0.093951 | 0.024956 | 0.022394 | -0.03252 | 0.004909 |
| PDGF.subunit.A | 0.093905 | 0.030236 | -0.10227 | 0.058633 | -0.05035 |
| SELP | 0.093813 | 0.05111 | -0.07493 | 0.023733 | -0.00886 |
| FABP4 | 0.093571 | -0.01526 | 0.036704 | -0.04348 | 0.004998 |
| BLM.hydrolase | 0.093539 | 0.057842 | 0.000653 | 0.009926 | 0.068508 |
| t.PA | 0.09301 | 0.03777 | 0.008988 | 0.005864 | 0.026824 |
| CCL16 | 0.092745 | 0.025525 | 0.024258 | 0.008159 | -0.00776 |
| ST2 | 0.092377 | 0.018033 | 0.032394 | -0.02809 | 0.050903 |
| CPA1 | 0.090165 | 0.032626 | 0.011596 | -0.00208 | -0.02189 |
| DLK.1 | 0.089327 | 0.049241 | 0.006725 | 0.014681 | -0.04291 |
| CPB1 | 0.088869 | 0.036596 | 0.020839 | -0.01075 | -0.02173 |
| vWF | 0.088302 | 0.011685 | 0.010824 | 0.008518 | 0.015069 |
| IGFBP.2 | 0.088056 | 0.003822 | 0.054872 | -0.02907 | -0.0035 |
| PAI | 0.087567 | 0.023811 | -0.11018 | 0.057114 | -0.05069 |
| COL1A1 | 0.086478 | 0.053625 | 0.005555 | 0.027037 | -0.01217 |
| PRTN3 | 0.086213 | -0.00316 | -0.11615 | -0.04369 | 0.042237 |
| MMP.3 | 0.085116 | 0.053396 | 0.009528 | 0.010496 | 0.017649 |
| LDL.receptor | 0.084269 | 0.060262 | -0.03185 | 0.047405 | -0.01688 |
| CHI3L1 | 0.084087 | -0.01811 | 0.038958 | -0.04503 | 0.008778 |
| MB | 0.079597 | 0.040471 | 0.033986 | 0.007697 | 0.008627 |
| MMP.9 | 0.079375 | -0.00933 | -0.1428 | -0.00349 | 0.01544 |
| PSP.D | 0.078989 | 0.038296 | 0.02606 | 0.003333 | 0.021836 |
| CASP.3 | 0.077146 | 0.017077 | -0.1086 | -0.05206 | 0.154062 |
| SCGB3A2 | 0.074439 | 0.033718 | 0.006211 | -0.0153 | -0.01373 |
| MPO | 0.072504 | -0.01304 | -0.13535 | -0.00802 | 0.032075 |
| TR | 0.068423 | -0.00333 | 0.014095 | -0.03215 | 0.056927 |
| CCL24 | 0.066912 | 0.051916 | 0.006013 | 0.012914 | -0.0077 |
| IGFBP.1 | 0.064898 | 0.011513 | 0.039894 | -0.02837 | 0.000856 |
| PON3 | 0.06481 | 0.110597 | -0.03046 | 0.063788 | -0.01451 |
| CHIT1 | 0.063132 | 0.009206 | -0.00164 | -0.00755 | -0.01548 |
| AZU1 | 0.060648 | -0.01868 | -0.15723 | -0.03598 | 0.049697 |
| Ep.CAM | 0.059458 | 0.057679 | -0.0056 | 0.050786 | 0.018656 |
| CCL22 | 0.058741 | 0.039899 | -0.02654 | -1.8E-05 | -0.05933 |
| TRAIL.R2 | 0.05366 | -0.12313 | 0.079742 | -0.03531 | 0.015771 |
| IL.10RB | 0.052979 | -0.08544 | 0.029213 | -0.03187 | -0.08255 |
| TNFRSF10A | 0.050118 | -0.11875 | 0.074368 | 0.022198 | 0.021663 |
| CD40 | 0.049565 | -0.11431 | -0.01171 | -0.0612 | -0.06865 |
| Gal.9 | 0.049215 | -0.12008 | 0.051661 | 0.004263 | 0.007799 |
| CDCP1 | 0.048449 | -0.05995 | 0.073585 | -0.04535 | -0.01697 |
| TNFRSF9 | 0.04503 | -0.09428 | 0.048851 | -0.08677 | -0.11532 |
| IL.4RA | 0.044722 | -0.11469 | 0.061625 | 0.015649 | 0.059909 |
| uPA.2 | 0.043989 | -0.0622 | 0.006062 | -0.01739 | -0.0634 |
| TNFRSF11A | 0.04376 | -0.1236 | 0.022572 | 0.024831 | -0.04292 |
| PAR.1 | 0.043646 | -0.11369 | 0.012793 | 0.045686 | 0.042574 |
| CD5 | 0.043622 | -0.06626 | 0.015721 | -0.04372 | -0.12477 |
| VSIG2 | 0.043061 | -0.08895 | 0.072929 | 0.027416 | 0.009573 |
| hOSCAR | 0.042831 | -0.08878 | -0.00557 | 0.109725 | -0.01068 |
| CTSL1 | 0.042775 | -0.10631 | 0.069375 | 0.05916 | 0.062465 |
| HGF | 0.042336 | -0.11005 | -0.07735 | -0.08527 | -0.02402 |
| IL.15RA | 0.041767 | -0.05451 | 0.036154 | -0.05312 | -0.11393 |
| SPON2 | 0.041671 | -0.12016 | 0.036071 | 0.062031 | 0.020787 |
| THBS2 | 0.041409 | -0.08395 | 0.072482 | 0.084771 | 0.063877 |
| CSF.1 | 0.041386 | -0.10541 | 0.032182 | -0.08553 | -0.12018 |
| PD.L1 | 0.041273 | -0.07681 | 0.014718 | -0.02208 | -0.09573 |
| MMP.7 | 0.041154 | -0.10192 | 0.063323 | 0.024996 | -0.00873 |
| DCN | 0.040852 | -0.08648 | 0.096539 | 0.122375 | 0.070866 |
| PD.L2 | 0.040675 | -0.08578 | 0.038035 | 0.102394 | 0.030926 |
| TM | 0.040562 | -0.07291 | 0.015565 | 0.12914 | -0.01448 |
| ADM | 0.040217 | -0.11209 | 0.110344 | -0.02178 | 0.04026 |
| CXCL9 | 0.040124 | -0.08531 | 0.062325 | -0.08464 | -0.07599 |
| PRELP | 0.039475 | -0.09469 | 0.087032 | 0.103942 | 0.069552 |
| PIgR | 0.039429 | -0.07533 | 0.042213 | 0.11429 | -0.02187 |
| OPG.y | 0.039421 | -0.07927 | 0.059011 | -0.0352 | -0.04712 |
| IL.1ra | 0.039333 | -0.0953 | -0.08031 | -0.0286 | 0.044431 |
| MERTK | 0.038996 | -0.07823 | 0.070761 | 0.087488 | 0.060526 |
| PRSS8 | 0.038896 | -0.07905 | 0.023108 | 0.05849 | 0.003805 |
| PlGF | 0.038822 | -0.09534 | 0.059369 | 0.006618 | -0.00584 |
| MMP.12 | 0.037689 | -0.07425 | 0.054662 | 0.004645 | -0.04351 |
| SLAMF1 | 0.037659 | -0.0683 | 0.029758 | -0.05213 | -0.07648 |
| TF | 0.037228 | -0.0632 | 0.079185 | 0.126079 | 0.043101 |
| TNFRSF13B | 0.037124 | -0.09194 | 0.058242 | 0.012699 | 0.002271 |
| XCL1 | 0.036997 | -0.08608 | 0.061937 | 0.040128 | -0.0111 |
| VEGF.A | 0.036798 | -0.09775 | -0.02624 | -0.0207 | -0.08687 |
| SLAMF7 | 0.036652 | -0.0866 | 0.046556 | 0.052419 | 0.027588 |
| CCL25 | 0.036504 | -0.06279 | 0.051552 | -0.03158 | -0.05212 |
| CD244 | 0.036381 | -0.06288 | -0.02713 | 0.031377 | -0.12883 |
| IL16 | 0.036341 | -0.09513 | -0.08299 | -0.01475 | 0.055343 |
| LIF.R | 0.036093 | -0.08921 | 0.079666 | -0.0594 | 0.001752 |
| AMBP | 0.035543 | -0.06722 | 0.036499 | 0.164197 | -0.00848 |
| CCL19 | 0.03544 | -0.05879 | 0.022775 | -0.07317 | -0.07789 |
| TIM | 0.035062 | -0.06768 | 0.09788 | 0.024882 | 0.016834 |
| SORT1 | 0.034864 | -0.09142 | -0.10473 | 0.159846 | 0.013616 |
| MCP.3 | 0.034698 | -0.07647 | -0.05218 | -0.05839 | -0.0756 |
| IL.18.y | 0.034548 | -0.04518 | -0.00789 | -0.02548 | -0.0736 |
| Beta.NGF | 0.033639 | -0.05659 | -0.00141 | 0.001742 | -0.04685 |
| FGF.5 | 0.033499 | -0.04274 | 0.043888 | -0.01445 | -0.091 |
| IL.8 | 0.03324 | -0.08116 | -0.01653 | -0.03033 | -0.05449 |
| gal3 | 0.032208 | -0.05609 | 0.019104 | -0.08398 | 0.073491 |
| IL27 | 0.031977 | -0.08861 | 0.095149 | 0.015492 | 0.038812 |
| IL.6.x | 0.031951 | -0.09136 | 0.057728 | -0.09013 | 0.050712 |
| IL.18R1 | 0.031856 | -0.075 | -0.00171 | -0.02638 | -0.05463 |
| MARCO | 0.031792 | -0.06833 | 0.012083 | 0.184742 | 0.026475 |
| IL.18.x | 0.031197 | -0.05697 | -0.00165 | 0.03201 | -0.02444 |
| CCL3 | 0.031139 | -0.07765 | -0.00907 | -0.00682 | -0.00825 |
| MIP.1.alpha | 0.031105 | -0.06845 | -0.01088 | -0.06162 | -0.0507 |
| MMP.10 | 0.030931 | -0.03425 | -0.00606 | 0.006792 | -0.08588 |
| OSM | 0.030711 | -0.08031 | -0.15836 | -0.05135 | -0.00225 |
| IL.6.y | 0.030691 | -0.08507 | 0.052689 | -0.12538 | 0.005191 |
| RAGE | 0.030336 | -0.05797 | 0.063804 | 0.055962 | 0.013966 |
| PTX3 | 0.029878 | -0.08821 | -0.07658 | -0.01534 | 0.092171 |
| FGF.23.y | 0.029702 | -0.09466 | 0.064891 | -0.0958 | 0.040108 |
| FGF.23.x | 0.029077 | -0.10038 | 0.075382 | -0.06007 | 0.060632 |
| CD4 | 0.028739 | -0.10472 | 0.087977 | 0.031675 | 0.011652 |
| IL.12B | 0.02865 | -0.06644 | 0.04269 | -0.06106 | -0.11894 |
| CCL4 | 0.028475 | -0.07675 | -0.06926 | -0.04192 | -0.08947 |
| IL.17C | 0.028351 | -0.04953 | 0.00545 | -0.04611 | -0.06445 |
| CXCL10 | 0.028113 | -0.09178 | 0.042941 | -0.08432 | -0.06106 |
| GT | 0.027793 | -0.02661 | 0.015766 | 0.052524 | 0.009411 |
| ACE2 | 0.027569 | -0.06717 | 0.066374 | 0.04565 | 0.087939 |
| TNFSF14 | 0.027546 | -0.08181 | -0.18327 | -0.04699 | 0.011011 |
| CEACAM8 | 0.027451 | -0.07956 | -0.15078 | -0.00114 | 0.053329 |
| CX3CL1 | 0.026969 | -0.04672 | 0.044836 | -0.03975 | -0.05257 |
| TGF.alpha | 0.026863 | -0.07848 | -0.15262 | -0.06342 | -0.00814 |
| DECR1 | 0.026832 | -0.06516 | -0.06189 | -0.00598 | 0.110721 |
| AGRP | 0.02669 | -0.08934 | 0.063048 | 0.023125 | 0.041381 |
| MCP.1.y | 0.026356 | -0.06066 | -0.04196 | -0.0153 | -0.1361 |
| CCL23 | 0.025733 | -0.07271 | 0.039395 | -0.10913 | -0.06236 |
| PARP.1 | 0.025724 | -0.06604 | -0.07835 | -0.06734 | 0.06514 |
| CA5A | 0.025107 | -0.05916 | 0.042815 | 0.024027 | 0.05719 |
| TIE2 | 0.024964 | -0.08105 | 0.013282 | 0.120125 | 0.078607 |
| CCL11 | 0.02493 | -0.04956 | -0.03457 | 0.022709 | -0.12934 |
| CST5 | 0.024191 | -0.06592 | 0.055235 | -0.02142 | -0.04958 |
| FABP2 | 0.024117 | -0.03032 | 0.03461 | 0.070953 | -0.00208 |
| CCL20 | 0.024054 | -0.06972 | 0.043935 | -0.04772 | -0.01764 |
| HSP.27 | 0.024046 | -0.08348 | 0.013229 | -0.018 | 0.164759 |
| IL.17D | 0.023943 | -0.05784 | 0.070217 | 0.068263 | 0.032544 |
| FGF.21.1 | 0.023607 | -0.07283 | 0.043109 | -0.08426 | -0.00775 |
| FGF.21 | 0.023577 | -0.07843 | 0.049187 | -0.0525 | 0.018966 |
| VEGF.D | 0.023441 | -0.04444 | 0.061322 | 0.094413 | 0.071736 |
| CXCL11 | 0.023374 | -0.09228 | -0.03186 | -0.0723 | -0.06972 |
| Protein.BOC | 0.022673 | -0.05196 | 0.033113 | 0.175324 | 0.071397 |
| TRAIL | 0.021698 | -0.00308 | -0.04143 | 0.043313 | -0.1201 |
| IL.10 | 0.021586 | -0.05167 | 0.00621 | -0.1072 | -0.02086 |
| ADA | 0.021446 | -0.02898 | -0.02006 | -0.03448 | 0.019043 |
| GDNF | 0.021379 | -0.06513 | 0.051767 | -0.03154 | -0.0655 |
| CCL17 | 0.02111 | -0.07173 | -0.08277 | 0.012954 | -0.10321 |
| GDF.2 | 0.020699 | -0.02605 | 0.00506 | 0.188768 | 0.019004 |
| IDUA | 0.020573 | -0.05392 | -0.05882 | 0.096141 | -0.01258 |
| BNP | 0.020473 | -0.08025 | 0.099252 | -0.06113 | 0.072344 |
| LEP | 0.020279 | -0.03611 | -0.00546 | 0.021931 | -0.00321 |
| LOX.1 | 0.020222 | -0.07421 | -0.15979 | -0.01077 | 0.049876 |
| GIF | 0.020027 | -0.03041 | 0.040952 | 0.064825 | 0.000681 |
| THPO | 0.019337 | -0.03773 | -0.03119 | 0.142738 | 0.029189 |
| NEMO | 0.019333 | -0.07967 | -0.14018 | -0.0474 | 0.17325 |
| CASP.8 | 0.019267 | -0.07436 | -0.10382 | -0.13127 | 0.13602 |
| CD84 | 0.018859 | -0.0713 | -0.17428 | 0.096702 | -0.00626 |
| REN | 0.018675 | -0.05182 | 0.018193 | 0.0011 | 0.011094 |
| STK4 | 0.018544 | -0.07259 | -0.15563 | -0.05695 | 0.160565 |
| CCL28 | 0.018424 | -0.04975 | 0.011737 | -0.02644 | -0.05027 |
| EN.RAGE | 0.01828 | -0.0521 | -0.14188 | -0.08901 | 0.044266 |
| SOD2 | 0.017755 | -0.05495 | -0.00756 | 0.136205 | 0.081151 |
| ST1A1 | 0.017669 | -0.04686 | -0.15174 | -0.01386 | 0.070826 |
| PSGL.1 | 0.017576 | -0.02254 | 0.024128 | 0.159702 | 0.052402 |
| Flt3L | 0.017475 | -0.0354 | 0.054198 | -0.01779 | -0.06772 |
| FS | 0.017422 | -0.04779 | 0.035889 | 0.047982 | 0.008908 |
| IL1RL2 | 0.017412 | -0.03201 | 0.014072 | 0.0969 | 0.029967 |
| ANG.1 | 0.017007 | -0.05832 | -0.12611 | 0.169058 | -0.03919 |
| PAPPA | 0.016402 | -0.0494 | 0.002679 | 0.084043 | 0.039987 |
| IL.7 | 0.016123 | -0.0439 | -0.05039 | 0.015244 | -0.116 |
| IgG.Fc.receptor.II.b | 0.016066 | -0.0404 | 0.000961 | 0.056345 | 0.031695 |
| STAMPB | 0.01606 | -0.04865 | -0.06648 | -0.08211 | 0.160897 |
| Dkk.1 | 0.015485 | -0.06068 | -0.157 | 0.141716 | -0.05844 |
| ITGB1BP2 | 0.015438 | -0.04105 | -0.04521 | -0.06137 | 0.113729 |
| HO.1 | 0.015362 | -0.04639 | 0.053122 | 0.064627 | 0.056832 |
| PRSS27 | 0.014745 | -0.04651 | -0.07825 | 0.122046 | -0.09796 |
| NRTN | 0.014579 | -0.0079 | -0.01459 | -0.00433 | -0.01755 |
| MCP.4 | 0.014118 | -0.05332 | -0.05501 | -0.01692 | -0.11672 |
| SIRT2 | 0.014018 | -0.05504 | -0.0707 | -0.10106 | 0.175351 |
| HAOX1 | 0.013944 | -0.02949 | 0.013928 | 0.036921 | 0.061764 |
| NT.3 | 0.013924 | -0.02599 | 0.003711 | 0.042057 | -0.03863 |
| AXIN1 | 0.013468 | -0.06606 | -0.1199 | -0.08908 | 0.12978 |
| ADAM.TS13 | 0.013377 | -0.02921 | -0.02336 | 0.24031 | 0.030748 |
| IL.1.alpha | 0.013221 | -0.01594 | 0.004959 | 0.024865 | 0.012939 |
| PDGF.subunit.B | 0.013146 | -0.05495 | -0.12501 | 0.128124 | -0.09224 |
| CD40.L | 0.012975 | -0.06644 | -0.17755 | 0.049251 | -0.01201 |
| SERPINA12 | 0.012867 | -0.01875 | 0.013783 | 0.017291 | 0.0242 |
| TGM2 | 0.012667 | -0.0565 | -0.00607 | 0.009732 | 0.203576 |
| MMP.1 | 0.012138 | -0.04961 | -0.04185 | -0.05796 | -0.07207 |
| TNF | -0.01176 | -0.00415 | -0.01626 | -0.021 | 0.013189 |
| X4E.BP1 | 0.011613 | -0.06184 | 0.008679 | -0.1204 | 0.143366 |
| LAP.TGF.beta.1 | 0.011398 | -0.07293 | -0.12233 | -0.01394 | -0.10169 |
| IL.17A | 0.01059 | -0.02728 | -0.0042 | -0.02809 | -0.06193 |
| HB.EGF | 0.010494 | -0.07565 | -0.09606 | 0.079511 | -0.05967 |
| TWEAK | 0.010438 | -0.00755 | -0.10064 | 0.08419 | -0.139 |
| SRC | 0.010017 | -0.05803 | -0.1629 | 0.006639 | 0.11096 |
| GH | 0.009648 | -0.02592 | 0.037319 | -0.00159 | 0.021852 |
| CXCL1.x | 0.009608 | -0.08073 | -0.08727 | 0.010062 | -0.04545 |
| FGF.19 | 0.0093 | -0.03399 | 0.021317 | -0.00099 | -0.05521 |
| TNFB | 0.009276 | -0.03142 | -0.00428 | 0.001609 | -0.09244 |
| CTRC | 0.008904 | -0.01568 | 0.002026 | 0.083979 | -0.01019 |
| CXCL1.y | 0.008877 | -0.07335 | -0.09254 | -0.03517 | -0.07395 |
| CXCL6 | 0.008307 | -0.07017 | -0.08826 | -0.02764 | -0.08136 |
| MCP.2 | 0.007772 | -0.04705 | -0.07763 | 0.024508 | -0.0984 |
| CXCL5 | 0.006942 | -0.02604 | -0.06744 | 0.041407 | -0.11901 |
| TSLP | -0.00688 | -0.00211 | -0.00103 | -0.00594 | 0.001261 |
| IL.24 | 0.006399 | -0.00407 | 0.005549 | -0.00642 | -0.03091 |
| TRANCE | -0.00625 | 0.015154 | -0.0412 | 0.032778 | -0.07533 |
| CD6 | 0.006063 | -0.01268 | 0.000863 | 0.023368 | -0.0844 |
| trop | 0.005934 | -0.03233 | 0.044769 | -0.02648 | 0.053578 |
| LIF | -0.00564 | -0.00467 | -0.00844 | 0.017601 | 0.026313 |
| DNER | -0.00554 | 0.021361 | -0.05792 | 0.105885 | -0.05926 |
| LPL | 0.005352 | -0.0354 | 0.03535 | 0.072545 | 0.014742 |
| IL.10RA | 0.005324 | -0.00809 | -0.02392 | -0.0188 | -0.02719 |
| IL.5 | 0.005225 | 0.0085 | -0.03583 | -0.0247 | -0.0184 |
| IL.4 | 0.004708 | -0.00937 | -0.00295 | -0.03913 | -0.0419 |
| IL.2 | 0.004374 | -0.01781 | -0.03677 | 0.03407 | -0.02644 |
| ARTN | -0.00408 | -0.00848 | 0.018535 | -0.02134 | -0.03538 |
| GLO1 | 0.003638 | -0.04171 | -0.03236 | -0.02806 | 0.191325 |
| IL.20RA | -0.00223 | -0.00218 | -0.00696 | 0.01776 | -0.01489 |
| IL.33 | 0.002028 | -0.01497 | -0.01017 | 0.003483 | -0.03472 |
| SCF.x | -0.00181 | 0.00574 | -0.00189 | 0.123753 | -0.01964 |
| IL.22.RA1 | 0.001532 | -0.00885 | -0.03007 | 0.041059 | -0.05178 |
| IL.2RB | -0.0015 | 0.003906 | -0.01088 | 0.013628 | -0.01368 |
| SCF.y | -0.00109 | 0.024936 | -0.01046 | 0.066826 | -0.07878 |
| IFN.gamma | 0.000909 | -0.01648 | -0.02528 | 0.004497 | -0.00468 |
| IL.13 | -0.0007 | -0.01506 | -0.00908 | -0.02897 | 0.002349 |
| BDNF | -0.00058 | 0.018953 | -0.02056 | -0.02313 | -0.03534 |
| IL.20 | 0.000345 | -0.0113 | 0.020711 | 0.010425 | 0.040491 |

**Supplementary Table 10: Principal component analysis loadings (Eigenvectors) across the 6 selected principal components from the CMR analysis**

Variables are sorted according to the absolute magnitude of their contribution towards the first principal component, following normalization. Green and red shadings indicate positive and negative contributions, respectively. The strength of shading indicates the absolute magnitude of contribution.

|  | PC1 | PC2 | PC3 | PC4 | PC5 | PC6 |
| --- | --- | --- | --- | --- | --- | --- |
| lvesvi | 0.422575 | 0.063725 | 0.298969 | -0.19745 | 0.086925 | -0.26081 |
| lvef | -0.41307 | 0.236253 | -0.17752 | 0.113759 | -0.07078 | 0.163826 |
| rvef | -0.37326 | 0.109968 | 0.427998 | -0.09855 | -0.06925 | -0.15438 |
| lvedvi | 0.360703 | 0.242509 | 0.342678 | -0.22575 | 0.056998 | -0.28189 |
| rvesvi | 0.357068 | 0.216783 | -0.43363 | 0.073408 | 0.159449 | 0.049815 |
| lvmi | 0.259448 | 0.133321 | 0.378893 | -0.01346 | -0.08287 | 0.870699 |
| lavi | 0.217284 | 0.131806 | -0.09341 | 0.174931 | -0.92884 | -0.13937 |
| rvedvi | 0.211473 | 0.473338 | -0.352 | 0.055466 | 0.190998 | 0.011696 |
| rvsvi | -0.21135 | 0.532687 | 0.073472 | -0.01988 | 0.092018 | -0.06416 |
| lvsvi | -0.20362 | 0.527156 | 0.122573 | -0.06764 | -0.08021 | -0.02232 |
| lge | 0.092174 | 0.037145 | 0.306355 | 0.918249 | 0.181576 | -0.13066 |

References

1. Tayal U, Wage R, Newsome S et al. Predictors of left ventricular remodelling in patients with dilated cardiomyopathy - a cardiovascular magnetic resonance study. Eur J Heart Fail 2020;22:1160-1170.

2. Whiffin N, Walsh R, Govind R et al. CardioClassifier: disease- and gene-specific computational decision support for clinical genome interpretation. Genet Med 2018;20:1246-1254.

3. Assarsson E, Lundberg M, Holmquist G et al. Homogenous 96-plex PEA immunoassay exhibiting high sensitivity, specificity, and excellent scalability. PLoS One 2014;9:e95192.

4. Shah SJ, Katz DH, Selvaraj S et al. Phenomapping for novel classification of heart failure with preserved ejection fraction. Circulation 2015;131:269-279.

5. Hicks KA, Tcheng JE, Bozkurt B et al. 2014 ACC/AHA Key Data Elements and Definitions for Cardiovascular Endpoint Events in Clinical Trials: A Report of the American College of Cardiology/American Heart Association Task Force on Clinical Data Standards (Writing Committee to Develop Cardiovascular Endpoints Data Standards). Circulation 2015;132:302-361.

6. Zipes DP, Camm AJ, Borggrefe M et al. ACC/AHA/ESC 2006 guidelines for management of patients with ventricular arrhythmias and the prevention of sudden cardiac death--executive summary: A report of the American College of Cardiology/American Heart Association Task Force and the European Society of Cardiology Committee for Practice Guidelines (Writing Committee to Develop Guidelines for Management of Patients with Ventricular Arrhythmias and the Prevention of Sudden Cardiac Death) Developed in collaboration with the European Heart Rhythm Association and the Heart Rhythm Society. Eur Heart J 2006;27:2099-2140.

7. de Marvao A, Dawes TJ, Shi W et al. Precursors of Hypertensive Heart Phenotype Develop in Healthy Adults: A High-Resolution 3D MRI Study. JACC Cardiovasc Imaging 2015;8:1260-1269.

8. Pocock SJ, Ariti CA, McMurray JJ et al. Predicting survival in heart failure: a risk score based on 39 372 patients from 30 studies. Eur Heart J 2013;34:1404-1413.

9. Bartekova M, Radosinska J, Jelemensky M, Dhalla NS. Role of cytokines and inflammation in heart function during health and disease. Heart Fail Rev 2018;23:733-758.
